# Catch reconstructions reveal that global marine fisheries catches are higher than reported and declining

**DOI:** 10.1038/ncomms10244

**Published:** 2016-01-19

**Authors:** Daniel Pauly, Dirk Zeller

**Affiliations:** 1 Sea Around Us, Global Fisheries Cluster, University of British Columbia, 2202 Main Mall, Vancouver, British Columbia, Canada V6T 1Z4

## Abstract

Fisheries data assembled by the Food and Agriculture Organization (FAO) suggest that global marine fisheries catches increased to 86 million tonnes in 1996, then slightly declined. Here, using a decade-long multinational ‘catch reconstruction' project covering the Exclusive Economic Zones of the world's maritime countries and the High Seas from 1950 to 2010, and accounting for all fisheries, we identify catch trajectories differing considerably from the national data submitted to the FAO. We suggest that catch actually peaked at 130 million tonnes, and has been declining much more strongly since. This decline in reconstructed catches reflects declines in industrial catches and to a smaller extent declining discards, despite industrial fishing having expanded from industrialized countries to the waters of developing countries. The differing trajectories documented here suggest a need for improved monitoring of all fisheries, including often neglected small-scale fisheries, and illegal and other problematic fisheries, as well as discarded bycatch.

## Abstract (web summary)

Officially reported fisheries statistics suggest that global catches have stabilized since their peak in the mid-1990s. Here, the authors supplement these reported data with best-estimate values of missing data from the literature, and find that global catches have steadily declined over this time period.

## Graphical abstract

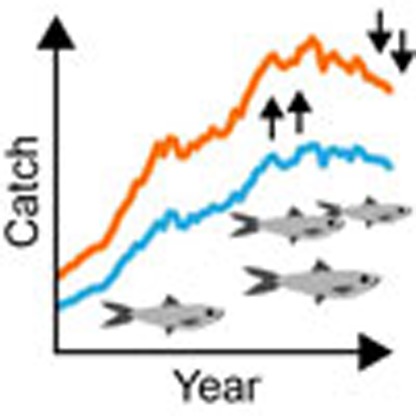

Marine fisheries are the chief contributors of wholesome seafood (finfish and marine invertebrates; here ‘fish'). In many developing countries (and likely also in many ‘transition‘ countries), fish is the major animal protein source that rural people can access or afford^1^; and they are also an important source of micronutrients essential to people with otherwise deficient nutrition^2^. However, the growing popularity of fish in countries with developed or rapidly developing economies creates a demand that cannot be met by fish stocks in their own waters (for example, the EU, the USA, China and Japan). These markets are increasingly supplied by fish imported from developing countries, or caught in the waters of developing countries by various distant-water fleets^3^,^4^,^5^, with the consequences that:

- Foreign and/or export-oriented domestic industrial fleets are increasingly fishing in the waters of developing countries^5^,^6^,
- Industrially caught fish has become a globalized commodity that is mostly traded between continents rather than consumed in the countries where it was caught^7^, and
- The small-scale fisheries that traditionally supplied seafood to coastal rural communities and the interior of developing countries (notably in Africa)^8^ are forced to compete with the export-oriented industrial fleets without much support from their governments.

The lack of attention that small-scale fisheries suffer in most parts of the world^9^ manifests itself in potentially misleading statistics that are submitted annually by many member countries of the Food and Agriculture Organization of the United Nations (FAO), which may omit or substantially underreport small-scale fisheries data^10^. FAO harmonizes the data submitted by its members, which then becomes the only global data set of fisheries statistics in the world, widely used by policy makers and scholars^11^.

This data set, however, may not only underestimate artisanal (that is, small scale, commercial) and subsistence fisheries^10^, but also generally omit the catch of recreational fisheries, discarded bycatch^12^ and illegal and otherwise unreported catch, even when some estimates are available^13^. Thus, except for a few obvious cases of over-reporting^14^, the landings data updated and disseminated annually by the FAO on behalf of member countries may considerably underestimate actual fisheries catch. While this underestimation is widely known among many fisheries scientists working with FAO catch data, and is freely acknowledged by FAO, its global magnitude has not been explicitly presented until now.

Here we present the results of an approach called ‘catch reconstruction'^15^,^16^ that utilizes a wide variety of data and information sources to derive estimates for all fisheries components missing from the official reported data. We find that reconstructed global catches between 1950 and 2010 were 50% higher than data reported to FAO suggest, and are declining more strongly since catches peaked in the 1990s. These findings and the country-specific technical work underlying these results will hopefully contribute to member countries submitting more accurate fisheries statistics to FAO. Such improved and more comprehensive data contribute a foundation that can facilitate the implementation of ecosystem-based fisheries management^17^, which is a component of the ‘*FAO Code of Conduct for Responsible Fisheries*'^18^.

## Results

### Global pattern

**Figure 1:**
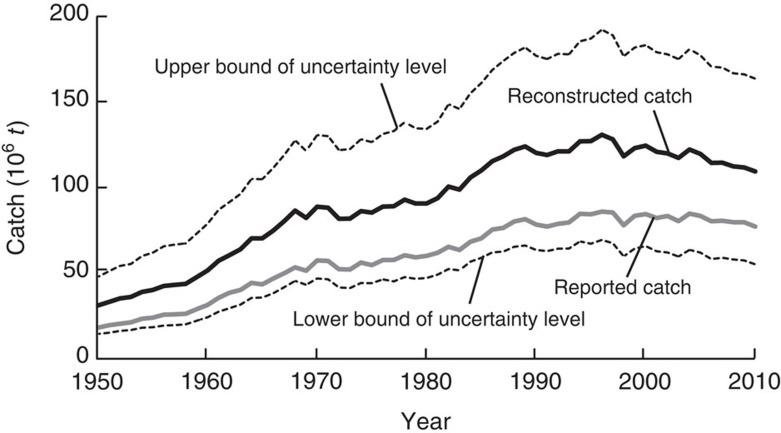
Trajectories of reported and reconstructed marine fisheries catches 1950–2010. Contrast between the world's marine fisheries catches, assembled by FAO from voluntary submissions of its member countries (‘reported') and that of the catch ‘reconstructed' to include all fisheries known to exist, in all countries and in the High Sea (‘reconstructed'=‘reported'+estimates of ‘unreported'). The mean weighted percentage uncertainty of the reconstructed total catches (over all countries and fisheries sectors) based on the quality scores attributed to each sector in each country and territory (dashed line) is also shown.

The sum of the reconstructed catches of all sectors in all Exclusive Economic Zones (EEZs) of the world, plus the catch of tuna and other large pelagic fishes in the High Seas leads to two major observations (Fig. 1; Supplementary Table 1). First, the trajectory of reconstructed catches differs substantially from those reported by FAO on behalf of its member countries. The FAO statistics suggest that, starting in 1950, the world catch (actually ‘landings', as discarded catches are explicitly excluded from the global FAO data set) increased fairly steadily to 86 million tonnes (mt) in 1996, stagnated and then slowly declined to around 77 mt by 2010 (Fig. 1). In contrast, the reconstructed catch peaked at 130 mt in 1996 and declined more strongly since. Thus, the reconstructed catches are overall 53% higher than the reported data.

Furthermore, since the year of peak catches in 1996, the reconstructed catch declined strongly at a mean rate of −1.22 mt·per year, whereas FAO, at least until 2010, described the reported catch cautiously as characterized by ‘stability'^19^,^20^, though it exhibited a gradual decline (−0.38 mt·per year). The reconstructed total catches therefore represent a decline of over three times that of the reported data as presented by FAO on behalf of countries. A segmented regression^21^ identifies two breakpoints in the catch time series (that is, change in trend) of the reconstructed total catches as well as the reported catches. These are in 1967 as a result of a changing slope of the catch time series from a stronger increase prior to 1967 (reconstructed catches=2.82 mt·per year; reported catches=1.88 mt·per year) to a slower increase after 1967 (reconstructed catches=1.86 mt·per year; reported catches=1.30 mt·per year). The second breakpoint is in 1996 (the year of peak catch), with a subsequently decreasing trend (that is, slope) of −1.22 mt·per year for reconstructed catches and −0.38 mt per·year for reported catches, as also presented for the simple regression above (Fig. 1; see also Supplementary Table 2).

**Figure 2:**
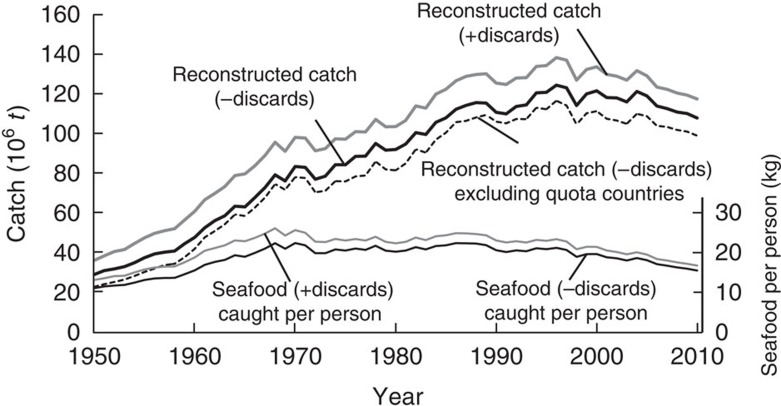
Trajectories of marine fisheries catches 1950–2010. Effects of removing discards on estimates of seafood caught *per capita*, and of removing the catches of the major countries using quota management (that is, USA, New Zealand, Australia and Western Europe) on reconstructed total catches.

Note that the recent, stronger decline in reconstructed total catches is not due to some countries reducing catch quotas so that stocks can rebuild. For example, a similar decline (−1.01 mt·per year) in reconstructed catches is obtained when the catch from the Unites States, Northwestern Europe, Australia and New Zealand (that is, countries where quota management predominates) is excluded (Fig. 2; Supplementary Table 3).

### Spatial pattern

**Figure 3:**
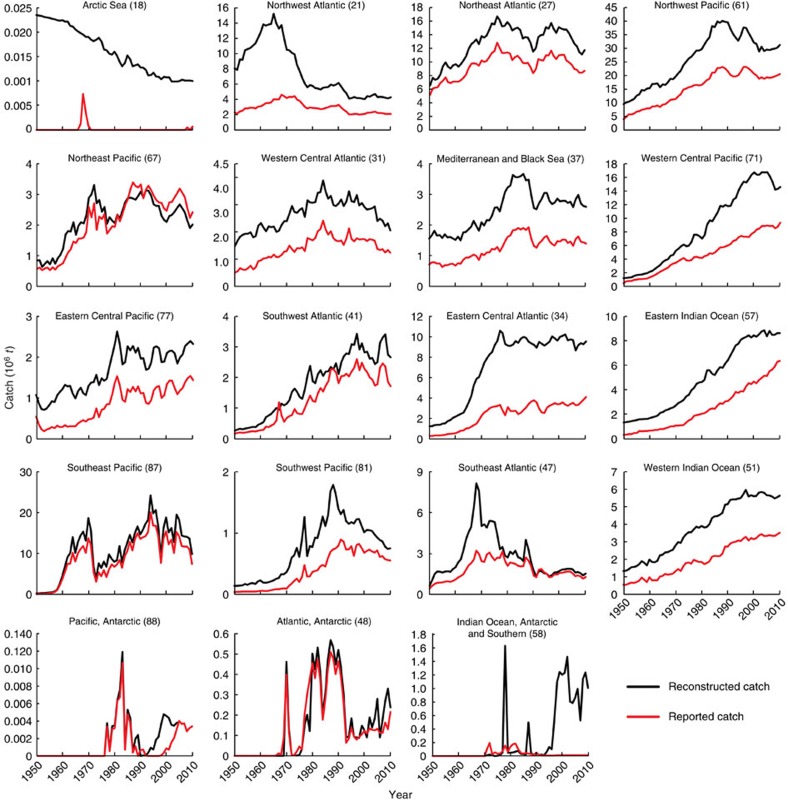
Reconstructed and reported catches by FAO areas. Contrasting reconstructed and reported catches in the 19 maritime ‘Statistical Areas' which FAO uses to roughly spatialize the world catch. Note that for Area 18 (Arctic), the reported catch by the U.S. and Canada was zero, while only Russia (former-USSR) reported a small catch in the late 1960s, even though the coastal fishes of the high Arctic are exploited by Inuit and others.

Closer examination of the reconstructed versus reported catches in each of the 19 maritime FAO statistical areas suggests that some of the areas where industrial fishing originated, such as the Northwest Atlantic (FAO area 21), are the first regions of the world to demonstrate declining catches (Fig. 3). In contrast, lower-latitude areas demonstrate declines later, or still appear to have increasing catches, for example, the Indian and Western Central Pacific Oceans still showing generally increasing trends in reported catches (Fig. 3).

### Catches by fishing sector

**Figure 4:**
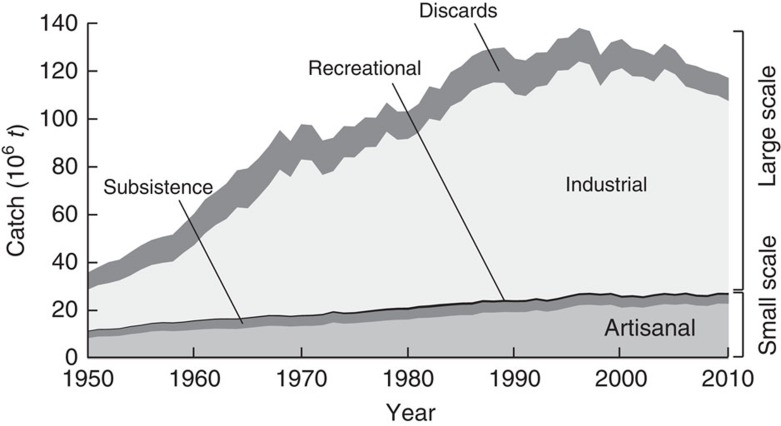
Reconstructed global catch by fisheries sectors. Reconstructed catches for all countries in the world, plus High Seas, by large-scale (industrial) and small-scale sectors (artisanal, subsistence, recreational), with discards (overwhelmingly from industrial fisheries) presented separately.

We present, for the first time, global reconstructed marine fisheries catches by fisheries sectors (Fig. 4; Supplementary Table 4). They are dominated by industrial fisheries, which contribute 73 mt of landings in 2010, down from 87 mt in 2000 (Fig. 4). At the global scale it is a declining industrial catch (combined with the smaller contribution of gradually reduced levels of discarding)^12^ that leads to declining global catches since 1996, while the artisanal sector, which generates a catch increasing from about 8 mt·per year in the early 1950s to 22 mt·per year in 2010, continues to show gradual growth in catches at the global scale (Fig. 4).

Also noticeable is that the inter-annual variations (small peaks and troughs) in both reconstructed catches and reported catches (Fig. 1) are mainly driven by industrial data, which are relatively well documented and reported in time series, while the small-scale sector data are smoother over time (Fig. 4), and more strongly influenced by continuity assumptions over time as part of the national reconstructions.

While some countries increasingly include subsets of artisanal catches in official catch statistics provided to FAO, subsistence fisheries catches (Fig. 4) rarely are^10^. Worldwide, subsistence fisheries caught an estimated 3.8 mt·per year between 2000 and 2010 (Fig. 4; Supplementary Table 4). The current global estimate of just under 1 mt·per year of recreational catches is rather imprecise, and recreational fishing is declining in developed, but increasing in developing countries.

Discarded bycatch, generated mainly by industrial fishing, notably shrimp trawling^22^, was estimated at 27 mt·per year (±10 mt) and 7 mt·per year (±0.7 mt) in global studies conducted for FAO in the early 1990s and 2000s, respectively^23^,^24^. However, these point estimates were not incorporated into FAO's global ‘capture' database, which thus consists only of landings. Here, these studies are used, along with numerous other sources, to generate time series of discards (Fig. 4). Discards, after peaking in the late 1980s, have declined, and during 2000–2010, an average of 10.3 mt·per year of fish were discarded.

## Discussion

Our reconstructed catch data, which combines the data reported to FAO with estimates of unreported catches (that is, reconstructed data are ‘reported FAO data+unreported catches') include estimates of uncertainty (Fig. 1) associated with each national reconstruction. Note that many reconstructions are associated with high uncertainty, especially for earlier decades, for sectors such as subsistence which receive less data collection attention by governments, and for small countries or territories (Fig. 1; Supplementary Table 5)^10^. We include uncertainty estimates here, despite the fact that reconstructions address an inherent negative bias in global catch data (that is, address the ‘accuracy' of data) and not the replicability of catch data collection (that is, the statistical ‘precision' of such estimates), which is what ‘uncertainty' estimates (for example, confidence limits) generally are used for. We do recognize that any estimates of unreported catches implies a certain degree of uncertainty, but so do officially reported data. Most countries in the world use sampling schemes, estimations and raising factors to derive their national catch data they officially report domestically and internationally, all without including estimates of the uncertainty inherent in the numbers being reported as official national catches.

Our comparison of the reconstructed versus reported catches in each of the 19 maritime FAO statistical areas suggests that some of the lower-latitude areas still appear to have increasing reported catches. This generally increasing trend is most pronounced in the Indian and Western Central Pacific Oceans (Fig. 3), where the reconstructed catches are most uncertain, as the statistics of various countries could only partially correct a regional tendency to exaggerate reported catches^5^. FAO's Indian and Western Central Pacific Oceans areas are also the only ones with an increasing FAO reported catch, which, when added to that of other FAO areas, makes the FAO reported world catch appear more stable than it is based on our global reconstructions.

Our data and analyses show that, at the global scale, it is a declining industrial catch (plus a smaller contribution of gradually declining discards)^12^ that provide for the declining global catches, while artisanal fishing continues to show slight growth in catches (Fig. 4). Thus, the gradually increasing incorporation of artisanal and other small-scale catches in the officially reported data presented by FAO on behalf of countries is partly masking the decline in industrial catches at the global level. Since officially reported data are not (at the international level) separated into large-scale versus small-scale sectors^25^, this trend could not be easily documented until now. Obviously, these patterns may vary between countries. Furthermore, while parts of artisanal catches are increasingly included in official catch statistics by some countries, non-commercial subsistence fisheries catches, a substantial fraction of it through gleaning by women in coastal ecosystems such as coral reef flats and estuaries^26^ are generally neglected. The importance of subsistence fishing for the food security of developing countries, particularly in the tropical Indo-Pacific, cannot be overemphasized^10^,^27^.

Our preliminary and somewhat imprecise reconstruction of recreational catches indicates that this sector is largely missing from official reported data, despite FAO's annual data requests explicitly allowing inclusion of recreational catch data. This activity, however, generates an estimated 40 billion USD·per year of global benefits, involves between 55 and 60 million persons, and generates about one million jobs worldwide^28^.

Finally, our country-by-country reconstructed data supports previous studies illustrating that global discards have decreased^12^,^24^. Discarded catches should therefore be included in catch databases, if only to allow for correct inferences on the state of the fisheries involved in this problematic practice.

The reconstructed catch data presented here for the first time for all countries in the world can contribute to formulating better policies for governing the world's marine fisheries, with a first step being the recognition in national policies of the likely magnitude of fisheries not properly captured in the official national data collection systems. This recognition will hopefully contribute to improvements in national data collection systems, an aspiration that we share with FAO. For example, in Mauritania and Guinea Bissau, which, in large part as a result of the reconstructions^29^,^30^ and our ongoing direct engagement with these countries, are now initiating national data collection systems for recreational fisheries (a growth industry in both countries and missing from current data systems). It is hoped that this type of data, and other missing data (for example, subsistence catches)^10^, will be included in future national data reports to FAO, as is the case for some other countries such as Finland^31^. The taxonomic composition of this reconstructed catch (not presented here but available from the *Sea Around Us* and through the individual catch reconstruction reports, see Supplementary Table 5) can also contribute to the development of more useful first-order indicators of fisheries status^32^,^33^,^34^ than has been possible previously, especially in the absence of comprehensive stock assessments for all taxa targeted.

A policy change that would be straightforward for FAO to coordinate and implement with all countries around the world is to request countries to submit their annual catch statistics separately for large-scale and small-scale fisheries^25^, which would be an excellent contribution towards the implementation of the ‘*Voluntary Guidelines for Securing Sustainable Small-scale Fisheries in the Context of Food Security and Poverty Eradication*' recently adopted and endorsed at the thirty-first Session of the FAO Committee on Fisheries and Aquaculture (COFI) in June 2014 (ref. 35). While we have found that many countries already have such data or data structure at hand, until all countries can implement such a data-change request, FAO could incorporate such a split into their internal data harmonization procedures, based, for example, on the same or similar information sources as used by the reconstructions.

The very high catches that were achieved globally in the 1990s were probably not sustainable. However, they do suggest that stock rebuilding, as successfully achieved in many Australian and US fisheries, and beginning to be applied in some European fisheries, is a policy that needs wider implementation, and which would generate even higher sustained benefits than previously estimated from reported catches^36^. On the other hand, the recent catch decline documented here is of considerable concern in its implication for food security, as evidenced by the decline in *per capita* seafood availability (Fig. 2). Note that the recent, strong decline in reconstructed total catches is also evident if catches in countries with well-established quota management systems (United States, Northwestern Europe, Australia and New Zealand) are excluded (Fig. 2). Low quotas are generally not imposed when a stock is abundant; rather low and reduced quotas in fully developed fisheries are generally a management intervention to reduce fishing pressure as a result of past overfishing. Similarly, it has been proposed that strongly declining catches in unmanaged, heavily exploited fisheries are likely a sign of overfishing^32^,^33^,^34^. The often raised suggestion that aquaculture production can replace or compensate for the shortfall in wild capture seafood availability, while being questionable for various reasons^37^, is not addressed here.

The last policy relevant point to be made here transcends fisheries in that it deals with the accuracy of the data used by the international community for its decision making, and the generation of factual knowledge that this requires. After the creation of the United Nations and its technical organizations, including the FAO, a major project of ‘quantifying the world'^38^ began to provide data for national and international agencies on which they could base their policies. As a result, large databases, for example on agricultural crops and forest cover, were created whose accuracy is becoming increasingly important given the expanding exploitation of our natural ecosystems^39^.

Periodic validation of these databases should therefore be a priority to ensure they avoid producing ‘poor numbers'^40^. For example, reports of member countries to FAO about their forest cover, when aggregated at the global level, suggest that the annual rate of forest loss between 1990/2000 and 2001/2005 was nearly halved, while the actual loss rate doubled when assessed by remote sensing and rigorous sampling^41^. Similarly, here we show that the main trend of the world marine fisheries catches is not one of ‘stability' as cautiously suggested earlier by FAO^42^, but one of decline. Moreover, this decline, which began in the mid-1990s, started from a considerably higher peak catch than suggested by the aggregate statistics supplied by FAO members, implying that we have more to lose if this decline continues. Thankfully, this also means that there may be more to gain by rebuilding stocks.

For the global community, a solution could therefore be to provide the FAO the required funds to more intensively assist member countries in submitting better and more comprehensive fishery statistics, especially statistics that cover all fisheries components, and report data by sector^25^. Such improved statistics can then lead to better-informed policy changes for rebuilding stocks and maintaining (sea)food security. Alternatively, or in addition, FAO could team up with other groups (as was done for forestry statistics) to improve the fisheries statistics of member countries that often have fisheries departments with very limited human and financial resources.

Ultimately, the only database of international fisheries statistics that the world has (through FAO) can be improved. The more rapid decline of fisheries catches documented here is a good reason for this.

## Methods

### Catch reconstruction principles

The catch reconstruction approach rests on two basic principles^16^:

- When ‘no data are available' on a fishery that is known to exist, it is not appropriate to enter ‘NA' or ‘no data' into the database. Such entries will later be turned into a zero, which is a bad estimate of the catch of an existing fishery. This concern about the problematic ‘elegance of the number zero' is also something that affects other scientific activities, such as climate modelling^43^;
- Rather, a best estimate should be inserted in all such cases, based on the fact that fishing is a social activity that is bound to throw a ‘shadow' on the society in which it is embedded, and from which an approximate and conservative (but better than zero) estimate of catch can be derived if fishing of this type is known to occur (for example, from the seafood or the fuel consumed locally, or the number of vessels engaged in fisheries and the average catch rate of vessels of this type and so on).

This approach addresses an inherent negative bias in national and, by extension, global catch data, although considerable uncertainty in catch data is likely to remain.

Notably, when doing reconstructions, it became apparent that the perception of ‘no data' being available was not always correct: the ‘social shadow' yields hundreds of articles in the peer-reviewed and report literature with catch data, or data from which catch rates could be inferred, even for remote islands^10^. Also, countries may sometimes send to FAO a stripped down version of the national catch data their fisheries research institutes actually possess, and may even publish on their websites.

What is covered here are both ‘coastal' waters, defined as the waters within the EEZ (Supplementary Fig. 1) that countries have claimed since this was allowed under the United Nations Convention on the Law of the Sea (UNCLOS), or which they could claim under UNCLOS rules, but have not (such as many countries around the Mediterranean), and the open oceans, or High Seas, that is, the waters beyond national jurisdiction (that is, beyond the EEZs). The delineations provided by the Flanders' Marine Institute (VLIZ, see www.vliz.be) are used for our definitions of EEZs. Countries that have not formally claimed an EEZ are assigned areas equivalent to EEZs based on the basic principles of EEZs as outlined in UNCLOS (that is, 200 nm and/or mid-line rules). Note that we (a) include territorial waters within our EEZs; and (b) treat disputed zones (that is, EEZ areas claimed by more than one country) as being ‘owned' by each claimant with respect to their fisheries catches. We treat EEZ areas prior to each country's year of EEZ declaration as ‘EEZ-equivalent waters' (with open access to all fishing countries during that time). If the year of EEZ declaration could not be determined (and for ‘EEZs' that were derived by us for non-claimant countries), we assign the year 1982 as declaration year, that is, the year of conclusion of UNCLOS.

We use different catch reconstruction approaches for EEZs (40% of the global ocean), and High Seas (60%), where the catches are mainly large pelagic fishes (notably tuna). Note that we also exclude the Caspian Sea from all considerations.

### Domestic catch reconstruction method

Reconstructing time series of fisheries catch for all countries of the world from 1950 (the first year that FAO published its ‘Yearbook' of global fisheries statistics) to 2010 was undertaken by fisheries ‘sectors'. However, because a standardized global definition of fishing sectors based on vessel size does not exist (for example, a vessel considered large-scale (industrial) in a developing country may be considered small-scale (artisanal) in developed countries), reconstructions utilize each country's individual definitions for sectors, or a regional equivalent. These are described in each country reconstruction publication underlying this work. We consider four sectors:

- Industrial: large-scale fisheries (using trawlers, purse-seiners, longliners) with high capital input into vessel construction, maintenance and operation, and which may move fishing gear across the seafloor or through the water column using engine power (for example, demersal and pelagic trawlers), irrespective of vessel size. This corresponds to the ‘commercial' sectors of countries such as the USA;
- Artisanal: small-scale fisheries whose catch is predominantly sold (hence they are also ‘commercial fisheries'), and which often use a large variety of generally static or stationary (passive) gears. Our definition of artisanal fisheries relies also on adjacency: they are assumed to operate only in domestic waters (that is, in their country's EEZ). Within their EEZ, they are further limited to a coastal area to a maximum of 50 km from the coast or to 200 m depth, whichever comes first. This area is defined as the Inshore Fishing Area (IFA)^44^. Note that the definition of an IFA assumes the existence of a small-scale fishery, and thus unpopulated islands, although they may have fisheries in their EEZ (which by our definition are industrial, whatever the gear used), have no IFA;
- Subsistence: small-scale non-commercial fisheries whose catch is predominantly consumed by the persons fishing it, and their families (this may also include the ‘take-home' fraction of the catch of commercial fishers, which usually by-passes reporting systems); and
- Recreational: small-scale non-commercial fisheries whose major purpose is enjoyment.

In addition to the reconstructions by sector, we also assign catches to either ‘landings' (that is, retained and landed catch) or ‘discards' (that is, discarded catch), and label all catches as either ‘reported' or ‘unreported' with regards to national and FAO data. Thus, reconstructions present ‘catch' as the sum of ‘landings' plus ‘discards'.

Discarded fish and invertebrates are generally assumed to be dead, except for the US fisheries where the fraction of fish and invertebrates reported to survive is generally available on a per species basis^45^. Due to a distinct lack of global coverage of information, we do not account for so-called under-water discards, or net-mortality of fishing gears^46^. We also do not address mortality caused by ghost-fishing of abandoned or lost fishing gear^47^.

For commercially caught jellyfishes (particularly Rhizostomeae, but also other taxa), it has been shown that over 2.5 time more are caught than reported to FAO (mostly as ‘*Rhizostoma* spp.')^48^. This factor is used to estimate missing catches of unidentified jellyfish. However, this additional catch is, pending further study, not allocated to any specific country or FAO area, and is thus counted only in the world's total catch.

We exclude from consideration all catches of marine mammals, reptiles, corals, sponges and marine plants (the bulk of the plant material is not primarily used for human consumption, but for cosmetic or pharmaceutical use). In addition, we do not estimate catches made for the aquarium trade, which can be substantial in some areas in terms of number of individuals, but relatively small in overall tonnage, as most aquarium fish are small or juvenile specimens^49^.

Most catch reconstructions consist of six steps^15^:

(1) Identification, sourcing and comparison of baseline catch times series, that is, (a) FAO reported landings data by FAO statistical areas, taxon and year; and (b) national or regional data series by area, taxon and year. Implicit in this first step is that the spatial entity be identified and named that is to be reported on (for example, EEZ of Germany in the Baltic Sea), something that is not always obvious, and which poses problems to some of our external collaborators, notably those in countries with a claimed EEZ overlapping with that of their neighbour.

For most countries, the baseline data are the statistics reported by member countries to FAO. We treat all countries recognized in 2010 (or acting like independent countries with regards to fisheries) by the international community as having existed from 1950 to 2010. This is necessary, given our emphasis on ‘places', that is, on time-series of catches taken from specific ecosystems. This also applies to islands and other territories, many of which were colonies, and which have changed status and borders since 1950.

For several countries, the baseline data are provided by international bodies. In the case of EU countries, the baseline data originate from the International Council for the Exploration of the Sea (ICES), which maintains fisheries statistics by smaller statistical areas, as required given the Common Fisheries Policy of the EU. A similar area is the Antarctic waters and surrounding islands, whose fisheries are managed by the Commission for the Conservation of Antarctic Marine Living Resources (CCAMLR), where catch data are available by relatively small statistical areas^50^.

When FAO data are used, care is taken to maintain their assignment to different FAO statistical areas for each country (Supplementary Fig. 1), as they often distinguish between strongly different ecosystems. For example, the Caribbean Sea versus the coast of the Eastern Central Pacific in the case of Panama, Costa Rica, Nicaragua, Honduras and Guatemala. For each maritime country, the area covered extends from the coastline to the edge of the EEZ, including any major coastal lagoons connected to the sea, and the mouths of rivers, that is, estuaries. However, freshwaters are excluded.

(2) Identification of sectors (for example, subsistence, recreational), time periods, species, gears and so on, not covered by (1), that is, missing data components. This is conducted via literature searches and consultations with local experts. This step is one where the contribution of local co-authors and experts is crucial. Potentially, all four sectors defined by us can occur in the marine fisheries of a given coastal country, with the distinction between large-scale and small-scale being the most important^25^. For any entity, we check whether catches originating from the four sectors were included in the reported baseline of catch data, notably by examining their taxonomic composition, and any metadata, which were particularly detailed in the early decades of the FAO ‘Yearbooks'^51^.

The absence of a taxon known to be caught in a country or territory from the baseline data (for example, cockles gleaned by women on the shore of an estuary)^26^ can also be used to identify a fishery that has been overlooked in the official data collection scheme, as can the absence of reef fishes in the coastal data of a Pacific Island state^10^. To avoid double counting, tuna and other large pelagic fishes, unless known to be caught by a local small-scale fishery (and thus in the past not likely reported to a Regional Fisheries Management Organization or RFMO), are not included in this reconstruction step (see below under ‘High Seas and other catches of large pelagic fishes').

Finally, if gears are identified in national data, but a gear known to exist in a given country is not included, then it can be assumed that its catch has been missed, as documented for weirs (*hadrah*) in the Persian Gulf^52^.

(3) Sourcing of alternative information sources on missing sectors identified in (2), via literature searches (peer-reviewed and grey) and consultations with local experts. Information sources include social science studies (anthropology, economics and so on), reports, data sets and expert knowledge. The major initial source of information for catch reconstructions is governments' websites and publications (specifically their Department of Fisheries or equivalent agency), both online and in hard copies. Contrary to what could be expected, it is often not the agency responsible for fisheries research and initial data collection that supplies the catch statistics to FAO, but other agencies, for example, statistical office or agency. As a result, much of the granularity of the original data (that is, catch by sector, by species or by gear) may be lost even before data are prepared for submission to FAO. Furthermore, the data request form sent by FAO each year to each country does not encourage improvements or changes in taxonomic composition, as the form that requests the most recent year's data contains the country's previous years' data in the same composition as submitted in earlier years. This encourages the pooling of detailed data at the national level into the taxonomic categories inherited through earlier (often decades old) FAO reporting schemes, as was discovered, for example, for Bermuda in the early 2000s (ref. 53). Thus, by getting back to the original data, much of the original granularity can be regained during reconstructions.

Additional sources of information on national catches are international organizations such as FAO, ICES or SPC (Secretariat of the Pacific Community), or a Regional Fisheries Management Organization (RFMO) such as NAFO (Northwest Atlantic Fisheries Organization), or CCAMLR^54^, or current or past regional fisheries development and/or management projects (many of them launched and supported by FAO), such as the Bay of Bengal Large Marine Ecosystem project (BOBLME). All these organizations and projects issue reports and publications describing—sometimes in considerable details—the fisheries of their member countries. Another source of information is the academic literature, now widely accessible through Google Scholar.

A good source of information for the earlier decades (especially the 1950s and 1960s) for countries that were part of former colonial empires (especially British or French) are the colonial archives in London (British Colonial Office) and the ‘Archives Nationales d'Outre-Mer', in Aix-en-Provence, and the publications of ORSTOM (Office de la recherche scientifique et technique d'outre-mer), for former French colonies. A further source of information and data are non-fisheries sources, including household and/or nutritional surveys, which are occasionally used for estimating unreported subsistence catches. Our global network of local collaborators is also crucial in this respect, as they have access to key data sets, publications and local knowledge not available elsewhere, often in languages other than English.

Supplementary Figure 2 shows a plot of the publications used for slightly over 110 reconstructions against their date of publication. Although, recent publications predominate, older publications firmly anchor the 1950s catch estimates of many reconstructions. On average, around 35 unique publications were used per reconstruction (not counting online sources and personal communications).

Potential language bias is taken seriously in the *Sea Around Us*, to ensure that data are collated in languages other than English. Besides team members who read Chinese, others speak Arabic, Danish, Filipino/Tagalog, French, German, Hindi, Japanese, Portuguese, Russian, Spanish, Swedish and Turkish. To deal with other languages, research assistants are hired who speak, for example, Korean or Malay/Indonesian. We also rely on our multilingual network of colleagues and friends throughout the world, for example, for Greek or Thai. While it is true that English has now become the undisputed language of science^55^, other languages are used by billions of people, and assembling knowledge about the fisheries of the world is not possible without the capacity to explore the literature in languages other than English.

(4) Development of data ‘anchor points' in time for each missing data item, and expansion of anchor point data to country-wide catch estimates. ‘Anchor' points are catch estimates usually pertaining to a single year and sector, and often to an area not exactly matching the limits of the EEZ or IFA in question. Thus, an anchor point pertaining to a fraction of the coastline of a given country may need to be expanded to the country as a whole. For expansion, we use fisher or population density, or relative IFA or shelf area as raising factor, as appropriate given the local condition. In all cases, we consider that case studies underlying or providing the anchor point data may had a case-selection bias (for example, representing an exceptionally good area or community for study, compared with other areas in the same country), and thus use raising factors very conservatively.

(5) Interpolation for time periods between data anchor points, either linearly or assumption based for commercial fisheries, and generally via per capita (or per fisher) catch rates for non-commercial sectors. Fisheries are often difficult to govern, as they are social activities involving multiple actors. In particular, fishing effort is often difficult to reduce, at least in the short term. Thus, if anchor points are available for years separated by multi-year intervals, it usually will be more reasonable to assume that the underlying fishing activity continues in the intervening years with no data. We tread this ‘continuity' assumption as a default proposition. Exceptions to such continuity assumptions are major environmental impacts such a hurricanes or tsunamis^56^, or major socio-political disturbances, such as military conflicts or civil wars^57^, which we explicitly consider with regards to the use of raising factors and the structure of time series estimates. In such cases, our reconstructions mark the event through a temporary change (for example, decline) in the catch time series, which is documented in the text of each catch reconstruction. At the very least, this provides pointers for future research on the relationship between fishery catches and natural catastrophes or conflicts. We note that the absence of such signals (such as a reduction in catch for a year or two) in the officially reported catch statistics for countries having experienced a major natural or socio-political disturbance can be a sign that their official catch data may not accurately reflect what occurs on the ground. This contributes to the emergence of ‘poor numbers'^40^. Overall, our reconstructions assume—when no information to the contrary is available—that commercial catches (that is, industrial and artisanal) can be linearly interpolated between anchor points, while non-commercial catches (that is, subsistence and recreational) can generally be interpolated between anchor points using non-linear trends in human population numbers or number of fishers over time (via *per capita* rates).

Radical and rapid effort reductions as a result of an intentional policy decision and implementation do not occur widely. One example we are aware of is the trawl ban of 1980 in Western Indonesia^58^. The ban had little or no impact on official Indonesian fisheries statistics for Western Indonesia, another indication that these statistics may have little to do with the realities on the ground. FAO hints at this being widespread in the Western Central Pacific and the Eastern Indian Ocean (the only FAO areas where reported catches appear to be increasing) when they note that ‘while some countries (i.e., the Russian Federation, India and Malaysia) have reported decreases in some years, marine catches submitted to FAO by Myanmar, Vietnam, Indonesia and China show continuous growth, i.e., in some cases resulting in an astonishing decadal increase (e.g., Myanmar up 121 percent, and Vietnam up 47 percent)'.^42^

(6) Estimation of total catch times series. A reconstruction is completed when the estimated catch time series derived through steps 2–5 are combined and harmonized with the reported catch of step 1. Generally, this results in an increase of the overall catch, but several cases exist where the reconstructed total catch is lower than the reported catch. The best documented case of this is that of mainland China^14^, whose over-reported catches for local waters in the Northwest Pacific are compensated for by under-reported catches taken by Chinese distant water fleets fishing elsewhere. In the 2000s, Chinese distant water fleets operated in the EEZs of over 90 countries, that is, in most parts of the world's oceans^5^. Harmonizing reconstructed catches with the reported baselines goes hand-in-hand with documenting the entire reconstruction procedure. Thus, every reconstruction is documented and published, either in the peer-reviewed scientific literature, or as detailed technical reports in the publicly accessible and indexed Fisheries Centre Research Reports series or the Fisheries Centre Working Paper series, or other regional organization reports (Supplementary Table 5).

Several reconstructions were conducted in the mid- to late 2000s, when official reported data (that is, FAO statistics or national data) were not available to 2010 (refs 15, 59). All these cases are updated to 2010, in line with each country's individual reconstruction approach to estimating missing catch data. Thus, all reconstructions are brought to 2010 to ensure identical time coverage (Supplementary Table 5).

Since these six points were originally proposed, a seventh point has come to the fore that cannot be ignored^10^:

(7) Quantifying the uncertainty associated with each reconstruction. In fisheries research, catch data are rarely associated with a measure of uncertainty, at least not in the form resembling confidence intervals. This may reflect the fact that the issue with catch data is not a lack of precision (that is, whether we could expect to produce similar results upon re-estimation), but about accuracy, that is, attempting to eliminate a systematic bias, a type of error which statistical theory does not really address.

We deal with this issue through a procedure related to ‘pedigrees'^60^ and the approach used by the Intergovernmental Panel on Climate Change to quantify the uncertainty in its assessments^61^. The authors of the reconstructions are asked to attribute a ‘score' expressing their evaluation of the quality of the time series data to each fisheries sector (industrial, artisanal and so on) for each of the three time periods (1950–1969, 1970–1989 and 1990–2010). These ‘scores' are (1) ‘very low', (2) ‘low', (3) ‘high' and (4) ‘very high' (Table 1). There is a deliberate absence of an uninformative ‘medium' score, to avoid the effective ‘non-choice' that this option would represent. Each of these scores is assigned a percentage uncertainty range (Table 1). Thereafter, the overall mean weighted percentage uncertainty (over all countries and sectors) was computed (Fig. 1).

### Foreign catches

We define foreign catches as taken by vessels of a maritime state in the EEZ, or EEZ-equivalent waters of another coastal state. Based on our definition of sectors, all foreign fishing in the waters of another country is deemed to be industrial in nature. As the High Seas legally belong to no one (or to everyone), there can be no ‘foreign' catches in the High Seas. Prior to UNCLOS, and the declaration of EEZs by maritime countries, foreign catches were illegal only if conducted without explicit permission within the territorial waters of such countries (generally 12 nautical miles). Since the declarations of EEZs by the overwhelming majority of maritime countries, foreign catches are considered illegal if conducted within the EEZ but without access being granted by the coastal state. A distinct exception is the EU, whose waters are managed by a ‘Common Fisheries Policy', which implies a multilateral ‘access agreement'.

Access permission can be tacit and based on historic rights (‘observed' access), or more commonly in the form of explicit access agreements and involving compensatory payment for the coastal state. The *Sea Around Us*, building on previous work by FAO^62^, has created a database of such access and agreements, which is used to allocate the catches of distant-water fleets to the waters where they were taken.

This information is then harmonized with the catches reported by FAO for countries fishing outside their country's ‘home' FAO areas, which always identifies this catch as distant-water industrial catch (see below for tuna catches reported to RFMOs).

In line with INTERPOL and others^63^, we define illegal fishing as foreign fishing within the EEZ waters of another country without a permission to access these resources. We do not treat domestic fisheries' violations of ‘fishing regulations' as ‘illegal'. In general, our reconstruction method cannot readily distinguish between legal and illegal foreign fishing, as we do not necessarily know about all access agreements^5^,^6^. Thus, our data only pertain to ‘reported' versus ‘unreported' status, irrespective of legality of foreign fleets in a host country^5^. However, for around two dozen countries (mainly in West Africa) where the number of illegally operating vessels could be inferred, the fleet size can be multiplied by appropriate catch per unit of effort rates, leading to an estimate of illegal catch in these EEZs.

### Industrial catches of large pelagic fishes

*Nominal landings data*. To date, there is no single, publicly available data set presenting industrial landings of tuna and large pelagic fishes for the entire world that is separate from the amalgamated FAO statistics, despite these fisheries being among the most valuable in the world^64^. Here, we first compile nominal industrial landings of tuna and other large pelagic fish caught either in the High Seas or within EEZs by fishing gear, taxon, countries and statistical reporting areas from data published by Regional Fisheries Management Organizations. Second, we use partially spatialized landings data provided by staff of the French ‘Institut de recherche pour le développement' to spatially pre-assign the nominal landings data derived from RFMOs (Supplementary Table 6).

For each ocean, the nominal landings data are spatialized according to reported proportions in the previously spatialized data (Supplementary Table 6). For example, if the nominal data reports France catching 100 tonnes of yellowfin tuna (*Thunnus albacares*) in 1983 using longlines, but the spatial data only present 85 tonnes of yellowfin tuna reported in 1983 by France using longlines in four separate statistical cells, the nominal 100 tonnes for France are split into these four spatial cells according to their reported proportion of catch in the spatial dataset. This matching of the nominal and spatial records is done over a series of successive refinements, with the first being the best-case scenario, in which there are matching records for year, country, gear and taxon. The last refinement is the worst-case scenario, in which there are no matching records except for the year of catch. For example, if Sri Lanka reports 100 tonnes of yellowfin tuna caught in 1983 using longlines, but there are no spatial records for any country catching yellowfin tuna in 1983, the nominal 100 tonnes for Sri Lanka are split into spatial cells according to their reported proportions of total catch of any species and gear in 1983. The end result is a baseline landings database containing all matched and spatialized catch records, which sum to the original nominal catch tonnages.

*Discards*. A review of the literature for each ocean provided limited country- and fleet-specific discard data. Therefore, we average the discard rates across the entire time period and apply these to the region of origin of the fleet (for example, East Asia or Western Europe), rather than the actual country of origin of the fleet. Discards were spatialized in conjunction with nominal landings data.

### Assembly of total catches

Ultimately, the total catch extracted from a given area, such as a given EEZ or EEZ-equivalent waters, or high seas waters within a given FAO area is computed as the sum of three data layers: (1) the reconstructed domestic catches within home EEZs (‘Layer 1' data); (2) the derived catch by foreign fleets (‘Layer 2' data); and (3) the tuna and other large pelagic fishes caught in the High Seas and in EEZs (‘Layer 3' data).

### Documentation of the catch reconstructions

The references and web-links of the contributions documenting the catch reconstructions that went into the re-estimation of the global catch of marine fisheries are documented in Supplementary Table 5. Altogether, 273 EEZs (or EEZ ‘components') were covered in 247 catch reconstructions, which had 103 unique first authors and 279 unique co-authors in over 50 countries.

All data presented here are also deposited in the Dryad Digital Depository (DOI: 10.5061/dryad.4s4t1).

### Analyses

To examine if significant breakpoints exist in the catch data time series of both reconstructed total catches and reported catches that may illustrate a change in trends of catches over time (that is, a change in the slope), we analyse the time series trajectories using segmented regression^21^. For both the reconstructed as well as reported time series, we identify two breakpoints, being 1967 and 1996, respectively (Supplementary Table 2). These breakpoints suggest a change in regression slope, with the second breakpoint suggesting a trend reversal. This was validated by testing for a significant difference-in-slope parameter using the Davies test^65^, which tests for a non-zero difference-in-slope of a segmented regression relationship.

## Additional information

**How to cite this article:** Pauly, D. & Zeller, D. Catch reconstructions reveal that global marine fisheries catches are higher than reported and declining. *Nat. Commun.* 7:10244 doi: 10.1038/ncomms10244 (2016).

## Supplementary Material

Supplementary Information Supplementary Figures 1-2, Supplementary Tables 1-6 and Supplementary Acknowledgements

## Figures and Tables

**Table 1:** Scoring system for deriving uncertainty bands for the quality of time series data of reconstructed catches.

**Score**		_**±**_%*	**Corresponding IPCC criteria**†
4	Very high	10	High agreement and robust evidence
3	High	20	High agreement and medium evidence **or** medium agreement and robust evidence
2	Low	30	High agreement and limited evidence **or** medium agreement and medium evidence **or** low agreement and robust evidence.
1	Very low	50	Less than high agreement and less than robust evidence

^*^Percentage uncertainty derived from Monte-Carlo simulations^66^,^67^.

^†^‘Confidence increases' (and hence percentage ranges are reduced) ‘when there are multiple, consistent independent lines of high-quality evidence'^61^.
