# Supplementary material for: Catch reconstructions reveal that global marine fisheries catches are higher than reported and declining
Source: Nat Commun. 2016 Jan 19;7:10244. doi: 10.1038/ncomms10244 (PMC4735634; doi:10.1038/ncomms10244)
Supplement: Supplementary Information — Supplementary Figures 1-2, Supplementary Tables 1-6 and Supplementary Acknowledgements [file ncomms10244-s1.pdf]

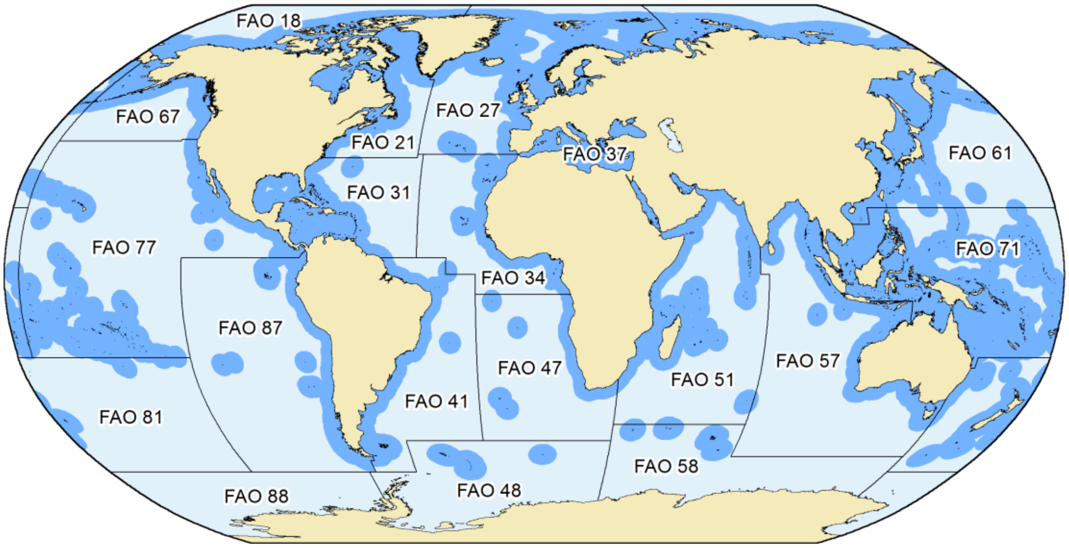

Supplementary Fig. 1. **Spatial categorization of world oceans using Exclusive Economic Zones and FAO statistical areas**. Extent and delimitation of countries' Exclusive Economic Zones (EEZs), as declared by individual countries, or as defined by the *Sea Around Us* based on the fundamental principles outlined in UNCLOS (i.e., 200 nautical miles or mid-line rules), and the FAO statistical areas by which global fisheries catch statistics are reported. Note that for several FAO areas some data exist by sub-areas as provided through regional organizations (e.g., ICES for FAO Area 27). The *Sea Around Us* makes use of these spatially refined data to improve the spatial allocation of catch data.

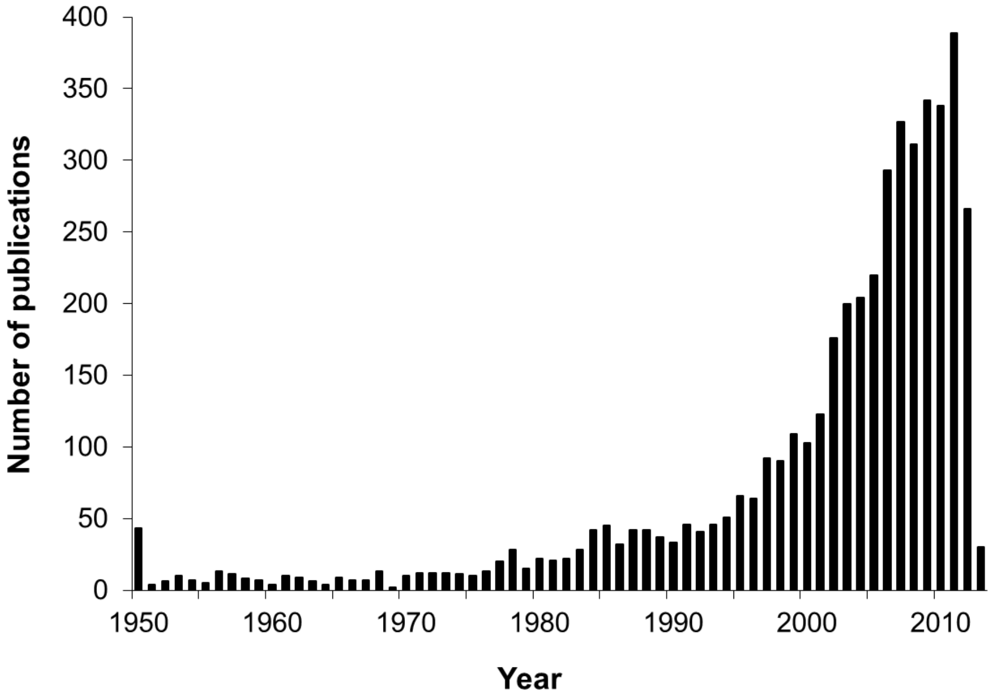

Supplementary Fig. 2. **Year of publication of source material for reconstructions.** Number of publications (scientific and grey literature) and their publication date used for slightly over 110 country/territory catch reconstructions, i.e., less than half. A total of 4,000 publications (excluding personal communications and online sources) were consulted, resulting in an average of 35 publications being used per reconstruction. While more information sources are obviously available for recent time periods, this illustrates that publications are accessible for the entire time period. The slightly elevated number for 1950 is due to pooling of material dated pre-1950 (as far back as the early 20th century or even late 19th century) that was used conservatively to inform 1950 anchor point information.

Supplementary Table 1: Reported and estimated reconstructed total catches (t) for 1950 to 2010, as used for Figure 1. Shown also are the mean weighted percentage uncertainty as derived from the reconstruction data quality scoring.

Year	Reported catch (t)	Reconstructed catch (t)	Lower mean weighted uncertainty (t)	Upper mean weighted uncertainty (t)
1950	17,965,332	30,913,396	14,304,728	47,522,064
1951	19,355,624	32,963,256	15,028,922	50,897,590
1952	20,251,127	34,988,062	16,030,598	53,945,525
1953	21,030,831	35,851,179	16,595,644	55,106,714
1954	23,117,208	38,779,606	17,887,772	59,671,441
1955	23,758,704	39,939,409	18,226,313	61,652,506
1956	25,532,664	42,276,420	19,163,920	65,388,919
1957	25,716,475	42,906,313	19,430,182	66,382,444
1958	26,062,935	43,490,638	19,912,670	67,068,607
1959	28,701,150	47,368,396	21,971,273	72,765,519
1960	31,211,158	51,293,583	24,038,174	78,548,992
1961	35,321,710	56,977,496	27,292,148	86,662,845
1962	38,293,672	60,236,136	29,315,030	91,157,241
1963	39,912,587	63,650,102	31,386,281	95,913,923
1964	44,052,941	69,976,600	35,295,651	104,657,548
1965	43,194,562	70,004,551	35,532,398	104,476,705
1966	46,604,626	74,576,948	38,033,395	111,120,502
1967	49,709,437	80,108,400	41,581,020	118,635,780
1968	53,138,559	86,167,984	45,210,926	127,125,041
1969	51,149,491	81,935,120	42,928,461	120,941,779
1970	57,126,159	88,419,602	46,604,234	130,234,970
1971	56,844,016	87,862,478	46,123,525	129,601,432
1972	52,012,578	81,407,496	41,379,577	121,435,414
1973	51,851,834	81,533,965	41,047,550	122,020,381
1974	55,934,429	85,896,007	44,026,234	127,765,780
1975	54,373,239	85,003,430	43,907,522	126,099,337
1976	57,385,286	88,484,857	46,091,920	130,877,795
1977	57,393,771	88,740,143	44,941,343	132,538,942
1978	60,124,732	92,575,063	47,421,992	137,728,135
1979	58,951,238	90,285,640	46,415,909	134,155,372
1980	59,807,677	90,359,498	46,913,911	133,805,084
1981	61,577,339	93,471,969	48,896,076	138,047,862
1982	65,069,469	100,164,379	52,155,559	148,173,199
1983	63,254,361	98,427,059	51,392,953	145,461,164
1984	68,074,711	105,538,419	56,429,467	154,647,371
1985	70,049,576	109,577,689	58,626,496	160,528,882
1986	75,146,438	115,000,541	61,849,496	168,151,585
1987	76,114,925	117,870,501	62,314,655	173,426,347
1988	79,860,982	121,596,174	65,088,862	178,103,486
1989	81,182,606	123,584,984	65,868,296	181,301,673
1990	78,020,708	119,836,808	63,244,516	176,429,100
1991	76,909,696	118,579,720	62,592,414	174,567,026
1992	78,481,589	120,780,941	64,015,035	177,546,847
1993	79,304,422	120,716,313	64,126,463	177,306,162
1994	84,336,776	126,570,882	68,227,459	184,914,306
1995	84,048,150	126,691,190	66,906,385	186,475,996
1996	85,592,857	130,376,067	69,094,133	191,658,000
1997	85,059,630	127,748,804	67,181,521	188,316,086
1998	77,663,222	117,831,231	59,508,531	176,153,930
1999	83,132,383	122,637,246	64,062,043	181,212,449
2000	84,149,836	124,076,086	65,521,983	182,630,188
2001	81,897,761	120,479,709	62,482,930	178,476,487
2002	83,045,221	119,539,414	61,807,924	177,270,904
2003	80,060,282	116,942,719	59,369,647	174,515,791
2004	84,465,562	121,805,272	63,290,634	180,319,909
2005	83,107,615	119,224,366	61,732,679	176,716,054
2006	79,988,058	114,012,445	57,928,597	170,096,292
2007	80,252,914	114,112,941	58,693,835	169,532,047
2008	79,444,665	111,978,040	57,715,030	166,241,051
2009	79,400,252	111,362,092	57,110,647	165,613,537
2010	76,894,144	108,987,508	54,879,391	163,095,626

Supplementary Table 2. Results of segmented regressions[71] (determines regression breakpoint years and segmented line slopes) and Davies test[72] (testing for non-zero difference-in-slope parameters) for both the global reconstructed and reported fisheries catch time series presented in main text Figure 1.

Catch time series	Segmented regression			Davies test
	Breakpoint (year)	Line	Slope (mt·year^{-1})	
Reconstructed	-	1	2.82	-
	1967	2	1.86	NS
	1996	3	-1.22	< 0.001
Reported	-	1	1.88	-
	1967	2	1.30	NS
	1996	3	-0.38	< 0.001

Supplementary Table 3: Data used for Figure 2. Reconstructed catches with and without discards, and with and without the major countries using quota management (i.e., USA, New Zealand, Australia and Western Europe), as well as seafood caught per capita (with and without discards).

Year	Reconstructed catch (+discards) (t)	Reconstructed catch (-discards)(t)	Reconstructed catch (-discards) excluding quota countries (t)	Reconstructed catch (+discards) excluding quota countries (t)	Seafood (+discards) caught (kg/person)	Seafood (- discards) caught (kg/person)
1950	30,913,396	25,039,226	18,593,113	22,855,919	13.1	11.1
1951	32,963,256	26,685,031	19,916,834	24,252,884	13.6	11.5
1952	34,988,062	27,757,751	20,948,053	26,223,331	14.1	11.7
1953	35,851,179	28,678,242	21,919,206	27,133,849	14.2	11.9
1954	38,779,606	30,766,676	23,394,042	29,313,368	15.0	12.5
1955	39,939,409	31,599,230	24,391,541	30,667,991	15.8	13.1
1956	42,276,420	33,583,868	25,850,631	32,478,920	16.3	13.5
1957	42,906,313	33,907,581	26,341,204	33,285,103	16.5	13.6
1958	43,490,638	34,234,646	26,815,547	34,024,870	16.5	13.7
1959	47,368,396	37,460,393	29,580,696	37,305,929	17.6	14.6
1960	51,293,583	40,397,437	32,793,377	41,388,458	18.8	15.5
1961	56,977,496	45,264,536	37,133,061	46,755,959	20.5	16.9
1962	60,236,136	48,750,490	40,698,076	50,308,215	21.2	17.7
1963	63,650,102	51,317,915	43,250,449	53,550,429	21.8	18.2
1964	69,976,600	57,044,662	48,753,958	59,570,852	23.1	19.3
1965	70,004,551	56,408,859	47,618,245	59,277,757	22.9	19.0
1966	74,576,948	61,221,051	52,075,358	63,518,573	23.7	19.9
1967	80,108,400	66,558,600	57,402,582	69,111,181	24.8	21.0
1968	86,167,984	72,296,966	63,290,252	75,317,531	26.1	22.4
1969	81,935,120	69,173,236	60,404,068	71,269,141	24.3	21.0
1970	88,419,602	75,664,761	66,401,153	77,182,380	25.7	22.4
1971	87,862,478	75,176,673	65,504,786	76,341,658	25.0	21.8
1972	81,407,496	69,049,516	59,002,302	69,104,135	22.8	19.9
1973	81,533,965	69,502,789	59,326,529	69,119,475	22.7	19.9
1974	85,896,007	74,479,573	64,363,341	73,623,203	23.4	20.8
1975	85,003,430	73,690,350	63,164,323	72,263,073	23.0	20.5
1976	88,484,857	77,389,002	65,862,259	74,410,633	23.4	21.0
1977	88,740,143	77,850,414	65,643,255	74,123,691	23.0	20.7
1978	92,575,063	81,702,927	69,691,166	78,137,051	24.0	21.7
1979	90,285,640	79,577,763	67,705,072	75,976,871	22.7	20.5
1980	90,359,498	79,859,266	67,823,711	75,818,606	22.3	20.2
1981	93,471,969	82,487,507	70,429,960	78,934,210	22.7	20.5
1982	100,164,379	87,666,937	75,892,907	85,647,372	23.8	21.4
1983	98,427,059	86,038,585	74,130,347	84,059,093	23.2	20.8
1984	105,538,419	91,611,582	78,897,209	89,904,034	24.1	21.7
1985	109,577,689	94,871,355	82,067,534	93,961,362	24.4	21.8
1986	115,000,541	99,908,899	86,952,522	99,477,724	24.9	22.4
1987	117,870,501	102,200,612	88,750,132	101,552,822	24.8	22.3
1988	121,596,174	105,516,947	91,468,525	104,704,786	24.7	22.3
1989	123,584,984	106,622,257	93,086,670	107,316,459	24.4	21.9
1990	119,836,808	103,137,929	90,425,699	104,559,795	23.1	20.6
1991	118,579,720	102,138,130	89,004,554	102,627,992	22.6	20.2
1992	120,780,941	105,063,072	90,978,258	103,981,363	22.9	20.6
1993	120,716,313	105,597,545	91,313,312	103,737,965	22.5	20.3
1994	126,570,882	112,113,344	97,728,799	109,476,595	23.2	21.1
1995	126,691,190	112,285,168	97,962,868	109,720,102	23.0	20.9
1996	130,376,067	115,686,872	101,787,805	113,967,161	23.4	21.2
1997	127,748,804	113,426,976	98,754,820	110,421,083	22.8	20.7
1998	117,831,231	104,676,970	90,975,666	101,582,567	20.9	19.0
1999	122,637,246	110,211,122	96,956,107	107,131,693	21.5	19.6
2000	124,076,086	112,089,954	98,714,494	108,643,436	21.4	19.6
2001	120,479,709	108,973,257	95,371,294	104,797,034	20.6	18.8
2002	119,539,414	108,747,754	95,270,880	104,149,438	20.2	18.6
2003	116,942,719	106,159,866	93,529,958	102,308,613	19.6	18.0
2004	121,805,272	111,417,333	98,843,465	107,260,851	20.1	18.6
2005	119,224,366	109,209,836	97,388,313	105,606,026	19.5	18.1
2006	114,012,445	104,481,687	93,179,136	100,931,691	18.4	17.1
2007	114,112,941	104,387,449	93,023,038	101,069,938	18.0	16.7
2008	111,978,040	102,409,135	91,745,272	99,691,795	17.6	16.3
2009	111,362,092	101,992,562	91,232,194	98,968,959	17.2	16.0
2010	108,987,508	99,348,620	88,639,690	96,653,238	16.8	15.5

Supplementary Table 4: Data used for Figure 4. Reconstructed catches for all countries in the world, plus High Seas, by large-scale (industrial) and small-scale sectors (artisanal, subsistence, recreational), with discards (overwhelmingly from industrial fisheries) presented separately.

Year	Artisanal (t)	Subsistence (t)	Recreational (t)	Industrial (t)	Discards (t)
1950	7,526,795	2,677,833	268,260	14,566,338	5,874,170
1951	8,278,304	2,704,471	284,319	15,417,937	6,278,225
1952	8,272,109	2,728,141	293,558	16,463,942	7,230,311
1953	8,469,284	2,753,098	292,070	17,163,789	7,172,937
1954	9,226,926	2,895,153	304,398	18,340,199	8,012,930
1955	9,545,554	3,012,167	313,420	18,728,089	8,340,179
1956	10,303,408	3,058,523	319,333	19,902,604	8,692,551
1957	10,425,695	3,112,506	339,291	20,030,089	8,998,732
1958	10,172,920	3,187,292	353,633	20,520,801	9,255,992
1959	10,385,711	3,296,620	355,360	23,422,702	9,908,003
1960	10,703,245	3,479,410	360,842	25,853,941	10,896,146
1961	10,846,177	3,532,629	364,629	30,521,101	11,712,961
1962	11,015,370	3,692,738	383,464	33,658,918	11,485,645
1963	11,005,246	3,826,997	392,172	36,093,501	12,332,187
1964	11,303,832	3,870,677	394,958	41,475,194	12,931,938
1965	11,653,978	3,873,765	405,857	40,475,260	13,595,692
1966	12,084,658	3,877,243	432,286	44,826,863	13,355,898
1967	12,386,026	3,903,718	440,399	49,828,456	13,549,800
1968	12,303,383	3,916,270	460,158	55,617,156	13,871,018
1969	12,211,407	3,889,378	445,673	52,626,777	12,761,884
1970	12,365,128	3,937,638	480,642	58,881,353	12,754,841
1971	12,398,298	3,976,718	508,341	58,293,316	12,685,805
1972	12,753,562	3,983,958	522,985	51,789,011	12,357,980
1973	13,688,372	3,957,258	548,785	51,308,375	12,031,176
1974	13,309,012	3,866,687	583,018	56,720,856	11,416,434
1975	13,432,664	3,808,464	594,247	55,854,975	11,313,080
1976	13,761,132	3,911,536	632,334	59,084,000	11,095,856
1977	13,892,726	4,062,986	653,480	59,241,222	10,889,729
1978	14,360,786	4,029,281	689,377	62,623,483	10,872,136
1979	14,616,134	3,985,743	723,286	60,252,600	10,707,877
1980	14,688,464	3,918,334	762,688	60,489,781	10,500,231
1981	15,279,447	3,986,858	761,247	62,459,956	10,984,462
1982	15,220,824	4,080,200	820,815	67,545,097	12,497,442
1983	15,300,068	4,200,135	849,021	65,689,361	12,388,473
1984	16,074,619	4,138,484	789,467	70,609,012	13,926,837
1985	16,380,243	4,173,602	810,942	73,506,569	14,706,334
1986	16,227,297	4,226,487	848,975	78,606,140	15,091,641
1987	17,449,809	4,216,780	778,434	79,755,589	15,669,889
1988	17,418,253	4,100,400	774,315	83,223,979	16,079,227
1989	17,620,239	4,103,211	763,732	84,135,075	16,962,727
1990	17,734,811	4,075,507	733,903	80,593,708	16,698,879
1991	17,773,521	4,099,117	753,923	79,511,569	16,441,591
1992	18,417,117	4,108,368	744,304	81,793,283	15,717,869
1993	18,031,035	4,072,844	747,610	82,746,056	15,118,768
1994	18,723,265	4,139,764	754,426	88,495,889	14,457,538
1995	19,665,232	4,124,233	752,382	87,743,321	14,406,022
1996	20,787,945	4,081,801	748,967	90,068,159	14,689,195
1997	20,401,848	4,044,958	774,972	88,205,198	14,321,828
1998	20,332,081	4,025,919	744,742	79,574,228	13,154,260
1999	20,588,769	4,057,334	731,511	84,833,508	12,426,124
2000	19,735,866	4,094,731	765,919	87,493,439	11,986,132
2001	20,046,446	3,955,601	769,491	84,201,719	11,506,452
2002	19,742,034	3,879,482	758,863	84,367,375	10,791,660
2003	20,568,530	3,865,552	771,767	80,954,017	10,782,853
2004	21,076,208	3,808,617	771,531	85,760,977	10,387,939
2005	20,966,689	3,718,690	745,431	83,779,026	10,014,530
2006	21,417,851	3,792,749	790,270	78,480,816	9,530,758
2007	21,097,388	3,623,797	804,477	78,861,788	9,725,492
2008	21,111,836	3,629,819	826,843	76,840,638	9,568,906
2009	21,828,623	3,630,156	805,632	75,728,150	9,369,530
2010	21,586,606	3,627,383	808,357	73,326,274	9,638,888

Supplementary Table 5: Sources of reconstructions by country/EEZ component with associated publication links. Publication types are: 1: *Fisheries Centre Working Paper*, 2: *Fisheries Centre Research Reports*, 3: Other reports; 4: Primary literature. Uncertainty scores (as per Table S1) are given below the source of reconstruction for the three time periods 1950-1969, 1970-1989, 1990-2010 in square brackets by fishing sectors: Indust. = industrial, Art. = artisanal, Subs. = subsistence, Recr. = recreational, plus Disc. = discards.

#	Country	Publ. type	Source of reconstruction
1	Albania	1	Moutopoulos, D.K., Bradshaw, B. and Pauly, D. 2015. Reconstruction of Albania fishery catches by fishing gear (1950-2010). Fisheries Centre Working Paper #2015-12, University of British Columbia, Vancouver, 12 p. (http://www.seaaroundus.org/doc/publications/wp/2015/Moutopoulos-et-al-Albania.pdf).<br>[Indust. 2,2,3; Art. 2,2,3; Subs. 1,1,1; Recr. 1,1,1; Disc. 1,1,2]
2	Algeria	2	Belhabib, D., Pauly, D., Harper, S. and Zeller, D. 2012. Reconstruction of marine fisheries catches for Algeria, 1950-2010. pp. 1-22. *In*: D. Belhabib, D. Pauly, S. Harper and D. Zeller (eds.) *Marine fisheries catches in West Africa, 1950-2010, Part I.* Fisheries Centre Research Reports 20(3), University of British Columbia, Vancouver. (http://www.seaaroundus.org/doc/publications/chapters/2012/Belhabib-et-al-Algeria.pdf).<br>[Indust. 3,3,3; Art. 2,2,4; Subs. 3,2,2; Recr. 2,2,2; Disc. 3,3,4]
3	Angola	1,2,4,4	(1) Belhabib, D. and Divovich, E. 2014. Rich fisheries and poor data: a catch reconstruction for Angola, 1950-2010. Fisheries Centre Working Paper #2014-12, University of British Columbia, Vancouver, 19 p. (http://www.seaaroundus.org/doc/publications/wp/2014/Belhabib-et-al-Angola.pdf).<br>(2) Belhabib, D. and Divovich, E. 2015. Rich fisheries and poor data: a catch reconstruction for Angola, 1950-2010, an update of Belhabib and Divovich (2014). pp. 115-128. *In:* D. Belhabib and D. Pauly (eds). *Fisheries catch reconstructions: West Africa, Part II.* Fisheries Centre Research Reports 23(3), University of British Columbia (http://www.seaaroundus.org/doc/publications/chapters/2015/Belhabib-and-Divovich-Angola.pdf).<br>(4) Belhabib, D., Sumaila, U.R., Lam, V.W.Y., Zeller, D., Le Billon, P., Kane, E.A. and Pauly, D. 2015. Euro vs. Yuan: Comparing European and Chinese fishing access in West Africa. *PLoS One* 10(3): e0118351<br>(4) Belhabib, D., Sumaila, U.R. and Pauly, D. 2015. Feeding the poor: contribution of West African fisheries to employment and food security. *Ocean & Coastal Management* 111: 72-81.<br>[Indust. 3,3,2; Art. 1,3,3; Subs. 1,3,3; Recr. 2,3,3; Disc. 2,2,4]
4	Antarctica	4	Ainley, D. and Pauly, D. 2014. Fishing down the food web of the Antarctic continental shelf and slope. *Polar Record,* 50, pp. 92-107.<br>[Indust. -,3,3; Art. -,-,-; Subs. -,-,-; Recr. -,-,-; Disc. -,1,2]
5	Antigua and Barbuda	1	Georges, J., Ramdeen, R., Zylich, K. and Zeller, D. 2015. Reconstruction of total marine fisheries catch for Antigua and Barbuda (1950-2010). Fisheries Centre Working Paper #2015-13, University of British Columbia, Vancouver, 18 p. (http://www.seaaroundus.org/doc/publications/wp/2015/Georges-et-al-Antigua-Barbuda.pdf).<br>[Indust. -,-,-; Art. 2,3,3; Subs. 4,3,3; Recr. 2,2,2; Disc. -,-,-]
6	Argentina	1	Villasante, S., Macho, G., Isusu de Rivero, J., Divovich, E., Zylich, K., Harper, S., Zeller, D. and Pauly, D. 2015. Reconstruction of Argentina's marine fisheries catches (1950-2010). Fisheries Centre Working Paper #2015-50, University of British Columbia, Vancouver, 16 p. (http://www.seaaroundus.org/doc/publications/wp/2015/Villasante-et-al-Argentina.pdf).<br>[Indust. 1,1,2; Art. 1,1,2; Subs. 1,1,2; Recr. 1,1,2; Disc. 1,1,2]
7	Australia	1	Kleisner, K.M., Brennan, C., Garland, A., Lingard, S., Tracey, S., Sahlqvist, P., Tsolos, A., Pauly, D. and Zeller, D. 2015. Australia: reconstructing estimates of total fisheries removals 1950-2010. Fisheries Centre Working Paper #2015-02, University of British Columbia, Vancouver, 26 p. (http://www.seaaroundus.org/doc/publications/wp/2015/Kleisner-et-al-Australia.pdf).<br>[Indust. 2,3,4; Art. 2,3,4; Subs. 1,1,1; Recr. 1,2,3; Disc. 2,2,3]
8	Australia (Christmas Island)	2	Greer, K., Harper, S., Zeller, D. and Pauly, D. 2012. Cocos (Keeling) Islands and Christmas Islands: Brief history of fishing and coastal catches (1950-2010). pp. 1-13. *In*: S. Harper, K. Zylich, L. Boonzaier, F. Le Manach, D. Pauly and D. Zeller (eds.) *Fisheries catch reconstructions: Islands, Part III.* Fisheries Centre Research Reports 20(5), University of British Columbia, Vancouver. (http://www.seaaroundus.org/doc/publications/chapters/2012/Greer-et-al-CocosChristmasIsland.pdf).<br>[Indust. -,-,2; Art. 1,1,1; Subs. 1,1,1; Recr. -,-,-; Disc. -,-,-]
9	Australia (Cocos Keeling Island)	2	Greer, K., Harper, S., Zeller, D. and Pauly, D. 2012. Cocos (Keeling) Islands and Christmas Islands: Brief history of fishing and coastal catches (1950-2010). pp. 1-13. *In*: S. Harper, K. Zylich, L. Boonzaier, F. Le Manach, D. Pauly and D. Zeller (eds.) *Fisheries catch reconstructions: Islands, Part III.* Fisheries Centre Research Reports 20(5), University of British Columbia, Vancouver. (http://www.seaaroundus.org/doc/publications/chapters/2012/Greer-et-al-CocosChristmasIsland.pdf).<br>[Indust. -,-,3; Art. -,1,1; Subs. 1,1,2; Recr. -,1,2; Disc. -,-,-]
10	Australia (Heard	1	Kleisner, K.M., Brennan, C., Garland, A., Lingard, S., Tracey, S., Sahlqvist, P., Tsolos, A., Pauly,

Supplementary Table 5: Sources of reconstructions by country/EEZ component with associated publication links. Publication types are: 1: *Fisheries Centre Working Paper*, 2: *Fisheries Centre Research Reports*, 3: Other reports; 4: Primary literature. Uncertainty scores (as per Table S1) are given below the source of reconstruction for the three time periods 1950-1969, 1970-1989, 1990-2010 in square brackets by fishing sectors: Indust. = industrial, Art. = artisanal, Subs. = subsistence, Recr. = recreational, plus Disc. = discards.

#	Country	Publ. type	Source of reconstruction
	and MacDonald Islands)		D. and Zeller, D. 2015. Australia: reconstructing estimates of total fisheries removals 1950-2010. Fisheries Centre Working Paper #2015-02, University of British Columbia, Vancouver, 26 p. (http://www.seaaroundus.org/doc/publications/wp/2015/Kleisner-et-al-Australia.pdf).<br>[Indust. 2,3,4; Art. 2,3,4; Subs. 1,1,1; Recr. 1,2,3; Disc. 2,2,3]
11	Australia (Lord Howe Island)	1	Kleisner, K.M., Brennan, C., Garland, A., Lingard, S., Tracey, S., Sahlqvist, P., Tsolos, A., Pauly, D. and Zeller, D. 2015. Australia: reconstructing estimates of total fisheries removals 1950-2010. Fisheries Centre Working Paper #2015-02, University of British Columbia, Vancouver, 26 p. (http://www.seaaroundus.org/doc/publications/wp/2015/Kleisner-et-al-Australia.pdf).<br>[Indust. 2,3,4; Art. 2,3,4; Subs. 1,1,1; Recr. 1,2,3; Disc. 2,2,3]
12	Australia (Macquarie Island)	1	Kleisner, K.M., Brennan, C., Garland, A., Lingard, S., Tracey, S., Sahlqvist, P., Tsolos, A., Pauly, D. and Zeller, D. 2015. Australia: reconstructing estimates of total fisheries removals 1950-2010. Fisheries Centre Working Paper #2015-02, University of British Columbia, Vancouver, 26 p. (http://www.seaaroundus.org/doc/publications/wp/2015/Kleisner-et-al-Australia.pdf).<br>[Indust. 2,3,4; Art. 2,3,4; Subs. 1,1,1; Recr. 1,2,3; Disc. 2,2,3]
13	Australia (Norfolk Island)	1	Kleisner, K.M., Brennan, C., Garland, A., Lingard, S., Tracey, S., Sahlqvist, P., Tsolos, A., Pauly, D. and Zeller, D. 2015. Australia: reconstructing estimates of total fisheries removals 1950-2010. Fisheries Centre Working Paper #2015-02, University of British Columbia, Vancouver, 26 p. (http://www.seaaroundus.org/doc/publications/wp/2015/Kleisner-et-al-Australia.pdf).<br>[Indust. 2,3,4; Art. 2,3,4; Subs. 1,1,1; Recr. 1,2,3; Disc. 2,2,3]
14	Bahamas	1	Smith, N.S. and Zeller, D. 2013. Bahamas catch reconstruction: fisheries trends in a tourism-driven economy (1950-2010). Fisheries Centre Working Paper #2013-08, University of British Columbia, Vancouver, 28 p. (http://www.seaaroundus.org/doc/publications/wp/2013/Smith-and-Zeller-Bahamas.pdf).<br>[Indust. 2,4,4; Art. 3,4,4; Subs. 1,1,1; Recr. 1,2,3; Disc. -,-,-]
15	Bahrain	2,4	(2) Al-Abdulrazzak, D. 2013. Missing sectors from Bahrain's reported fisheries catches: 1950-2010. pp. 1-6. *In*: D. Al-Abdulrazzak and D. Pauly (eds.) *From dhows to trawlers: a recent history of fisheries in the Gulf countries, 1950 to 2010.* Fisheries Centre Research Reports 21(2), University of British Columbia, Vancouver. (http://www.seaaroundus.org/doc/publications/chapters/2013/AlAbdulrazzak-et-al-Bahrain.pdf).<br>(4) Al-Abdulrazzak, D., Zeller, D., Belhabib, D., Tesfamichael, D. and Pauly, D. 2015. Total marine fisheries catches in the Persian/Arabian Gulf from 1950-2010. *Regional Studies in Marine Science* 2: 28-34.<br>[Indust. 1,1,1; Art. 3,3,3; Subs. 1,1,1; Recr. 2,2,2; Disc. 2,2,4]
16	Bangladesh	1	Ullah, H., Knip, D., Gibson, D., Zylich, K. and Zeller, D. 2014. Reconstruction of total marine fisheries catches for Bangladesh: 1950-2010. Fisheries Centre Working Paper #2014-15, University of British Columbia, Vancouver, 10 p. (http://www.seaaroundus.org/doc/publications/wp/2014/Ullah-et-al-Bangladesh.pdf).<br>[Indust. 3,2,3; Art. 1,2,2; Subs. 2,2,2; Recr. -,-,-; Disc. 1,2,3]
17	Barbados	1	Mohammed, E., Lindop, A., Parker, C. and Willoughby, S. 2015. Reconstructed fisheries catches of Barbados, 1950-2010. Fisheries Centre Working Paper #2015-16, University of British Columbia, Vancouver, 28 p. (http://www.seaaroundus.org/doc/publications/wp/2015/Mohammed-et-al-Barbados.pdf).<br>[Indust. -,4,4; Art. 2,2,2; Subs. 3,3,3; Recr. 3,3,3; Disc. -,-,-]
18	Belgium	1,4	(1) Lescrauwaet, A.K., Torreele, E., Vincx, M., Polet, H., Mees, J., Lindop, A. and Zylich, K. 2015. Invisible Catch: A century of by-catch and unreported removals in sea fisheries, Belgium 1950-2010. Fisheries Centre Working Paper #2015-18, University of British Columbia, Vancouver, 18 p. (http://www.seaaroundus.org/doc/publications/wp/2015/Lescrauwaet-et-al-Belgium.pdf).<br>(4) Lescrauwaet, A.K., Debergh, H., Vincx, M. and Mees, J. 2010. Fishing in the past: Historical data on sea fisheries landings in Belgium. *Marine Policy* 34(6): 1279-1289<br>[Indust. 3.5,3.5,3.5; Art. 2,-,-; Subs. 1,1,1; Recr. 1,1,1; Disc. 2,2,2]
19	Belize	2	Zeller, D., Graham, R. and Harper, S. 2011. Reconstruction of total marine fisheries catches for Belize, 1950-2008. p. 142-151 *In*: M.L.D. Palomares and D. Pauly (eds.) *Too Precious to Drill: the Marine Biodiversity of Belize*, Fisheries Centre Research Reports 19(6), University of British Columbia, Vancouver. (http://www.seaaroundus.org/doc/publications/chapters/2011/Zeller-et-al-Belize.pdf).<br>Since completing the initial reconstruction, FAO data became available to 2010. To update this reconstruction, the 2008 total reconstructed catch was carried forward to 2010. There were four new categories reported by FAO starting in 2009/2010 (blue shark, longbill spearfish, marlins,

Supplementary Table 5: Sources of reconstructions by country/EEZ component with associated publication links. Publication types are: 1: *Fisheries Centre Working Paper*, 2: *Fisheries Centre Research Reports*, 3: Other reports; 4: Primary literature. Uncertainty scores (as per Table S1) are given below the source of reconstruction for the three time periods 1950-1969, 1970-1989, 1990-2010 in square brackets by fishing sectors: Indust. = industrial, Art. = artisanal, Subs. = subsistence, Recr. = recreational, plus Disc. = discards.

#	Country	Publ. type	Source of reconstruction
			sailfishes, etc. nei, and shortfin mako). These tonnages were all allocated to outside the EEZ. There were also sharp increases starting in 2009/2010 for albacore, atlantic sailfish, bigeye tuna, swordfish and yellowfin tuna. For the tuna and billfish species, FAO tonnages for 2008 were flatlined to 2010, and any additional tonnages were allocated to outside the EEZ for 2009-2010. The ratio and the forward carry were based on the tonnages considered inside the EEZ. The sectoral breakdown of the unreported component was based on the percentage breakdown in 2008. The taxonomic breakdown for both the reported and unreported components was based on the percentage breakdown in 2008 (calculated separately by sector and input).<br>[Indust. 3,3,3; Art. 2,2,2; Subs. 1,1,1; Recr. -,-,-; Disc. 1,2,2]
20	Benin	2,4,4	(2) Belhabib, D. and Pauly, D. 2015. Benin's fisheries: a catch reconstruction, 1950 to 2010. pp. 51-64. *In:* D. Belhabib and D. Pauly (eds). *Fisheries catch reconstructions: West Africa, Part II.* Fisheries Centre Research Reports 23(3), University of British Columbia (http://www.seaaroundus.org/doc/publications/chapters/2015/Belhabib-and-Pauly-Benin.pdf)<br>(4) Belhabib, D., Sumaila, U.R., Lam, V.W.Y., Zeller, D., Le Billon, P., Kane, E.A. and Pauly, D. 2015. Euro vs. Yuan: Comparing European and Chinese fishing access in West Africa. *PLoS One* 10(3): e0118351<br>(4) Belhabib, D., Sumaila, U.R. and Pauly, D. 2015. Feeding the poor: contribution of West African fisheries to employment and food security. *Ocean & Coastal Management* 111: 72-81.<br>[Indust. 3,3,3; Art. 2,4,4; Subs. 3,3,3; Recr. -,-,-; Disc. 2,1,2]
21	Bosnia-Herzegovina	1	Iritani, D., Färber, L., Zylich, K. and Zeller, D. 2015. Reconstruction of fisheries catches for Bosnia-Herzegovina: 1950-2010. Fisheries Centre Working Paper #2015-15, University of British Columbia, Vancouver, 7 p. (http://www.seaaroundus.org/doc/publications/wp/2015/Iritani-et-al-BosniaHerzegovina.pdf).<br>[Indust. -,-,-; Art. 2,2,3; Subs. 4,4,3; Recr. 1,3,3; Disc. -,-,-]
22	Brazil	2	Freire, K.M.F., Aragão, J.A.N., Araújo, A.R.R., Ávila-da-Silva, A.O., Bispo, M.C.S., Canziani, G.V., Carneiro, M.H., Gonçalves, F.D.S., Keunecke, K.A., Mendonça, J.T., Moro, P.S., Motta, F.S., Olavo, G., Pezzuto, P.R., Santana, R.F., Santos, R.A., Trindade-Santos, I., Vasconcelos, J.A., Vianna, M. and Divovich, E. 2015. Reconstruction of catch statistics for Brazilian marine waters (1950-2010). pp. 3-30 *In*: K.M.F. Freire and D. Pauly (eds.), *Fisheries catch reconstructions for Brazil's mainland and oceanic islands.* Fisheries Centre Research Reports 23(4). University of British Columbia, Vancouver. (. http://www.seaaroundus.org/doc/publications/chapters/2015/Freire-et-al-Brazil.pdf)<br>[Indust. 1,3,4; Art. 1,3,4; Subs. 1,2,3; Recr. 1,2,2; Disc. 1,1,1]
23	Brazil (Fernando de Noronha)	2	Divovich, E. and Pauly, D. 2015. Oceanic islands of Brazil: catch reconstruction from 1950 to 2010). pp. 31-48. *In:* K.M.F. Freire and D. Pauly (eds). *Fisheries catch reconstructions for Brazil's mainland and oceanic islands*. Fisheries Centre Research Reports 23(4), University of British Columbia, Vancouver (http://www.seaaroundus.org/doc/publications/chapters/2015/Divovich-and-Pauly-Oceanic-Islands-Brazil.pdf)<br>[Indust. -,-,-; Art. 2,2,2; Subs. 1,1,1; Recr. -,-,-; Disc. 1,1,1]
24	Brazil (St Peter and St Paul Archipelago)	2	Divovich, E. and Pauly, D. 2015. Oceanic islands of Brazil: catch reconstruction from 1950 to 2010). pp. 31-48. *In:* K.M.F. Freire and D. Pauly (eds). *Fisheries catch reconstructions for Brazil's mainland and oceanic islands.* Fisheries Centre Research Reports 23(4), University of British Columbia, Vancouver. . (http://www.seaaroundus.org/doc/publications/chapters/2015/Divovich-and-Pauly-Oceanic-Islands-Brazil.pdf)<br>[Indust. 2,2,2; Art. -,-,-; Subs. -,-,-; Recr. -,-,-; Disc. 1,1,1]
25	Brazil (Trindade & Martim Vaz Islands)	2	Divovich, E. and Pauly, D. 2015. Oceanic islands of Brazil: catch reconstruction from 1950 to 2010). pp. 31-48. *In:* K.M.F. Freire and D. Pauly (eds). *Fisheries catch reconstructions for Brazil's mainland and oceanic islands*. Fisheries Centre Research Reports 23(4), University of British Columbia, Vancouver (http://www.seaaroundus.org/doc/publications/chapters/2015/Divovich-and-Pauly-Oceanic-Islands-Brazil.pdf)<br>[Indust. 2,2,3; Art. 2,2,2; Subs. 1,1,1; Recr. -,-,-; Disc. 1,1,1]
26	Brunei	1	Cinco, E.A., Zylich, K., Teh, L.C.L. and Pauly, D. 2015. The marine and estuarine fisheries of Brunei Darussalam, 1950 to 2010. Fisheries Centre Working Paper #2015-29, University of British Columbia, Vancouver, 16 p. (http://www.seaaroundus.org/doc/publications/wp/2015/Cinco-et-al-Brunei.pdf).<br>[Indust. 3,3.5,3.5; Art. 3,3.5,3.5; Subs. 3,3,3.5; Recr. 2,2.5,2.5; Disc. 2,3,3.5]
27	Bulgaria	1	Keskin, Ç., Ulman, A., Raykov, V., Daskalov, G.M., Zylich, K., Pauly, D. and Zeller, D. 2015. Reconstruction of fisheries catches for Bulgaria: 1950-2010. Fisheries Centre Working Paper #2015-20. University of British Columbia, Vancouver, 18 p.

Supplementary Table 5: Sources of reconstructions by country/EEZ component with associated publication links. Publication types are: 1: *Fisheries Centre Working Paper*, 2: *Fisheries Centre Research Reports*, 3: Other reports; 4: Primary literature. Uncertainty scores (as per Table S1) are given below the source of reconstruction for the three time periods 1950-1969, 1970-1989, 1990-2010 in square brackets by fishing sectors: Indust. = industrial, Art. = artisanal, Subs. = subsistence, Recr. = recreational, plus Disc. = discards.

#	Country	Publ. type	Source of reconstruction
			(http://www.seaaroundus.org/doc/publications/wp/2015/Keskin-et-al-Bulgaria.pdf).<br>[Indust. 2,3,2; Art. 3,3,2; Subs. 1,1,1; Recr. 1,1,1; Disc. 2,2,2]
28	Cambodia	1	Teh, L.C.L., Shon, D., Zylich, K. and Zeller, D. 2014. Reconstructing Cambodia's Marine Fisheries Catch, 1950-2010. Fisheries Centre Working Paper #2014-18, University of British Columbia, Vancouver, 10 p. (http://www.seaaroundus.org/doc/publications/wp/2014/Teh-et-al-Cambodia.pdf).<br>[Indust. -,1,1; Art. 1,2,2; Subs. 1,2,2; Recr. -,-,-; Disc. 2,2,2]
29	Cameroon	2,4,4	(2) Belhabib, D. and Pauly, D. 2015. Reconstructing fisheries catches for Cameroon between 1950-2010. pp. 77-84. *In:* D. Belhabib and D. Pauly (eds*). Fisheries catch reconstructions: West Africa, Part II.* Fisheries Centre Research Reports 23(3), University of British Columbia. . (http://www.seaaroundus.org/doc/publications/chapters/2015/Belhabib-and-Pauly-Cameroon.pdf)<br>(4) Belhabib, D., Sumaila, U.R., Lam, V.W.Y., Zeller, D., Le Billon, P., Kane, E.A. and Pauly, D. 2015. Euro vs. Yuan: Comparing European and Chinese fishing access in West Africa. *PLoS One* 10(3): e0118351<br>(4) Belhabib, D., Sumaila, U.R. and Pauly, D. 2015. Feeding the poor: contribution of West African fisheries to employment and food security. *Ocean & Coastal Management* 111: 72-81.<br>[Indust. 2,2,2; Art. 1,3,3; Subs. 2,2,1; Recr. -,-,-; Disc. 2,2,3]
30	Canada (Arctic)	1,2,4	(1) Teh, L.C.L., Zylich, K. and Zeller, D. 2015. FAO area 18 (Arctic Sea): Catch data reconstruction extension of Zeller *et al*. (2011) to 2010. Fisheries Centre Working Paper #2015-14, University of British Columbia, Vancouver, 5 p. (http://www.seaaroundus.org/doc/publications/wp/2015/Teh-et-al-Arctic-Sea.pdf).<br>(2) Booth, S. and Watts, P. 2007. Canada's Arctic marine fish catches. pp. 3-15. *In:* D. Zeller and D. Pauly (eds.) *Reconstruction of marine fisheries catches for key countries and regions (1950-2005).* Fisheries Centre Research Reports 15(2), University of British Columbia, Vancouver. (http://www.seaaroundus.org/doc/publications/chapters/2007/Booth-and-Watts-Canada-Arctic.pdf).<br>(4) Zeller, D., Booth, S., Pakhomov, E., Swartz, W. and Pauly, D. 2011. Arctic fisheries catches in Russia, USA and Canada: Baselines for neglected ecosystems. *Polar Biology* 34(7): 955-973.<br>[Indust. -,-,-; Art. -,-,-; Subs. 3,3,3; Recr. -,-,-; Disc. -,-,-]
31	Canada (East Coast)	1	Divovich, E., Belhabib, D., Zeller, D. and Pauly, D. 2015. Eastern Canada, "a fishery with no clean hands": Marine fisheries catch reconstruction from 1950 to 2010. Fisheries Centre Working Paper #2015-56, University of British Columbia, Vancouver, 37 p. (http://www.seaaroundus.org/doc/publications/wp/2015/Divovich-et-al-Canada-East.pdf).<br>[Indust. 2,2,1; Art. 2,2,2; Subs. 1,3,1; Recr. 1,2,4; Disc. 4,4,4]
32	Canada (Pacific)	1	Ainsworth, C. 2015. British Columbia Marine Fisheries Catch Reconstruction: 1873 to 2010. Fisheries Centre Working Paper #2015-62, University of British Columbia, Vancouver, 9 p. (http://www.seaaroundus.org/doc/publications/wp/2015/Ainsworth-Canada-BC.pdf).<br>[Indust. 4,4,4; Art. 1,1,2; Subs. 1,1,2; Recr. 3,4,4; Disc. 1,1,1]
33	Cape Verde	2,4,4	(2) Santos, I.T., Monteiro, C.A., Harper, S., Zeller, D. and Belhabib, D. 2012. Reconstruction of marine fisheries catches for the Republic of Cape Verde, 1950-2010. pp. 79-90. *In*: D. Belhabib, D. Zeller, S. Harper and D. Pauly (eds.) *Marine fisheries catches in West Africa, Part I*. Fisheries Centre Research Reports 20(3), University of British Columbia, Vancouver. (http://www.seaaroundus.org/doc/publications/chapters/2012/Santos-et-al-CapeVerde.pdf).<br>(4) Belhabib, D., Sumaila, U.R., Lam, V.W.Y., Zeller, D., Le Billon, P., Kane, E.A. and Pauly, D. 2015. Euro vs. Yuan: Comparing European and Chinese fishing access in West Africa. *PLoS One* 10(3): e0118351<br>(4) Belhabib, D., Sumaila, U.R. and Pauly, D. 2015. Feeding the poor: contribution of West African fisheries to employment and food security. *Ocean & Coastal Management* 111: 72-81.<br>[Indust. 1,1,4; Art. 4,4,4; Subs. 2,2,2; Recr. 2,2,4; Disc. 2,2,2]
34	Chile	1	van der Meer, L., Arancibia, H., Zylich, K. and Zeller, D. 2015. Reconstruction of total marine fisheries catches for mainland Chile (1950-2010). Fisheries Centre Working Paper #2015-91, University of British Columbia, Vancouver, 15 p. (http://www.seaaroundus.org/doc/publications/wp/2015/VanderMeer-et-al-Chile-Mainland.pdf).<br>[Indust. 2,2,4; Art. 2,2,3; Subs. 2,2,3; Recr. 2,2,1; Disc. 1,1,1]
35	Chile (Easter Island)	4,4	(4) Zylich, K., Harper, S., Licandeo, R., Vega, R., Zeller, D. and Pauly, D. 2014. Fishing in Easter Island: a recent history (1950-2010). *Latin American Journal of Aquatic Research* 24(4): 845-856.<br>(4) Zeller, D., Harper, S., Zylich, K. and Pauly, D. 2015. Synthesis of under-reported small-scale fisheries catch in Pacific island waters. *Coral Reefs* 34(1): 25-39.<br>[Indust. 1.3,2.3,3; Art. 1.3,2.3,3; Subs. 1.3,2,2; Disc. -,-,-]

Supplementary Table 5: Sources of reconstructions by country/EEZ component with associated publication links. Publication types are: 1: *Fisheries Centre Working Paper*, 2: *Fisheries Centre Research Reports*, 3: Other reports; 4: Primary literature. Uncertainty scores (as per Table S1) are given below the source of reconstruction for the three time periods 1950-1969, 1970-1989, 1990-2010 in square brackets by fishing sectors: Indust. = industrial, Art. = artisanal, Subs. = subsistence, Recr. = recreational, plus Disc. = discards.

#	Country	Publ. type	Source of reconstruction
36	Chile (Juan Fernandez and Desventuradas Islands)	1	van der Meer, L. and Zylich, K. 2015. Reconstruction of total marine fisheries catches for Juan Fernández Islands and the Desventuradas Islands (1950-2010). Fisheries Centre Working Paper #2015-92, University of British Columbia, Vancouver, 14 p. (http://www.seaaroundus.org/doc/publications/wp/2015/VanderMeer-and-Zylich-Chile-Islands.pdf).<br>[Indust. 2,2,4; Art. 4,4,4; Subs. 2,2,2; Recr. 2,2,1; Disc. 1,1,1]
37	China	1,4	(1) Pauly, D. and Le Manach, F. 2015. Tentative adjustments of China's marine fisheries catches (1950-2010). Fisheries Centre Working Paper #2015-28, University of British Columbia, Vancouver, 16 p. (http://www.seaaroundus.org/doc/publications/wp/2015/Pauly-and-LeMenach-China.pdf).<br>(4) Pauly, D., Belhabib, D., Blomeyer, R., Cheung, W.W.L., Cisneros-Montemayor, A.M., Copeland, D., Harper, S., Lam, V.W.Y., Mai, Y., Le Manach, F., Österblom, H., Mok, K.M., Van der Meer, L., Sanz, A., Antonio, S., Shon, S., Sumaila, U.R., Swartz, W., Watson, R., Zhai, Y. and Zeller, D. 2014. China's distant-water fisheries in the 21st century. *Fish and Fisheries* 15: 474-488.<br>[Indust. 2,3,3; Art. 1,2,2; Subs. 2,2,2; Recr. 1,1,1; Disc. 2,2,2]
38	China (Hong Kong)	1	Cheung, W.W.L. 2015. Reconstructed catches in waters administrated by the Hong Kong Special Administrative Region. Fisheries Centre Working Paper #2015-93, University of British Columbia, Vancouver, 15 p. (http://www.seaaroundus.org/doc/publications/wp/2015/Cheung-Hong-Kong.pdf).<br>[Indust. 3,2,2; Art. 2,3,3; Subs. 2,2,2; Recr. 1,1,1; Disc. 1,1,1]
39	Columbia (Caribbean)	1	Lindop, A., Chen, T., Zylich, K. and Zeller, D. 2015. A reconstruction of Colombia's marine fisheries catches. Fisheries Centre Working Paper #2015-32, University of British Columbia, Vancouver, 15 p. (http://www.seaaroundus.org/doc/publications/wp/2015/Lindop-et-al-Colombia.pdf).<br>[Indust. 2,2,3; Art. 2,2,2; Subs. 2,2,2; Recr. -,-,-; Disc. 2,2,2]
40	Colombia (Pacific)	1	Lindop, A., Chen, T., Zylich, K. and Zeller, D. 2015. A reconstruction of Colombia's marine fisheries catches. Fisheries Centre Working Paper #2015-32, University of British Columbia, Vancouver, 15 p. (http://www.seaaroundus.org/doc/publications/wp/2015/Lindop-et-al-Colombia.pdf).<br>[Indust. 2,2,3; Art. 2,2,2; Subs. 2,2,2; Recr. -,-,-; Disc. 2,2,2]
41	Cook Islands	2,4	Haas, A., Rongo, T., Hefferman, N., Harper, S. and Zeller, D. 2012. Reconstruction of the Cook Islands fisheries catches: 1950-2010, pp. 15-24. *In:* S. Harper, K. Zylich, L. Boonzaier, F. Le Manach, D. Pauly and D. Zeller (eds.) *Fisheries catch reconstructions: Islands, Part III*. Fisheries Centre Research Reports 20(5), University of British Columbia, Vancouver. (http://www.seaaroundus.org/doc/publications/chapters/2012/Haas-et-al-2012-Cook-Islands.pdf).<br>(4) Zeller, D., Harper, S., Zylich, K. and Pauly, D. 2015. Synthesis of under-reported small-scale fisheries catch in Pacific island waters. *Coral Reefs* 34(1): 25-39.<br>[Indust. 4,4,2; Art. 2,2,3; Subs. 2,2,2; Recr. -,-,-; Disc. 3,3,3]
42	Comoros	2	Doherty, B., Hauzer, M. and Le Manach, F. 2015. Reconstructing Catches for the Union of the Comoros: Uniting Historical Sources of Catch Data for Ngazidja, Ndzuwani and Mwali from 1950–2010. pp. 1-11. *In:* F. Le Manach and D. Pauly (eds.) *Fisheries catch reconstructions in the Western Indian Ocean, 1950-2010*. Fisheries Centre Research Report 23(2), University of British Columbia, Vancouver. (http://www.seaaroundus.org/doc/publications/chapters/2015/Doherty-et-al-2015-Comoros.pdf).<br>[Indust. -,-,-; Art. 1,1,1; Subs. 1,1,1; Recr. -,-,-; Disc. -,-,-]
43	Congo (Brazzaville)	2,4,4	(2) Belhabib, D. and Pauly, D. 2015. The implications of misreporting on catch trends: a catch reconstruction for the People's Republic of the Congo, 1950-2010. pp. 95-106. *In*: D. Belhabib and D. Pauly (eds). *Fisheries catch reconstructions: West Africa, Part II.* Fisheries Centre Research Reports 23(3), University of British Columbia(http://www.seaaroundus.org/doc/publications/chapters/2015/Belhabib-and-Pauly-Congo-Brazzaville.pdf)<br>(4) Belhabib, D., Sumaila, U.R., Lam, V.W.Y., Zeller, D., Le Billon, P., Kane, E.A. and Pauly, D. 2015. Euro vs. Yuan: Comparing European and Chinese fishing access in West Africa. *PLoS One* 10(3): e0118351<br>(4) Belhabib, D., Sumaila, U.R. and Pauly, D. 2015. Feeding the poor: contribution of West African fisheries to employment and food security. *Ocean & Coastal Management* 111: 72-81.<br>[Indust. 2,4,3; Art. 2,4,4; Subs. 2,4,4; Recr. -,-,-; Disc. 2,2,2]
44	Congo (ex-Zaïre)	2,4,4	(2) Belhabib, D., Ramdeen, S. and Pauly, D. 2015 An attempt at reconstructing the marine fisheries catches in the Congo (Ex-Zaïre), 1950-2010. pp. 107-114. *In:* D. Belhabib and D. Pauly (eds). *Fisheries catch reconstructions: West Africa, Part II*. Fisheries Centre Research Reports 23(3), University of British Columbia.

Supplementary Table 5: Sources of reconstructions by country/EEZ component with associated publication links. Publication types are: 1: *Fisheries Centre Working Paper*, 2: *Fisheries Centre Research Reports*, 3: Other reports; 4: Primary literature. Uncertainty scores (as per Table S1) are given below the source of reconstruction for the three time periods 1950-1969, 1970-1989, 1990-2010 in square brackets by fishing sectors: Indust. = industrial, Art. = artisanal, Subs. = subsistence, Recr. = recreational, plus Disc. = discards.

#	Country	Publ. type	Source of reconstruction
			(http://www.seaaroundus.org/doc/publications/chapters/2015/Belhabib-and-Pauly-Congo-Ex-Zaiire.pdf)<br>(4) Belhabib, D., Sumaila, U.R., Lam, V.W.Y., Zeller, D., Le Billon, P., Kane, E.A. and Pauly, D. 2015. Euro vs. Yuan: Comparing European and Chinese fishing access in West Africa. *PLoS One* 10(3): e0118351<br>(4) Belhabib, D., Sumaila, U.R. and Pauly, D. 2015. Feeding the poor: contribution of West African fisheries to employment and food security. *Ocean & Coastal Management* 111: 72-81.<br>[Indust. 2,3,1; Art. 2,3,1; Subs. 1,2,2; Recr. -,-,-; Disc. 1,1,1]
45	Costa Rica (Caribbean)	1	Trujillo, P., Cisneros-Montemayor, A., Harper, S., Zylich, K. and Zeller, D. 2015. Reconstruction of Costa Rica's marine fisheries catches, 1950-2010. Fisheries Centre Working Paper #2015-31, University of British Columbia, Vancouver, 16 p. (http://www.seaaroundus.org/doc/publications/wp/2015/Trujillo-et-al-Costa-Rica.pdf).<br>[Indust. 2,3,4; Art. 2,3,4; Subs. 2,3,3; Recr. 1,1,1; Disc. 2,3,4]
46	Costa Rica (Pacific)	1	Trujillo, P., Cisneros-Montemayor, A., Harper, S., Zylich, K. and Zeller, D. 2015. Reconstruction of Costa Rica's marine fisheries catches, 1950-2010. Fisheries Centre Working Paper #2015-31, University of British Columbia, Vancouver, 16 p. (http://www.seaaroundus.org/doc/publications/wp/2015/Trujillo-et-al-Costa-Rica.pdf).<br>[Indust. 2,3,4; Art. 2,3,4; Subs. 2,3,3; Recr. 1,1,1; Disc. 2,3,4]
47	Côte d'Ivoire	2,4,4	(2) Belhabib, D. and Pauly, D. 2015. Côte d'Ivoire: Fisheries catch reconstruction, 1950-2010. pp. 17-36. *In:* D. Belhabib and D. Pauly (eds). *Fisheries catch reconstructions: West Africa, Part II.* Fisheries Centre Research Reports 23(3), University of British Columbia (http://www.seaaroundus.org/doc/publications/chapters/2015/Belhabib-and-Pauly-Cote-d-Ivoirey.pdf)<br>(4) Belhabib, D., Sumaila, U.R., Lam, V.W.Y., Zeller, D., Le Billon, P., Kane, E.A. and Pauly, D. 2015. Euro vs. Yuan: Comparing European and Chinese fishing access in West Africa. *PLoS One* 10(3): e0118351<br>(4) Belhabib, D., Sumaila, U.R. and Pauly, D. 2015. Feeding the poor: contribution of West African fisheries to employment and food security. *Ocean & Coastal Management* 111: 72-81.<br>[Indust. 4,4,4; Art. 2,3,3; Subs. 2,3,2; Recr. -,-,-; Disc. 3,3,2]
48	Croatia	1	Matić-Skoko, S., Soldo, A., Stagličić, N., Blažević, D., Šiljić, J. and Iritani, D. 2014. Croatian Marine Fisheries (Adriatic Sea): 1950-2010. Fisheries Centre Working Paper #2014-26, University of British Columbia, Vancouver, 16 p. (http://www.seaaroundus.org/doc/publications/wp/2014/Matic-Skoko-et-al-Croatia.pdf).<br>[Indust. 2,3,3; Art. 2,3,3; Subs. 1,2,2; Recr. 1,1,1; Disc. 2,2,2]
49	Cuba	2	Au, A., Zylich, K. and Zeller, D. 2014. Reconstruction of total marine fisheries catches for Cuba (1950-2009). pp. 25-32. *In:* K. Zylich, D. Zeller, M. Ang, and D. Pauly (eds.) *Fisheries catch reconstructions: Islands, Part IV*. Fisheries Centre Research Reports 22(2). University of British Columbia, Vancouver. (http://www.seaaroundus.org/about/wp-content/uploads/2014/11/Au-et-al-Cuba-.pdf).<br>[Indust. 2,3,3; Art. 2,3,3; Subs. 1,2,2; Recr. 1,1,1; Disc. 2,2,2]
50	Cyprus (North)	1,4	(1) Ulman, A., Çiçek, B., Salihoglu, I., Petrou, A., Patsalidou, M., Pauly, D. and Zeller, D. 2013. The reconstructed and unified marine fisheries catches of Cyprus, 1950-2010. Fisheries Centre Working Paper #2013 - 09, Fisheries Centre, University of British Columbia, Vancouver. 69 p. (http://www.seaaroundus.org/doc/publications/wp/2013/Ulman-et-al-Cyprus.pdf).<br>(4) Ulman, A., Çiçek, B., Salihoglu, I., Petrou, A., Patsalidou, M., Pauly, D. and Zeller, D. 2014. Unifying the catch data of a divided island: Cyprus's marine fisheries catches, 1950-2010. *Environment, Development and Sustainability*, 21 p. doi: 10.1007/s10668-014-9576-z<br>[Indust. 1.5,-,2.5; Art. 2,2,4; Subs. 1,1,1; Recr. 2,1,3; Disc. -,-,-]
51	Cyprus (South)	1,4	(1) Ulman, A., Çiçek, B., Salihoglu, I., Petrou, A., Patsalidou, M., Pauly, D. and Zeller, D. 2013. The reconstructed and unified marine fisheries catches of Cyprus, 1950-2010. Fisheries Centre Working Paper #2013 - 09, Fisheries Centre, University of British Columbia, Vancouver, 69 p. (http://www.seaaroundus.org/doc/publications/wp/2013/Ulman-et-al-Cyprus.pdf).<br>(4) Ulman, A., Çiçek, B., Salihoglu, I., Petrou, A., Patsalidou, M., Pauly, D. and Zeller, D. 2014. Unifying the catch data of a divided island: Cyprus's marine fisheries catches, 1950-2010. *Environment, Development and Sustainability*, 21 p. doi: 10.1007/s10668-014-9576-z<br>[Indust. 1.5,1.5,2; Art. 2,2,2; Subs. 1,1,1; Recr. 2,1,2; Disc. -,-,-]
52	Denmark (Baltic Sea)	2,4	(2) Bale, S., Rossing, P., Booth, S. and Zeller, D. 2010. Denmark's marine fisheries catches in the Baltic Sea (1950-2007), pp. 39-62. *In:* R. Rossing, S. Booth and D. Zeller (eds.) *Total marine fisheries extractions by country in the Baltic Sea: 1950-present.* Fisheries Centre Research Reports 18(1), University of British Columbia, Vancouver. (http://www.seaaroundus.org/doc/publications/chapters/2010/Bale-et-al-Denmark-Baltic.pdf).

Supplementary Table 5: Sources of reconstructions by country/EEZ component with associated publication links. Publication types are: 1: *Fisheries Centre Working Paper*, 2: *Fisheries Centre Research Reports*, 3: Other reports; 4: Primary literature. Uncertainty scores (as per Table S1) are given below the source of reconstruction for the three time periods 1950-1969, 1970-1989, 1990-2010 in square brackets by fishing sectors: Indust. = industrial, Art. = artisanal, Subs. = subsistence, Recr. = recreational, plus Disc. = discards.

#	Country	Publ. type	Source of reconstruction
			(4) Zeller, D., Rossing, P., Harper, S., Persson, L., Booth, S. and Pauly, D. 2011. The Baltic Sea: estimates of total fisheries removals 1950-2007. *Fisheries Research* 108: 356-363.<br>Since completing the initial reconstruction, ICES landing statistics became available to 2010. To update the reconstruction, ICES landing statistics for 2008-2010 were accepted as the reported landings. The unreported component was calculated using the 2007 IUU rates (by species), which were applied to the reported landings. To calculate discards, the 2007 discard rates (by species) were applied to the sum of reported landings and unreported catches. To calculate recreational catch, population data was first retrieved from Populstat (www.populstat.info), and if needed, a linear interpolation was used to estimate annual population. The 2007 per capita catch rate for the recreational sector was then applied to the 2008-2010 population estimates to calculate total recreational catch for those years. Note that the values and comparisons for the years 1950-2007 were based on the 2007 ICES dataset, and changes were not made to account for small differences within the 2010 dataset regarding previous years.<br>[Indust. 3,3,3; Art. 2,2,2; Subs. 1,1,1; Recr. 2,2,2; Disc. 2,2,2]
53	Denmark (North Sea)	1	Gibson, D., Ueberschaer, B., Zylich, K. and Zeller, D. 2015. Preliminary reconstruction of total marine fisheries catches for Denmark in the Kattegat, the Skagerrak and the North Sea (1950-2010). Fisheries Centre Working Paper #2014-25, University of British Columbia, Vancouver, 12 p. (http://www.seaaroundus.org/doc/publications/wp/2014/Gibson-et-al-Denmark.pdf).<br>[Indust. 3,3,4; Art. 3,3,4; Subs. 1,1,1; Recr. 1,1,2; Disc. 1,1,2]
54	Djibouti	2	Colléter, M., Djibril, A.D., Hosch, G., Labrosse, P., Yvergniaux, Y., Le Manach, F. and Pauly, D. 2015. Le Développement Soutenu de Pêcheries Artisanales: Reconstruction des Captures Marines à Djibouti de 1950 à 2010. pp. 13-25. *In:* F. Le Manach and D. Pauly (eds.) *Fisheries catch reconstructions in the Western Indian Ocean, 1950-2010.* Fisheries Centre Research Report 23(2), University of British Columbia, Vancouver. (http://www.seaaroundus.org/doc/publications/chapters/2015/Colleter-et-al-2015-Djibouti.pdf).<br>[Indust. 4,4,4; Art. 2,3,3; Subs. 2,2,2; Recr. 1,1,2; Disc. 2,2,2]
55	Dominica	2	Ramdeen, R., Harper, S. and Zeller, D. 2014. Reconstruction of total marine fisheries catches for Dominica (1950-2010). pp. 33-42. *In:* K. Zylich, D. Zeller, M. Ang, and D. Pauly (eds.) *Fisheries catch reconstructions: Islands, Part IV.* Fisheries Centre Research Reports 22(2), University of British Columbia, Vancouver. (http://www.seaaroundus.org/doc/publications/chapters/2014/Ramdeen-et-al-Dominica.pdf).<br>[Indust. 2,3,3; Art. 2,2,2; Subs. 1,2,2; Recr. 1,1,1; Disc. 2,2,2]
56	Dominican Republic	2	Van der Meer, L., Ramdeen, R., Zylich, K. and Zeller, D. 2014. Reconstruction of total marine fisheries catches for the Dominican Republic (1950-2009). pp. 43-54. *In:* K. Zylich, D. Zeller, M. Ang, and D. Pauly (eds.) *Fisheries catch reconstructions: Islands, Part IV*. Fisheries Centre Research Reports 22(2), University of British Columbia, Vancouver. (http://www.seaaroundus.org/doc/publications/chapters/2014/VanderMeer-et-al-DominicanRepublic.pdf).<br>[Indust. 2,2,3; Art. 2,4,4; Subs. 2,2,3; Recr. 2,2,2; Disc. 1,1,1]
57	Ecuador	1	Alava, J.J., Lindop, A. and Jacquet, J. 2015. Marine fisheries catch reconstructions for continental Ecuador: 1950-2010. Fisheries Centre Working Paper #2015-34, University of British Columbia, Vancouver, 25 p. (http://www.seaaroundus.org/doc/publications/wp/2015/Alava-et-al-Ecuador.pdf).<br>[Indust. 2,2,2; Art. 3,3,3; Subs. 1,1,1; Recr. 1,1,1; Disc. 1,1,2]
58	Ecuador (Galápagos)	1,4	(1) Schiller, L., Alava, J.-J., Grove, J., Reck, G. and Pauly, D. 2013. A reconstruction of fisheries catches for the Galápagos islands, 1950-2010. Fisheries Centre Working Paper #2013-11, University of British Columbia, Vancouver, 38 p. (http://www.seaaroundus.org/doc/publications/wp/2013/Schiller-et-al-Galapagos.pdf).<br>(4) Schiller, L., Alava, J.-J., Grove, J., Reck, G. and Pauly, D. 2014. A reconstruction of fisheries catches for the Galápagos islands, 1950-2010. *Aquatic Conservation: Freshwater and Marine Ecosystems*, doi: 10.1002/aqc.2458.<br>[Indust. 2,3,3; Art. 2,4,4; Subs. 1,1,2; Recr. -,-,-; Disc. -,-,-]
59	Egypt (Mediterranean)	1	Mahmoud, H., Teh, L.C.L., Khalfallah, M. and Pauly, D. 2015. Reconstruction of marine fisheries statistics in the Egyptian Mediterranean Sea, 1950-2010. Fisheries Centre Working Paper #2015-85, University of British Columbia, Vancouver, 16 p. (http://www.seaaroundus.org/doc/publications/wp/2015/Mahmoud-et-al-Egypt-Med.pdf).<br>[Indust. 2,2,2; Art. 2,2,2; Subs. 2,2,2; Recr. -,1,1; Disc. 2,3,3]
60	Egypt (Red Sea)	2	Tesfamichael, D. and Mehanna, S.F. 2012. Reconstructing Red Sea fisheries of Egypt: Heavy investment and fisheries. pp. 23-50. *In*: D. Tesfamichael and D. Pauly (eds.) *Catch reconstruction for the Red Sea large marine ecosystem by countries (1950 – 2010).* Fisheries Centre Research Reports 20(1), University of British Columbia, Vancouver.

Supplementary Table 5: Sources of reconstructions by country/EEZ component with associated publication links. Publication types are: 1: *Fisheries Centre Working Paper*, 2: *Fisheries Centre Research Reports*, 3: Other reports; 4: Primary literature. Uncertainty scores (as per Table S1) are given below the source of reconstruction for the three time periods 1950-1969, 1970-1989, 1990-2010 in square brackets by fishing sectors: Indust. = industrial, Art. = artisanal, Subs. = subsistence, Recr. = recreational, plus Disc. = discards.

#	Country	Publ. type	Source of reconstruction
			(http://www.seaaroundus.org/doc/publications/chapters/2012/Tesfamichael-and-Mehanna-EgyptRedSea.pdf).<br>[Indust. 2,2,2; Art. 2,2,2; Subs. 2,2,2; Recr. -,1,1; Disc. 2,3,3]
61	El Salvador	1	Donadi, R., Au, A., Zylich, K., Harper, S. and Zeller, D. 2014. Reconstruction of marine fisheries in El Salvador, 1950-2010. Fisheries Centre Working Paper #2015-35, University of British Columbia, Vancouver, 22 p. (http://www.seaaroundus.org/doc/publications/wp/2015/Donadi-et-al-El-Salvador.pdf).<br>[Indust. 2,3,4; Art. 2,3,4; Subs. 2,3,1; Recr. 1,1,1; Disc. 2,3,4]
62	Equatorial Guinea	1,4,4	(1) Belhabib, D., Hellebrandt, D., Allison, E.H. and Pauly, D. 2015. Equatorial Guinea: a catch reconstruction (1950-2010). Fisheries Centre Working Paper #2015-71, University of British Columbia, Vancouver, 24 p. (http://www.seaaroundus.org/doc/publications/wp/2015/Belhabib-et-al-Equatorial-Guinea.pdf).<br>(4) Belhabib, D., Sumaila, U.R., Lam, V.W.Y., Zeller, D., Le Billon, P., Kane, E.A. and Pauly, D. 2015. Euro vs. Yuan: Comparing European and Chinese fishing access in West Africa. *PLoS One* 10(3): e0118351<br>(4) Belhabib, D., Sumaila, U.R. and Pauly, D. 2015. Feeding the poor: contribution of West African fisheries to employment and food security. *Ocean & Coastal Management* 111: 72-81.<br>[Indust. 2,3,3; Art. 2,2,4; Subs. 2,2,4; Recr 2,2,3; Disc. 2,2,2]
63	Eritrea	2	Tesfamichael, D. and Mohamud, S. 2012. Reconstructing Red Sea fisheries catches of Eritrea: a case study of the relationship between political stability and fisheries development. pp. 51-70. *In:* D. Tesfamichael and D. Pauly (eds.) *Catch reconstruction for the Red Sea large marine ecosystem by countries (1950-2010).* Fisheries Centre Research Reports 20(1), University of British Columbia, Vancouver. (http://www.seaaroundus.org/doc/publications/chapters/2012/Tesfamichael-and-Mohamud-Eritrea.pdf).<br>[Indust. 2,3,3; Art. 2,3,3; Subs. 2,3,3; Recr. 1,1,1; Disc. 2,2,2]
64	Estonia	2,4	(2) Veitch, L., Booth, S., Harper, S., Rossing, P. and Zeller, D. 2010. Catch reconstruction for Estonia in the Baltic Sea from 1950-2007, pp. 63-84. *In:* R. Rossing, S. Booth and D. Zeller (eds.) *Total marine fisheries extractions by country in the Baltic Sea: 1950-present.* Fisheries Centre Research Reports 18(1), University of British Columbia, Vancouver. (http://www.seaaroundus.org/doc/publications/chapters/2010/Veitch-et-al-Estonia.pdf).<br>(4) Zeller, D., Rossing, P., Harper, S., Persson, L., Booth, S. and Pauly, D. 2011. The Baltic Sea: estimates of total fisheries removals 1950-2007. *Fisheries Research* 108: 356-363.<br>Since completing the initial reconstruction, ICES landing statistics became available to 2010. To update the reconstruction, ICES landing statistics for 2008-2010 were accepted as the reported landings. The unreported component was calculated using the 2007 IUU rates (by species), which were applied to the reported landings. To calculate discards, the 2007 discard rates (by species) were applied to the sum of reported landings and unreported catches. To calculate recreational catch, population data was first retrieved from Populstat (www.populstat.info), and if needed, a linear interpolation was used to estimate annual population. The 2007 per capita catch rate for the recreational sector was then applied to the 2008-2010 population estimates to calculate total recreational catch for those years. Please note that the values and comparisons for the years 1950-2007 were based on the 2007 ICES dataset, and changes were not made to account for small differences within the 2010 dataset regarding previous years.<br>[Indust. 3,3,4; Art. -,-,-; Subs. -,-,-; Recr 3,3,2; Disc. 2,2,2]
65	Faeroe Islands	1	Gibson, D., Zylich, K. and Zeller, D. 2015. Preliminary reconstruction of total marine fisheries catches for the Faeroe Islands in EEZ- equivalent waters (1950-2010). Fisheries Centre Working Paper #2015-36, University of British Columbia, Vancouver, 12 p. (http://www.seaaroundus.org/doc/publications/wp/2015/Gibson-et-al-Faroe-Islands.pdf).<br>[Indust. 2,3,4; Art. 2,3,4; Subs. 1,1,1; Recr 1,1,1; Disc. 1,1,1]
66	Fiji	2,4	(2) Zylich, K., O'Meara, D., Jacquet, J., Harper, S. and Zeller, D. 2012. Reconstruction of marine fisheries catches for the Republic of Fiji (1950-2009). pp. 25-36. *In:* S. Harper, K. Zylich, L. Boonzaier, F. Le Manach, D. Pauly, and D. Zeller (eds.) *Fisheries catch reconstructions: Islands, Part III*. Fisheries Centre Research Reports 20(5), University of British Columbia, Vancouver. (http://www.seaaroundus.org/doc/publications/chapters/2012/Zylich-et-al-2012-Fiji.pdf).<br>(4) Zeller, D., Harper, S., Zylich, K. and Pauly, D. 2015. Synthesis of under-reported small-scale fisheries catch in Pacific island waters. *Coral Reefs* 34(1): 25-39.<br>[Indust. 3,3,4; Art. 3,3,3; Subs. 2,2,2; Recr. 4,4,4; Disc. 2,2,3]
67	Finland	2,4	(2) Rossing, P., Bale, S., Harper, S. and Zeller, D. 2010. Baltic Sea fisheries catches for Finland (1950-2007). pp. 85-106. *In:* R. Rossing, S. Booth and D. Zeller (eds.), *Total marine fisheries extractions by country in the Baltic Sea: 1950-present*. Fisheries Centre Research Reports 18(1), University of British Columbia, Vancouver.

Supplementary Table 5: Sources of reconstructions by country/EEZ component with associated publication links. Publication types are: 1: *Fisheries Centre Working Paper*, 2: *Fisheries Centre Research Reports*, 3: Other reports; 4: Primary literature. Uncertainty scores (as per Table S1) are given below the source of reconstruction for the three time periods 1950-1969, 1970-1989, 1990-2010 in square brackets by fishing sectors: Indust. = industrial, Art. = artisanal, Subs. = subsistence, Recr. = recreational, plus Disc. = discards.

#	Country	Publ. type	Source of reconstruction
			(http://www.seaaroundus.org/doc/publications/chapters/2010/Rossing-et-al-Finland.pdf).<br>(4) Zeller, D., Rossing, P., Harper, S., Persson, L., Booth, S. and Pauly, D. 2011. The Baltic Sea: estimates of total fisheries removals 1950-2007. *Fisheries Research* 108: 356-363.<br>Since completing the initial reconstruction, ICES landing statistics became available to 2010. To update the reconstruction, ICES landing statistics for 2008-2010 were accepted as the reported landings. The unreported component was calculated using the 2007 IUU rates (by species), which were applied to the reported landings. To calculate discards, the 2007 discard rates (by species) were applied to the sum of reported landings and unreported catches. To calculate recreational catch, population data was first retrieved from Populstat (www.populstat.info), and if needed, a linear interpolation was used to estimate annual population. The 2007 per capita catch rate for the recreational sector was then applied to the 2008-2010 population estimates to calculate total recreational catch for those years. Please note that the values and comparisons for the years 1950-2007 were based on the 2007 ICES dataset, and changes were not made to account for small differences within the 2010 dataset regarding previous years.<br>[Indust. 2,3,3; Art. 2,3,3; Subs. 1,1,1; Recr. 2,3,3; Disc. 2,3,3]
68	France (Atlantic Coast)	1	Bultel, E., Gascuel, D., Le Manach, F., Pauly, D. and Zylich, K. 2015. Catch reconstruction for the French Atlantic coasts 1950-2010. Fisheries Centre Working Paper #2015-37, University of British Columbia, Vancouver, 20 p. (http://www.seaaroundus.org/doc/publications/wp/2015/Bultel-et-al-France-Atlantic.pdf).<br>[Indust. 3,3,3; Art. 3,3,3; Subs. 1,1,1; Recr. 2,2,2; Disc. 2,2,2]
69	France (Clipperton Island)	2	Pauly, D. 2009. The fisheries resources of the Clipperton Island EEZ (France), pp. 35-37. *In*: D. Zeller and S. Harper (eds.) *Fisheries catch reconstructions: Islands, Part I.* Fisheries Centre Research Reports 17(5), University of British Columbia, Vancouver. (http://www.seaaroundus.org/doc/publications/chapters/2009/Pauly-Clipperton-France.pdf).<br>No updated needed as domestic catches are nil.<br>[Indust. -,-,-; Art. -,-,-; Subs. -,-,-; Recr. -,-,-; Disc. -,-,-]
70	France (Corsica)	1,2	(1) Le Manach, F. and Pauly, D. 2015. Update of the fisheries catch reconstruction of Corsica (France), 1950-2010. Fisheries Centre Working Paper #2015-33, University of British Columbia, Vancouver, 5 p. (http://www.seaaroundus.org/doc/publications/wp/2015/LeMenach-and-Pauly-Corsica.pdf).<br>(2) Le Manach, F., Dura, D., Pere, A., Riutor, J.J., Lejeune, P., Santoni, M.C., Culioli, J.M. and Pauly, D. 2011. Preliminary estimates of total fisheries catch in Corsica, France (1950-2008) pp. 3-14 *In:* S. Harper and D. Zeller (eds.) *Fisheries catch reconstruction: Islands, Part II*. Fisheries Centre Research Reports 19(4), University of British Columbia, Vancouver. (http://www.seaaroundus.org/doc/publications/chapters/2011/Le-Menach-et-al-Corsica-France.pdf).<br>[Indust. 2.5,3.5,3.5; Art. 2,3,3; Subs. 1,1,1; Recr. 2,2,2; Disc. -,-,-]
71	France (Crozet Islands)	2	Pruvost, P., Duhamel, G., Gasco, N. and Palomares, M.L.D. 2015. A short history of the fisheries of Crozet Islands. *In:* M.L.D. Palomares and D. Pauly (eds.) *Marine Fisheries Catches of Sub- Antarctic Islands, 1950 to 2010.* p. 30-35. Fisheries Centre Research Reports 23(1), University of British Columbia, Vancouver. (http://www.seaaroundus.org/doc/publications/chapters/2015/Pruvost-et-al-2015-Crozet-Islands.pdf).<br>[Indust. 3,3,4; Art. -,-,-; Subs. -,-,-; Recr. -,-,-; Disc. 3,3,4]
72	France (Guadeloupe)	2	Frotté, L., Harper, S., Veitch, L., Booth, S. and Zeller, D. 2009. Reconstruction of marine fisheries catches for Guadeloupe from 1950-2007. pp. 13-19. *In:* D. Zeller and S. Harper (eds.) *Fisheries catch reconstructions: Islands, Part I.* Fisheries Centre Research Reports 17(5), University of British Columbia, Vancouver. (http://www.seaaroundus.org/doc/publications/chapters/2009/Frotte-et-al-Guadeloupe.pdf).<br>To update this reconstruction the 2007 total reconstructed catch was carried forward, unaltered, to 2010. The FAO data constituted the reported portion of the catch. The unreported catch component for 2008-2010 was taken to be the difference between the 2007 total reconstructed catch amount and the FAO totals. The reported component was assigned to the artisanal sector. For the unreported component, the same sectoral breakdown for 2007 was applied to 2008-2010. The taxonomic breakdowns remained the same for all sectors for both the reported and unreported components. The only retroactive change made to the data was the removal of the assumed reported catches of the islands Saint Barthélemy and Saint Martin. The secession of the islands of Saint Barthélemy and Saint Martin from Guadeloupe was finalized in 2007. The 2011 FAO dataset was the first to account for this, with reported data for these islands separated from Guadeloupe in the years 2007-2011. The proportion of reported data allocated to each island from Guadeloupe's FAO data in 2007 was carried back to 1950 in order to extract an assumed reported baseline for the islands. These catches were removed from the Guadeloupe reconstruction.<br>[Indust. -,-,-; Art. 2,3,2; Subs. 1,1,1; Recr 1,1,2; Disc. -,-,-]

Supplementary Table 5: Sources of reconstructions by country/EEZ component with associated publication links. Publication types are: 1: *Fisheries Centre Working Paper*, 2: *Fisheries Centre Research Reports*, 3: Other reports; 4: Primary literature. Uncertainty scores (as per Table S1) are given below the source of reconstruction for the three time periods 1950-1969, 1970-1989, 1990-2010 in square brackets by fishing sectors: Indust. = industrial, Art. = artisanal, Subs. = subsistence, Recr. = recreational, plus Disc. = discards.

#	Country	Publ. type	Source of reconstruction
73	France (French Guiana)	1	Harper, S., Frotté, L., Booth, S., Veitch, L. and Zeller, D. 2015. Reconstruction of marine fisheries catches for French Guiana from 1950-2010. Fisheries Centre Working Paper #2015-07, University of British Columbia, Vancouver, 11 p. (http://www.seaaroundus.org/doc/publications/wp/2015/Harper-et-al-Guiana.pdf).<br>[Indust. 2,3,3; Art. 2,3,2; Subs. 1,1,1; Recr -,-,-; Disc. 2,2,3]
74	France (Kerguelen Islands)	2	Palomares, M.L.D. and Pauly, D. 2011. A brief history of fishing in the Kerguelen Islands, France, pp. 15-20. *In:* S. Harper and D. Zeller (eds.) *Fisheries catch reconstructions: Islands, Part II*. Fisheries Centre Research Reports 19(4), University of British Columbia, Vancouver. (http://www.seaaroundus.org/doc/publications/chapters/2011/Palomares-and-Pauly-KerguelenIs.pdf).<br>[Indust. 3,3,4; Art. -,-,-; Subs. -,-,-; Recr. -,-,-; Disc. 3,3,4]
75	France (Martinique)	2	Frotté, L., Harper, S., Veitch, L., Booth, S. and Zeller, D. 2009. Reconstruction of marine fisheries catches for Martinique, 1950-2007. pp. 21-26. *In:* D. Zeller and S. Harper (eds.) *Fisheries catch reconstructions: Islands, Part I.* Fisheries Centre Research Reports 17(5), University of British Columbia, Vancouver. (http://www.seaaroundus.org/doc/publications/chapters/2009/Frotte-et-al-Martinique.pdf).<br>To update this reconstruction the total reconstructed catch was carried forward to 2010. The FAO data constituted the reported portion of the catch. The ratio between the FAO reported component and total reconstructed component for 2007 was calculated and applied to the FAO data for 2008-2010 to estimate the total reconstructed amounts for those years. The unreported component for 2008-2010 was then taken to be the difference between those two numbers each year. The reported component was assigned to the artisanal sector. For the unreported component, the same sectoral breakdown for 2007 was applied to 2008-2010. The taxonomic breakdowns remained the same for all sectors for both the reported and unreported components. Some other changes that were made to the data included: (1) correction of the subsistence portion (it was missing taxon groups from older workings) and (2) adding in the Clams, etc. nei group from the FAO data. Please note that the values and comparisons for the years 1950-2007 were based on the 2007 FAO dataset, and changes were not made to account for small differences within the 2010 dataset regarding previous years.<br>[Indust. -,-,-; Art. 2,3,2; Subs. 1,2,1; Recr. 1,1,2; Disc. -,-,-]
76	France (Mayotte)	2	Doherty, B., Herfaut, J., Le Manach, F., Harper, S. and Zeller, D. 2015. Reconstructing Domestic Marine Fisheries in Mayotte from 1950–2010. pp. 53-65. *In:* F. Le Manach and D. Pauly (eds.) *Fisheries catch reconstructions in the Western Indian Ocean, 1950-2010.* Fisheries Centre Research Report 23(2), University of British Columbia, Vancouver. (http://www.seaaroundus.org/doc/publications/chapters/2015/Doherty-et-al-2015-Mayotte.pdf).<br>[Indust. -,-,-; Art. 1,1,3; Subs. 1,1,3; Recr. -,1,1; Disc. -,-,1]
77	France (Mediterranean)	1,4	(1) Bultel, E., Le Manach, F., Ulman, A. and Zeller, D. 2015. Catch reconstruction for the French Mediterranean Sea, 1950-2010. Fisheries Centre Working Paper #2015-38, University of British Columbia, Vancouver, 20 p. (http://www.seaaroundus.org/doc/publications/wp/2015/Bultel-et-al-France-Med.pdf).<br>(4) Pauly, D., Ulman, A., Piroddi, C., Bultel, E. and Coll, M. 2014. 'Reported' versus 'likely' fisheries catches of four Mediterranean countries. pp. 11-17 *In:* J. Lleonart and F. Maynou (eds.) The Ecosystem approach to fisheries in the Mediterranean and Black Seas. *Scientia Marina* 78S1.<br>[Indust. 3,3,3; Art. 3,3,3; Subs. 1,1,1; Recr. 2,2,2; Disc. 2,2,2]
78	France (New Caledonia)	2,4	(2) Harper, S., Frotté, L., Bale, S., Booth, S. and Zeller, D. 2009. Reconstruction of total marine fisheries catches for New Caledonia (1950-2007). pp. 67-75. *In*: S. Harper and D. Zeller (eds.) *Fisheries catch reconstructions: Islands, Part I.* Fisheries Centre Research Reports 17(5), University of British Columbia, Vancouver. (http://www.seaaroundus.org/doc/publications/chapters/2009/Harper-et-al-2009-New-Caledonia.pdf).<br>(4) Zeller, D., Harper, S., Zylich, K. and Pauly, D. 2015. Synthesis of under-reported small-scale fisheries catch in Pacific island waters. *Coral Reefs* 34(1): 25-39.<br>[Indust. 2,3,3; Art. 2,3,3; Subs. 2,2,2; Recr. 1,1,1; Disc. 2,3,3]
79	France (French Polynesia)	2,4	(2) Bale, S., Frotté, L., Harper, S. and Zeller, D. 2009. Reconstruction of total marine fisheries catches for French Polynesia (1950-2007). pp. 53-65. *In:* S. Harper and D. Zeller (eds.) *Fisheries catch reconstructions: Islands, Part I.* Fisheries Centre Research Reports 17(5), University of British Columbia, Vancouver. (http://www.seaaroundus.org/doc/publications/chapters/2009/Bale-et-al-2009-French-Polynesia.pdf).<br>(4) Zeller, D., Harper, S., Zylich, K. and Pauly, D. 2015. Synthesis of under-reported small-scale fisheries catch in Pacific island waters. *Coral Reefs* 34(1): 25-39.<br>[Indust. 3,3,3; Art. 2,2,2; Subs. 1,1,1; Recr. 2,2,2; Disc. 2,2,2]
80	France (Réunion)	2	Le Manach, F., Bach, P., Barret, L., Guyomard, D., Fleury, P.G., Sabarros, P.S. and Pauly, D. 2015.

Supplementary Table 5: Sources of reconstructions by country/EEZ component with associated publication links. Publication types are: 1: *Fisheries Centre Working Paper*, 2: *Fisheries Centre Research Reports*, 3: Other reports; 4: Primary literature. Uncertainty scores (as per Table S1) are given below the source of reconstruction for the three time periods 1950-1969, 1970-1989, 1990-2010 in square brackets by fishing sectors: Indust. = industrial, Art. = artisanal, Subs. = subsistence, Recr. = recreational, plus Disc. = discards.

#	Country	Publ. type	Source of reconstruction
			Reconstruction of the Domestic and Distant-Water Fisheries Catch of La Réunion (France), 1950–2010. pp. 83-98. *In:* F. Le Manach and D. Pauly (eds.) *Fisheries catch reconstructions in the Western Indian Ocean, 1950-2010.* Fisheries Centre Research Report 23(2), University of British Columbia, Vancouver. (http://www.seaaroundus.org/doc/publications/chapters/2015/LeManach-et-al-2015-La-Reunion.pdf).<br>[Indust. 2,3,3; Art. 3,3,3; Subs. 2,2,2; Recr. 1,1,1; Disc. 2,2,2]
81	France (Îles Éparses)	2	Le Manach, F. and Pauly, D. 2015. First Estimate of Unreported Catch in the French Îles Éparses, 1950-2010. pp. 27-35. *In:* F. Le Manach and D. Pauly (eds.) *Fisheries catch reconstructions in the Western Indian Ocean, 1950-2010.* Fisheries Centre Research Report 23(2), University of British Columbia, Vancouver. (http://www.seaaroundus.org/doc/publications/chapters/2015/LeManach-and-Pauly-2015-Iles-Eparses.pdf).<br>[Indust. 1,2,3; Art. 1,1,2; Subs. 1,1,2; Recr. -,-,-; Disc. 1,1,1]
82	France (Saint Barthélemy)	1	Bultel, E., Lindop, A., Ramdeen, R. and Zylich, K. 2015. Reconstruction of marine fisheries catches for St. Barthélémy and St. Martin (French Caribbean, 1950-2010). Fisheries Centre Working Paper #2015-39, University of British Columbia, Vancouver, 9 p. (http://www.seaaroundus.org/doc/publications/wp/2015/Bultel-et-al-St-Barts-St-Martin.pdf).<br>[Indust. 2,3,3; Art. 2,3,3; Subs. 2,2,2; Recr. 1,1,1; Disc. 2,2,2]
83	France (Saint Martin)	1	Bultel, E., Lindop, A., Ramdeen, R. and Zylich, K. 2015. Reconstruction of marine fisheries catches for St. Barthélémy and St. Martin (French Caribbean, 1950-2010). Fisheries Centre Working Paper #2015-39, University of British Columbia, Vancouver, 9 p. (http://www.seaaroundus.org/doc/publications/wp/2015/Bultel-et-al-St-Barts-St-Martin.pdf).<br>[Indust. 2,3,3; Art. 2,3,3; Subs. 2,2,2; Recr. 1,1,1; Disc. 2,2,2]
84	France (St Paul and Amsterdam)	2	Pruvost, P., Duhamel, G., Le Manach, F. and Palomares, M.L.D. 2015. A short history of the fisheries of Saint-Paul and Amsterdam Islands. pp. 36-44. *In:* M.L.D. Palomares and D. Pauly (eds.) *Marine Fisheries Catches of Sub- Antarctic Islands, 1950 to 2010.* Fisheries Centre Research Reports 23(1), University of British Columbia, Vancouver. (http://www.seaaroundus.org/doc/publications/chapters/2015/Pruvost-et-al-2015-StPaul-Amsterdam.pdf).<br>[Indust. 3,3,4; Art. -,-,-; Subs. -,-,-; Recr. -,-,-; Disc. 3,3,4]
85	France (Saint Pierre et Miquelon)	1	Bultel, E. and Zylich, K. 2015. Fisheries catch reconstruction of the Western Atlantic French archipelago of Saint Pierre et Miquelon, 1950-2010. Fisheries Centre Working Paper #2015-42, University of British Columbia, Vancouver, 15 p. http://www.seaaroundus.org/doc/publications/wp/2015/Bultel-and-Zylich-St-Pierre-et-Miquelon.pdf).<br>[Indust. 3,3,4; Art. 3,3,4; Subs. 2,2,2; Recr. 1,1,1; Disc. 2,2,2]
86	France (Wallis and Futuna)	2,4	(2) Harper, S., Frotté, L., Booth, S. and Zeller, D. 2009. Reconstruction of marine fisheries catches for Wallis and Futuna Islands (1950-2007). pp. 99-104. *In*: S. Harper and D. Zeller (eds.) *Fisheries catch reconstructions: Islands, Part I.* Fisheries Centre Research Reports 17(5), University of British Columbia, Vancouver. (http://www.seaaroundus.org/doc/publications/chapters/2009/Harper-et-al-2009-Wallis-and-Futuna-Islands.pdf).<br>(4) Zeller, D., Harper, S., Zylich, K. and Pauly, D. 2015. Synthesis of under-reported small-scale fisheries catch in Pacific island waters. *Coral Reefs* 34(1): 25-39.<br>[Indust. 2,3,3; Art. 3,3,3; Subs. 2,3,3; Recr. 1,1,1; Disc. 2,2,2]
87	Gabon	2,4,4	(2) Belhabib, D. 2015. Gabon fisheries between 1950-2010: a catch reconstruction. pp. 85-94. *In*: D. Belhabib and D. Pauly (eds). *Fisheries catch reconstructions: West Africa, Part II.* Fisheries Centre Research Reports 23(3), University of British Columbia (http://www.seaaroundus.org/doc/publications/chapters/2015/Belhabib-Gabon.pdf) (4) Belhabib, D., Sumaila, U.R., Lam, V.W.Y., Zeller, D., Le Billon, P., Kane, E.A. and Pauly, D. 2015. Euro vs. Yuan: Comparing European and Chinese fishing access in West Africa. *PLoS One* 10(3): e0118351<br>(4) Belhabib, D., Sumaila, U.R. and Pauly, D. 2015. Feeding the poor: contribution of West African fisheries to employment and food security. *Ocean & Coastal Management* 111: 72-81.<br>[Indust. 3,3,4; Art. 2,4,4; Subs. 3,2,3; Recr -,-,-; Disc. 2,2,3]
88	Gambia	1,4,4,4	(1) Belhabib, D., Mendy, A. and Pauly, D. 2013. Big fishing for small fishes: six decades of fisheries in The Gambia, "the smiling coast of Africa's". Fisheries Centre Working Papers #2013-07, University of British Columbia, Vancouver, 20 p. (http://www.seaaroundus.org/doc/publications/wp/2014/Belhabib-et-al-Gambia.pdf).<br>(4) Belhabib, D., Sumaila, U.R., Lam, V.W.Y., Zeller, D., Le Billon, P., Kane, E.A. and Pauly, D. 2015. Euro vs. Yuan: Comparing European and Chinese fishing access in West Africa.

Supplementary Table 5: Sources of reconstructions by country/EEZ component with associated publication links. Publication types are: 1: *Fisheries Centre Working Paper*, 2: *Fisheries Centre Research Reports*, 3: Other reports; 4: Primary literature. Uncertainty scores (as per Table S1) are given below the source of reconstruction for the three time periods 1950-1969, 1970-1989, 1990-2010 in square brackets by fishing sectors: Indust. = industrial, Art. = artisanal, Subs. = subsistence, Recr. = recreational, plus Disc. = discards.

#	Country	Publ. type	Source of reconstruction
			PLoS One 10(3): e0118351<br>(4) Belhabib, D., Sumaila, U.R. and Pauly, D. 2015. Feeding the poor: contribution of West African fisheries to employment and food security. Ocean & Coastal Management 111: 72-81.<br>(4) Belhabib, D., Mendy, A., Subah, Y., Broh, N.T., Jueseah, A.S., Nipey, N., Boeh, W.W., Willemse, N., Zeller, D. and Pauly, D. (in press) Fisheries catch under-reporting in The Gambia, Liberia and Namibia, and the three Large Marine Ecosystems which they represent. *Environmental Development*. DOI: 10.1016/j.envdev.2015.08.004<br>[Indust. 3,3,3; Art. 2,3,3; Subs. 2,3,3; Recr 2,2,3; Disc. 2,3,3]
89	Gaza Strip	4	Abudaya, M., Harper, S., Ulman, A. and Zeller, D. 2013. Correcting mis- and under-reported marine fisheries catches for the Gaza Strip: 1950-2010. *Acta Adriatica* 54(2): 241-252.<br>[Indust. 3,4,4; Art. 3,3,4; Subs. 3,3,3; Recr 2,3,3; Disc. 2,3,3]
90	Georgia	1	Ulman, A. and Divovich, E. 2015. The marine fishery catch of Georgia (including Abkhazia), 1950-2010. Fisheries Centre Working Paper #2015-88, University of British Columbia, Vancouver, 25 p. (http://www.seaaroundus.org/doc/publications/wp/2015/Ulman-and-Divovich-Georgia.pdf).<br>[Indust. 2.5,3,1.5; Art. 3,4,2; Subs. 1,1,2; Recr. 1,1,2; Disc. -,-,-]
91	Germany (Baltic Sea)	2,4	(2) Rossing, P., Hammer, C., Bale, S., Harper, S., Booth, S. and Zeller, D. 2010. Germany's marine fisheries catches in the Baltic Sea (1950-2007), pp. 107-126. *In:* R. Rossing, S. Booth and D. Zeller (eds.) *Total marine fisheries extractions by country in the Baltic Sea: 1950-present.* Fisheries Centre Research Reports 18 (1), University of British Columbia, Vancouver. (http://www.seaaroundus.org/doc/publications/chapters/2010/Rossing-et-al-Germany-Baltic.pdf).<br>(4) Zeller, D., Rossing, P., Harper, S., Persson, L., Booth, S. and Pauly, D. 2011. The Baltic Sea: estimates of total fisheries removals 1950-2007. *Fisheries Research* 108: 356-363.<br>Since completing the initial reconstruction, ICES landing statistics became available to 2010. To update the reconstruction, ICES landing statistics for 2008-2010 were accepted as the reported landings. The unreported component was calculated using the 2007 IUU rates (by species), which were applied to the reported landings. To calculate discards, the 2007 discard rates (by species) were applied to the sum of reported landings and unreported catches. To calculate recreational catch, population data was first retrieved from Populstat (www.populstat.info), and if needed, a linear interpolation was used to estimate annual population. The 2007 per capita catch rate for the recreational sector was then applied to the 2008-2010 population estimates to calculate total recreational catch for those years. Please note that the values and comparisons for the years 1950-2007 were based on the 2007 ICES dataset, and changes were not made to account for small differences within the 2010 dataset regarding previous years.<br>[Indust. 2,2,2; Art. 2,2,2; Subs. 2,2,2; Recr. 1,1,1; Disc. 2,2,2]
92	Germany (North Sea)	1	Gibson, D., Froese, R., Ueberschaer, B., Zylich, K. and Zeller, D. 2014. Reconstruction of total marine fisheries catches for Germany in the North Sea (1950-2010). Fisheries Centre Working Paper #2015-09, University of British Columbia, Vancouver, 11 p. (http://www.seaaroundus.org/doc/publications/wp/2015/Gibson-et-al-GermanyNorthSea.pdf).<br>[Indust. 3,3,4; Art. 3,3,4; Subs. 1,1,1; Recr 1,1,2; Disc. 1,1,3]
93	Ghana	1,4,4,4	(1) Nunoo, F.K.E., Asiedu, B., Amador, K., Belhabib, D. and Pauly, D. 2015. Reconstruction of marine fisheries catches for Ghana, 1950-2010. Fisheries Centre Working Paper #2015-10, University of British Columbia, Vancouver, 16 p. (http://www.seaaroundus.org/doc/publications/wp/2014/Nunoo-et-al-Ghana.pdf).<br>(4) Nunoo, F.K.E., Asiedu, B., Amador, K., Belhabib, D., Lam, V.W.Y., Sumaila, U.R. and Pauly, D. 2014. Marine fisheries catches in Ghana: historic reconstruction for 1950 to 2010 and current economic impacts. *Reviews in Fisheries Science & Aquaculture* 22(4): 274-283.<br>(4) Belhabib, D., Sumaila, U.R., Lam, V.W.Y., Zeller, D., Le Billon, P., Kane, E.A. and Pauly, D. 2015. Euro vs. Yuan: Comparing European and Chinese fishing access in West Africa. *PLoS One* 10(3): e0118351<br>(4) Belhabib, D., Sumaila, U.R. and Pauly, D. 2015. Feeding the poor: contribution of West African fisheries to employment and food security. *Ocean & Coastal Management* 111: 72-81.<br>[Indust. 4,4,4; Art. 3,3,3; Subs. 2,2,3; Recr. 2,2,3; Disc. 2,3,3]
94	Greece	1	Moutopoulos, D.K., Tsikliras, A.C. and Stergiou, K.I. 2014. Reconstruction of Greek fishery catches by fishing gear and area (1950-2010). Fisheries Centre Working Paper #2015-11, University of British Columbia, Vancouver, 14 p. (http://www.seaaroundus.org/doc/publications/wp/2015/Moutopoulos-et-al-Greece.pdf).<br>[Indust. 3,3,3; Art. 2,2,3; Subs. 1,1,1; Recr. 1,1,2; Disc. 1,1,3]
95	Greece (Crete)		Moutopoulos, D.K., Tsikliras, A.C. and Stergiou, K.I. 2014. Reconstruction of Greek fishery catches by fishing gear and area (1950-2010). Fisheries Centre Working Paper #2015-11, University of British Columbia, Vancouver, 14 p. (http://www.seaaroundus.org/doc/publications/wp/2015/Moutopoulos-et-al-Greece.pdf).

Supplementary Table 5: Sources of reconstructions by country/EEZ component with associated publication links. Publication types are: 1: *Fisheries Centre Working Paper*, 2: *Fisheries Centre Research Reports*, 3: Other reports; 4: Primary literature. Uncertainty scores (as per Table S1) are given below the source of reconstruction for the three time periods 1950-1969, 1970-1989, 1990-2010 in square brackets by fishing sectors: Indust. = industrial, Art. = artisanal, Subs. = subsistence, Recr. = recreational, plus Disc. = discards.

#	Country	Publ. type	Source of reconstruction
			[Indust. 3,3,3; Art. 2,2,3; Subs. 1,1,1; Recr. 1,1,2; Disc. 1,1,3]
96	Greenland	2	Booth, S. and Knip, D. 2014. The catch of living marine resources around Greenland from 1950-2010. pp. 55-72. *In*: K. Zylich, D. Zeller, M. Ang and D. Pauly (eds.) *Fisheries catch reconstructions: Islands, Part IV.* Fisheries Centre Research Reports 22(2), University of British Columbia, Vancouver. (http://www.seaaroundus.org/doc/publications/chapters/2014/Booth-et-al-Greenland.pdf).<br>[Indust. 2,3,3; Art. 2,3,3; Subs. 2,2,2; Recr. 1,1,1; Disc. 3,3,3]
97	Grenada	1	Mohammed, E. and Lindop, A. 2015. Grenada: Reconstructed Fisheries Catches, 1950-2010. Fisheries Centre Working Paper #2015-40, University of British Columbia, Vancouver, 27 p. (http://www.seaaroundus.org/doc/publications/wp/2015/Mohammed-and-Lindop-Grenada.pdf).<br>[Indust. 3,3,3; Art. 2,3,3; Subs. 2,2,2; Recr. 1,1,1; Disc. 2,2,2]
98	Guatemala (Caribbean)	1	Lindop, A., Ixquiac-Cabrera, M., Zylich, K. and Zeller, D. 2015. A reconstruction of marine fish catches in the Republic of Guatemala. Fisheries Centre Working Paper #2015-41, University of British Columbia, Vancouver, 17 p. (http://www.seaaroundus.org/doc/publications/wp/2015/Lindop-et-al-Guatemala.pdf).<br>[Indust. 2,2,2; Art. 2,2,2; Subs. 1,1,1; Recr. 1,1,1; Disc. 1,1,1]
99	Guatemala (Pacific)	1	Lindop, A., Ixquiac-Cabrera, M., Zylich, K. and Zeller, D. 2015. A reconstruction of marine fish catches in the Republic of Guatemala. Fisheries Centre Working Paper #2015-41, University of British Columbia, Vancouver, 17 p. (http://www.seaaroundus.org/doc/publications/wp/2015/Lindop-et-al-Guatemala.pdf).<br>[Indust. 2,2,2; Art. 2,2,2; Subs. 1,1,1; Recr. 1,1,1; Disc. 1,1,1]
100	Guinea	2,3,4,4	(2) Belhabib, D., Doumbouya, A., Copeland, D., Gorez, B., Harper, S., Zeller, D. and Pauly, D. 2012. Guinean fisheries, past, present and…future? pp. 91-104 *In*: D. Belhabib, D. Zeller, S. Harper and D. Pauly (eds.) *Marine fisheries catches in West Africa, Part I.* Fisheries Centre Research Reports 20(3), University of British Columbia, Vancouver. (http://www.seaaroundus.org/doc/publications/chapters/Belhabib-et-al-Guinea.pdf).<br>(3) Belhabib, D., Doumbouya, A., Diallo, I., Traore, S., Camara, Y., Copeland, D., Gorez, B., Harper, S., Zeller, D., Sumaila, U.R. and Pauly, D. 2014. Guinean fisheries resources: an abused source of wealth. *In:* P. Onyango, S. Chimatiro and U.R. Sumaila (eds.) UNKNOWN WEALTH: The Contribution of Capture and Aquaculture fisheries in Accelerating Economic Growth and Food Security in Africa. Dordrecht, Springer Netherlands [in press].<br>(4) Belhabib, D., Sumaila, U.R., Lam, V.W.Y., Zeller, D., Le Billon, P., Kane, E.A. and Pauly, D. 2015. Euro vs. Yuan: Comparing European and Chinese fishing access in West Africa. *PLoS One* 10(3): e0118351<br>(4) Belhabib, D., Sumaila, U.R. and Pauly, D. 2015. Feeding the poor: contribution of West African fisheries to employment and food security. *Ocean & Coastal Management* 111: 72-81.<br>[Indust. 2,4,2; Art. 2,3,4; Subs. 2,2,2; Recr -,-,-; Disc. 4,4,4]
101	Guinea Bissau	2,4,4	(2) Belhabib, D. and Pauly, D. 2015. Fisheries in troubled waters: a catch reconstruction for Guinea-Bissau, 1950-2010. pp. 1-16. *In:* D. Belhabib and D. Pauly (eds). *Fisheries catch reconstructions: West Africa, Part II.* Fisheries Centre Research Reports 23(3), University of British Columbia(http://www.seaaroundus.org/doc/publications/chapters/2015/Belhabib-and-Pauly-Guinea-Bissau.pdf) (4) Belhabib, D., Sumaila, U.R., Lam, V.W.Y., Zeller, D., Le Billon, P., Kane, E.A. and Pauly, D. 2015. Euro vs. Yuan: Comparing European and Chinese fishing access in West Africa. *PLoS One* 10(3): e0118351<br>(4) Belhabib, D., Sumaila, U.R. and Pauly, D. 2015. Feeding the poor: contribution of West African fisheries to employment and food security. *Ocean & Coastal Management* 111: 72-81.<br>[Indust. 4,4,4; Art. 3,3,3; Subs. 2,3,3; Recr 2,2,4; Disc. 2,3,3]
102	Guyana	1	MacDonald, J., Harper, S., Booth, S. and Zeller, D. 2015. Guyana fisheries catch: 1950-2010. Fisheries Centre Working Paper #2015-21, University of British Columbia, Vancouver, 18 p. (http://www.seaaroundus.org/doc/publications/wp/2015/MacDonald-et-al-Guyana.pdf).<br>[Indust. 3,3,3; Art. 2,2,2; Subs. 1,1,1; Recr -,-,-; Disc. 1,2,2]
103	Haiti and Navassa Island	2	Ramdeen, R., Belhabib, D., Harper, S. and Zeller, D. 2012. Reconstruction of total marine fisheries catches for Haiti and Navassa Island (1950-2010). pp. 37-45. *In:* S. Harper, K. Zylich, L. Boonzaier, F. Le Manach, D. Pauly and D. Zeller (eds.) *Fisheries catch reconstructions: Islands, Part III.* Fisheries Centre Research Reports 20(5), University of British Columbia, Vancouver. (http://www.seaaroundus.org/doc/publications/chapters/2012/Ramdeen-et-al-Haiti-NavassaIsland.pdf).<br>[Indust. 2,2,2; Art. 2,2,2; Subs. 1,2,2; Recr. 1,1,1; Disc. 2,2,2]
104	Honduras (Caribbean)	1	Funes, M., Zylich, K., Divovich, E., Zeller, D., Lindop, A., Pauly, D. and Box, S. 2015. Honduras, a fish exporting country: Preliminary reconstructed marine catches in the Caribbean Sea and the Gulf of Fonseca, 1950 – 2010. Fisheries Centre Working Paper #2015-90,

Supplementary Table 5: Sources of reconstructions by country/EEZ component with associated publication links. Publication types are: 1: *Fisheries Centre Working Paper*, 2: *Fisheries Centre Research Reports*, 3: Other reports; 4: Primary literature. Uncertainty scores (as per Table S1) are given below the source of reconstruction for the three time periods 1950-1969, 1970-1989, 1990-2010 in square brackets by fishing sectors: Indust. = industrial, Art. = artisanal, Subs. = subsistence, Recr. = recreational, plus Disc. = discards.

#	Country	Publ. type	Source of reconstruction
			University of British Columbia, Vancouver, 16 p. (http://www.seaaroundus.org/doc/publications/wp/2015/Funes-et-al-Honduras.pdf). [Indust. 2,2,2; Art. 2,2,2; Subs. 1,1,1; Recr. 1,1,1; Disc. 2,2,2]
105	Honduras (Pacific)	1	Funes, M., Zylich, K., Divovich, E., Zeller, D., Lindop, A., Pauly, D. and Box, S. 2015. Honduras, a fish exporting country: Preliminary reconstructed marine catches in the Caribbean Sea and the Gulf of Fonseca, 1950 – 2010. Fisheries Centre Working Paper #2015-90, University of British Columbia, Vancouver, 16 p. (http://www.seaaroundus.org/doc/publications/wp/2015/Funes-et-al-Honduras.pdf). [Indust. 2,2,2; Art. 2,2,2; Subs. 1,1,1; Recr. 1,1,1; Disc. 2,2,2]
106	Iceland	2	Valtýsson, H. 2014. Reconstructing Icelandic catches from 1950-2010. pp. 73-88. *In:* K. Zylich, D. Zeller, M. Ang and D. Pauly (eds.) *Fisheries catch reconstructions: Islands, Part IV.* Fisheries Centre Research Reports 22(2), University of British Columbia, Vancouver. (http://www.seaaroundus.org/doc/publications/chapters/2014/Valtýsson-Iceland.pdf). [Indust. 4,4,4; Art. 2,3,4; Subs. 1,2,3; Recr 1,2,3; Disc. 1,2,3]
107	India	1	Hornby, C., Bhathal, B., Pauly, D. and Zeller, D. 2015. Reconstruction of India's marine fish catch from 1950-2010. Fisheries Centre Working Paper #2015-77, University of British Columbia, Vancouver, 42 p. (http://www.seaaroundus.org/doc/publications/wp/2015/Hornby-et-al-India.pdf). [Indust. 2,2,2; Art. 2,3,3; Subs. 2,2,2; Recr. 1,1,1; Disc. 1,1,1]
108	India (Andaman and Nicobar Islands)	1	Hornby, C., Arun Kumar, M., Bhathal, B., Pauly, D. and Zeller, D. 2015. Reconstruction of the Andaman and Nicobar Islands marine fish catch from 1950-2010. Fisheries Centre Working Paper #2015-75, University of British Columbia, Vancouver, 27 p. (http://www.seaaroundus.org/doc/publications/wp/2015/Hornby-et-al-AN-Islands.pdf). [Indust. 1,2,1; Art. 1,2,1; Subs. 1,2,1; Recr. 1,1,1; Disc. 1,1,1]
109	Indonesia (Central)	1	Budimartono, V., Badrudin, M., Divovich, E. and Pauly, D. 2015. A reconstruction of marine fisheries catches of Indonesia, with emphasis on Central and Eastern Indonesia, 1950 – 2010. pp. 2-26 *In:* D. Pauly and V. Budimartono (eds.) *Marine Fisheries Catches of Western, Central and Eastern Indonesia, 1950-2010*. Fisheries Centre Working Paper #2015-61, University of British Columbia, Vancouver. (http://www.seaaroundus.org/doc/publications/wp/2015/Pauly-and-Budimartono-Indonesia.pdf). [Indust. 1,2,1; Art. 1,2,1; Subs. 1,2,1; Recr. 1,1,1; Disc. 1,1,1]
110	Indonesia (Eastern)	1	Budimartono, V., Badrudin, M., Divovich, E. and Pauly, D. 2015. A reconstruction of marine fisheries catches of Indonesia, with emphasis on Central and Eastern Indonesia, 1950 – 2010. pp. 2-26 *In:* D. Pauly and V. Budimartono (eds.) *Marine Fisheries Catches of Western, Central and Eastern Indonesia, 1950-2010.* Fisheries Centre Working Paper #2015-61, University of British Columbia, Vancouver. (http://www.seaaroundus.org/doc/publications/wp/2015/Pauly-and-Budimartono-Indonesia.pdf). [Indust. 1,2,1; Art. 1,2,1; Subs. 1,2,1; Recr. 1,1,1; Disc. 1,1,1]
111	Indonesia (Indian Ocean)	1	Budimartono, V., Badrudin, M. and Pauly, D. 2015. Indonesian marine fisheries catches in the Western Indonesia (FAO Area 57) and in the Bay of Bengal large marine ecosystem project (BOBLME) area: a tentative reconstruction, 1950-2010. pp. 27-51. *In:* D. Pauly and V. Budimartono (eds.) *Marine Fisheries Catches of Western, Central and Eastern Indonesia, 1950-2010.* Fisheries Centre Working Paper #2015-61, University of British Columbia, Vancouver. (http://www.seaaroundus.org/doc/publications/wp/2015/Pauly-and-Budimartono-Indonesia.pdf). [Indust. 2,2,2; Art. 2,2,2; Subs. 1,1,1; Recr. 1,1,1; Disc. 1,1,1]
112	Iran (Arabian Sea)	2,4	(2) Roshan Moniri, Nar., Roshan Moniri, Naz., Zeller, D., Al-Abdulrazzak, D. and Belhabib, D. 2013. Fisheries catch reconstruction for Iran, 1950-2010. pp. 7-16. *In*: D. Al-Abdulrazzak and D. Pauly (eds.) *From dhows to trawlers: a recent history of fisheries in the Gulf countries, 1950 to 2010.* Fisheries Centre Research Reports 21(2), University of British Columbia, Vancouver. (http://www.seaaroundus.org/doc/publications/chapters/2013/Moniri-et-al-Iran.pdf).<br>(4) Al-Abdulrazzak, D., Zeller, D., Belhabib, D., Tesfamichael, D. and Pauly, D. 2015. Total marine fisheries catches in the Persian/Arabian Gulf from 1950-2010. *Regional Studies in Marine Science* 2: 28-34.<br>[Indust. 2,3,2; Art. 2,3,2; Subs. 2,2,2; Recr. 1,1,1; Disc. 1,1,1]
113	Iran (Persian Gulf)	2,4	(2) Roshan Moniri, Nar., Roshan Moniri, Naz., Zeller, D., Al-Abdulrazzak, D. and Belhabib, D. 2013. Fisheries catch reconstruction for Iran, 1950-2010. pp. 7-16. *In*: D. Al-Abdulrazzak and D. Pauly (eds.) *From dhows to trawlers: a recent history of fisheries in the Gulf countries, 1950 to 2010.* Fisheries Centre Research Reports 21(2), University of British Columbia, Vancouver. (http://www.seaaroundus.org/doc/publications/chapters/2013/Moniri-et-al-Iran.pdf).

Supplementary Table 5: Sources of reconstructions by country/EEZ component with associated publication links. Publication types are: 1: *Fisheries Centre Working Paper*, 2: *Fisheries Centre Research Reports*, 3: Other reports; 4: Primary literature. Uncertainty scores (as per Table S1) are given below the source of reconstruction for the three time periods 1950-1969, 1970-1989, 1990-2010 in square brackets by fishing sectors: Indust. = industrial, Art. = artisanal, Subs. = subsistence, Recr. = recreational, plus Disc. = discards.

#	Country	Publ. type	Source of reconstruction
			(4) Al-Abdulrazzak, D., Zeller, D., Belhabib, D., Tesfamichael, D. and Pauly, D. 2015. Total marine fisheries catches in the Persian/Arabian Gulf from 1950-2010. *Regional Studies in Marine Science* 2: 28-34.<br>[Indust. 2,3,2; Art. 2,3,2; Subs. 2,2,2; Recr. 1,1,1; Disc. 1,1,1]
114	Iraq	2,4	(2) Al-Abdulrazzak, D. and Pauly, D. 2013. Reconstructing Iraq's fisheries: 1950-2010. pp. 17-22. *In*: D. Al-Abdulrazzak and D. Pauly (eds.) *From dhows to trawlers: a recent history of fisheries in the Gulf countries, 1950 to 2010.* Fisheries Centre Research Reports 21(2), University of British Columbia, Vancouver. (http://www.seaaroundus.org/doc/publications/chapters/2013/AlAbdulrazzak-et-al-Iraq.pdf).<br>(4) Al-Abdulrazzak, D., Zeller, D., Belhabib, D., Tesfamichael, D. and Pauly, D. 2015. Total marine fisheries catches in the Persian/Arabian Gulf from 1950-2010. *Regional Studies in Marine Science* 2: 28-34.<br>[Indust. -,-,-; Art. 2,2,2; Subs. 1,1,1; Recr -,-,-; Disc. 1,1,1]
115	Ireland	1	Miller, D. and Zeller, D. 2013. Reconstructing Ireland's marine fisheries catches: 1950-2010. Fisheries Centre Working Paper #2013-10, University of British Columbia, Vancouver, 48 p. (http://www.seaaroundus.org/doc/publications/wp/2013/Miller-et-al-Ireland.pdf).<br>[Indust. 3,3,4; Art. 2,2,3; Subs. 3,3,3; Recr 1,1,1; Disc. 2,3,4]
116	Israel (Mediterranean)	4	Edelist, D., Scheinin, A., Sonin, O., Shapiro, J., Salameh, P., Rilov, G., Benayhu, Y., Schulz, D. and Zeller, D. 2013. Israel: Reconstructed estimates of total fisheries removals in the Mediterranean, 1950–2010. *Acta Adriatica* 54(2): 252-264.<br>[Indust. 3,3,3; Art. 2,2,2; Subs. 1,1,1; Recr. 1,1,2; Disc. 1,1,2]
117	Israel (Red Sea)	2	Tesfamichael, D., Govender, R. and Pauly, D. 2012. Preliminary reconstruction of fisheries catches of Jordan and Israel in the inner Gulf of Aqaba, Red Sea, 1950-2010. pp. 179-204. *In: D.* Tesfamichael and D. Pauly (eds.) *Catch reconstruction for the Red Sea large marine ecosystem by countries (1950 – 2010).* Fisheries Centre Research Reports 20(1), University of British Columbia, Vancouver. (http://www.seaaroundus.org/doc/publications/chapters/2012/Tesfamichael-et-al-JordanIsrael.pdf).<br>[Indust. 3,3,3; Art. 2,2,2; Subs. 1,1,1; Recr. 1,1,2; Disc. 1,1,2]
118	Italy	1,4,4	(1) Piroddi, C., Gristina, M., Ulman, A., Zeller, D. and Pauly, D. 2014. Reconstruction of Italy's marine fisheries catches (1950-2010). Fisheries Centre Working Paper #2014-22, University of British Columbia, Vancouver, 42 p. (http://www.seaaroundus.org/doc/publications/wp/2014/Piroddi-et-al-Italy.pdf).<br>(4) Pauly, D., Ulman, A., Piroddi, C., Bultel, E. and Coll, M. 2014. 'Reported' versus 'likely' fisheries catches of four Mediterranean countries. pp. 11-17. *In:* J. Lleonart and F. Maynou (eds.) The Ecosystem approach to fisheries in the Mediterranean and Black Seas. *Scientia Marina* 78S1.<br>(4) Piroddi, C., Gristina, M., Zylich, K., Greer, K., Ulman, A., Zeller, D. and Pauly, D. 2015. Reconstruction of Italy's marine fisheries removals and fishing capacity, 1950-2010. *Fisheries Research* 172: 137-147.<br>[Indust. 1.5,2.5,3.5; Art. 2,3,4; Subs. 1,1,1; Recr 1,1,2; Disc. -,-,-]
119	Italy (Sardinia)	1,4	(1) Piroddi, C., Gristina, M., Ulman, A., Zeller, D. and Pauly, D. 2014. Reconstruction of Italy's marine fisheries catches (1950-2010). Fisheries Centre Working Paper #2014-22, University of British Columbia, Vancouver, 42 p. (http://www.seaaroundus.org/doc/publications/wp/2014/Piroddi-et-al-Italy.pdf).<br>(4) Piroddi, C., Gristina, M., Zylich, K., Greer, K., Ulman, A., Zeller, D. and Pauly, D. 2015. Reconstruction of Italy's marine fisheries removals and fishing capacity, 1950-2010. *Fisheries Research* 172: 137-147.<br>[Indust. 1.5,2.5,3.5; Art. 2,3,4; Subs. 1,1,1; Recr 1,1,2; Disc. -,-,-]
120	Italy (Sicily)	1,4	(1) Piroddi, C., Gristina, M., Ulman, A., Zeller, D. and Pauly, D. 2014. Reconstruction of Italy's marine fisheries catches (1950-2010). Fisheries Centre Working Paper #2014-22, University of British Columbia, Vancouver, 42 p. (http://www.seaaroundus.org/doc/publications/wp/2014/Piroddi-et-al-Italy.pdf).<br>(4) Piroddi, C., Gristina, M., Zylich, K., Greer, K., Ulman, A., Zeller, D. and Pauly, D. 2015. Reconstruction of Italy's marine fisheries removals and fishing capacity, 1950-2010. *Fisheries Research* 172: 137-147.<br>[Indust. 1.5,2.5,3.5; Art. 2,3,4; Subs. 1,1,1; Recr 1,1,2; Disc. -,-,-]
121	Jamaica	2	Lingard, S., Harper, S., Aiken, K., Hado, N., Smikle, S. and Zeller, D. 2012. Marine fisheries of Jamaica: total reconstructed catch 1950-2010, pp. 47-59. *In:* S. Harper, K. Zylich, L. Boonzaier, F. Le Manach, D. Pauly and D. Zeller (eds.) *Fisheries catch reconstructions: Islands, Part III.* Fisheries Centre Research Reports 20(5), University of British Columbia, Vancouver, 42 p. (http://www.seaaroundus.org/doc/publications/chapters/2012/Lingard-et-al-Jamaica.pdf).

Supplementary Table 5: Sources of reconstructions by country/EEZ component with associated publication links. Publication types are: 1: *Fisheries Centre Working Paper*, 2: *Fisheries Centre Research Reports*, 3: Other reports; 4: Primary literature. Uncertainty scores (as per Table S1) are given below the source of reconstruction for the three time periods 1950-1969, 1970-1989, 1990-2010 in square brackets by fishing sectors: Indust. = industrial, Art. = artisanal, Subs. = subsistence, Recr. = recreational, plus Disc. = discards.

#	Country	Publ. type	Source of reconstruction
			[Indust. 2,2,2; Art. 2,2,2; Subs. 1,1,1; Recr. 1,1,1; Disc. 1,1,1]
122	Japan	4	Swartz, W. and Ishimura, G. 2014. Baseline assessment of total fisheries-related biomass removal from Japan's Exclusive Economic Zone: 1950-2010. *Fisheries Science*, doi: 10.1007/s12562-014-0754-6<br>[Indust. 2.5,4,4; Art. 4,4,4; Subs. 2,2,2; Recr 1,2,4; Disc. 1,2,2]
123	Japan (Daito Islands)	1	Swartz, W. 2015. Notes on the fisheries around Japan's so-called 'outer-' or oceanic islands: Ogasawara (Bonin) and Daito Islands. Fisheries Centre Working Paper #2015-17, University of British Columbia, Vancouver, 5 p. (http://www.seaaroundus.org/doc/publications/wp/2015/Swartz-et-al-Japan-Outer.pdf).<br>[Indust. -,-,-; Art. 2.5,3.5,3.5; Subs. 2.5,3.5,3.5; Recr. -,-,-; Disc. -,-,-]
124	Japan (Ogasawara Islands)	1	Swartz, W. 2015. Notes on the fisheries around Japan's so-called 'outer-' or oceanic islands: Ogasawara (Bonin) and Daito Islands. Fisheries Centre Working Paper #2015-17, University of British Columbia, Vancouver, 5 p. (http://www.seaaroundus.org/doc/publications/wp/2015/Swartz-et-al-Japan-Outer.pdf).<br>[Indust. -,-,-; Art. 1.5,1.5,3.5; Subs. 1.5,1.5,3.5; Recr. -,-,-; Disc. -,-,-]
125	Jordan	2	Tesfamichael, D., Govender, R. and Pauly, D. 2012. Preliminary reconstruction of fisheries catches of Jordan and Israel in the inner Gulf of Aqaba, Red Sea, 1950-2010. pp. 179-204. *In:* D. Tesfamichael and D. Pauly (eds.) *Catch reconstruction for the Red Sea large marine ecosystem by countries (1950 – 2010).* Fisheries Centre Research Reports 20(1), University of British Columbia, Vancouver. (http://www.seaaroundus.org/doc/publications/chapters/2012/Tesfamichael-et-al-JordanIsrael.pdf).<br>[Indust. 1,4,4; Art. 1,1,1; Subs. 1,1,2; Recr. 2,2,2; Disc. 3,3,3]
126	Kenya	2	Le Manach, F., Abunge, C.A., McClanahan, T.R. and Pauly, D. 2015. Tentative Reconstruction of Kenya's Marine Fisheries Catch, 1950–2010. pp. 37-52. *In:* F. Le Manach and D. Pauly (eds.) *Fisheries catch reconstructions in the Western Indian Ocean, 1950-2010.* Fisheries Centre Research Report 23(2), University of British Columbia, Vancouver. (http://www.seaaroundus.org/doc/publications/chapters/2015/LaManach-et-al-2015-Kenya.pdf).<br>[Indust. 2,3,3; Art. 2,3,3; Subs. 1,2,2; Recr. 2,2,2; Disc. 2,2,2]
127	Kiribati (Gilbert Islands)	2,4	(2) Zylich, K., Harper, S. and Zeller, D. 2014. Reconstruction of marine fisheries catches for the Republic of Kiribati (1950-2010). pp. 89-106. *In:* K. Zylich, D. Zeller, M. Ang, and D. Pauly (eds.) *Fisheries catch reconstructions: Islands, Part IV.* Fisheries Centre Research Reports 22(2), University of British Columbia, Vancouver. (http://www.seaaroundus.org/doc/publications/chapters/2014/Zylich-et-al-Kiribati.pdf).<br>(4) Zeller, D., Harper, S., Zylich, K. and Pauly, D. 2015. Synthesis of under-reported small-scale fisheries catch in Pacific island waters. *Coral Reefs* 34(1): 25-39.<br>[Indust. 3,3,3; Art. 2,2,2; Subs. 2,2,2; Recr. 1,1,1; Disc. 3,3,3]
128	Kiribati (Line Islands)	2,4	(2) Zylich, K., Harper, S. and Zeller, D. 2014. Reconstruction of marine fisheries catches for the Republic of Kiribati (1950-2010). pp. 89-106. *In*: K. Zylich, D. Zeller, M. Ang, and D. Pauly (eds.) *Fisheries catch reconstructions: Islands, Part IV.* Fisheries Centre Research Reports 22(2), University of British Columbia, Vancouver. (http://www.seaaroundus.org/doc/publications/chapters/2014/Zylich-et-al-Kiribati.pdf).<br>(4) Zeller, D., Harper, S., Zylich, K. and Pauly, D. 2015. Synthesis of under-reported small-scale fisheries catch in Pacific island waters. *Coral Reefs* 34(1): 25-39.<br>[Indust. 3,3,3; Art. 2,2,2; Subs. 2,2,2; Recr. 1,1,1; Disc. 3,3,3]
129	Kiribati (Phoenix Islands)	2,4	Zylich, K., Harper, S. and Zeller, D. 2014. Reconstruction of marine fisheries catches for the Republic of Kiribati (1950-2010). pp. 89-106. *In*: K. Zylich, D. Zeller, M. Ang, and D. Pauly (eds.) *Fisheries catch reconstructions: Islands, Part IV.* Fisheries Centre Research Reports 22(2), University of British Columbia, Vancouver. (http://www.seaaroundus.org/doc/publications/chapters/2014/Zylich-et-al-Kiribati.pdf).<br>(4) Zeller, D., Harper, S., Zylich, K. and Pauly, D. 2015. Synthesis of under-reported small-scale fisheries catch in Pacific island waters. *Coral Reefs* 34(1): 25-39.<br>[Indust. 3,3,3; Art. 2,2,2; Subs. 2,2,2; Recr. 1,1,1; Disc. 3,3,3]
130	Korea (North)	1	Shon, S., Harper, S. and Zeller, D. 2014. Reconstruction of Marine Fisheries Catches for the Republic of Korea (South Korea) from 1950-2010. Fisheries Centre Working Paper #2014-19, University of British Columbia, Vancouver, 13 p. (http://www.seaaroundus.org/doc/publications/wp/2014/Shon-et-al-North-Korea.pdf).<br>[Indust. 2,2,2; Art. 1,1,1; Subs. 1,1,1; Recr. -,-,-; Disc. 1,1,1]
131	Korea (South)	1	Shon, S., Harper, S. and Zeller, D. 2014. Reconstruction of marine fisheries catches from the Democratic People's Republic of Korea (North Korea) from 1950-2010. Fisheries Centre Working Paper #2014-20, University of British Columbia, Vancouver, 11 p. (http://www.seaaroundus.org/doc/publications/wp/2014/Shon-et-al-South-Korea.pdf).<br>[Indust. 2,2,3; Art. 2,2,3; Subs. 2,2,2; Recr. 1,1,2; Disc. 2,2,2]

Supplementary Table 5: Sources of reconstructions by country/EEZ component with associated publication links. Publication types are: 1: *Fisheries Centre Working Paper*, 2: *Fisheries Centre Research Reports*, 3: Other reports; 4: Primary literature. Uncertainty scores (as per Table S1) are given below the source of reconstruction for the three time periods 1950-1969, 1970-1989, 1990-2010 in square brackets by fishing sectors: Indust. = industrial, Art. = artisanal, Subs. = subsistence, Recr. = recreational, plus Disc. = discards.

#	Country	Publ. type	Source of reconstruction
132	Kuwait	2,4	(2) Al-Abdulrazzak, D. 2013. Reconstructing Kuwait's marine fishery catches: 1950-2010. pp. 23-29. *In*: D. Al-Abdulrazzak and D. Pauly (eds.) *From dhows to trawlers: a recent history of fisheries in the Gulf countries, 1950 to 2010.* Fisheries Centre Research Reports 21(2), University of British Columbia, Vancouver. (http://www.seaaroundus.org/doc/publications/chapters/2013/AlAbdulrazzak-et-al-Kuwait.pdf).<br>(4) Al-Abdulrazzak, D., Zeller, D., Belhabib, D., Tesfamichael, D. and Pauly, D. 2015. Total marine fisheries catches in the Persian/Arabian Gulf from 1950-2010. *Regional Studies in Marine Science* 2: 28-34.<br>[Indust. 2,2,2; Art. 2,2,3; Subs. 1,1,1; Recr 2,2,2; Disc. 3,3,3]
133	Latvia	2,4	(2) Rossing, P., Plikshs, M., Booth, S., Veitch, L. and Zeller, D. 2010. Catch reconstruction for Latvia in the Baltic Sea from 1950 – 2007. pp. 127-144. *In:* R. Rossing, S. Booth and D. Zeller (eds.) *Total marine fisheries extractions by country in the Baltic Sea: 1950-present.* Fisheries Centre Research Reports 18 (1), University of British Columbia, Vancouver. (http://www.seaaroundus.org/doc/publications/chapters/2010/Rossing-et-al-Latvia.pdf).<br>(4) Zeller, D., Rossing, P., Harper, S., Persson, L., Booth, S. and Pauly, D. 2011. The Baltic Sea: estimates of total fisheries removals 1950-2007. *Fisheries Research* 108: 356-363.<br>Since completing the initial reconstruction, ICES landing statistics became available to 2010. To update the reconstruction, ICES landing statistics for 2008-2010 were accepted as the reported landings. The unreported component was calculated using the 2007 IUU rates (by species), which were applied to the reported landings. To calculate discards, the 2007 discard rates (by species) were applied to the sum of reported landings and unreported catches. To calculate recreational catch, population data was first retrieved from Populstat (www.populstat.info), and if needed, a linear interpolation was used to estimate annual population. The 2007 per capita catch rate for the recreational sector was then applied to the 2008-2010 population estimates to calculate total recreational catch for those years. Please note that the values and comparisons for the years 1950-2007 were based on the 2007 ICES dataset, and changes were not made to account for small differences within the 2010 dataset regarding previous years.<br>[Indust. 2,3,3; Art. 2,3,3; Subs. 2,2,2; Recr. 1,2,2; Disc. 2,2,2]
134	Lebanon	1	Nader, M.R., Indary, S., Roshan Moniri, Naz. and Zylich, K. 2014. Historical fisheries catch reconstruction for Lebanon (GSA 27), 1950-2010. Fisheries Centre Working Paper #2014-11, University of British Columbia, Vancouver, 19 p. (http://www.seaaroundus.org/doc/publications/wp/2014/Nader-et-al-Lebanon.pdf).<br>[Indust. 2,2,2; Art. 2,2,2; Subs. 1,1,1; Recr. 1,1,1; Disc. 1,1,1]
135	Liberia	1,4,4,4	(1) Belhabib, D., Subah, Y., Broh, N.T., Jueseah, A.S., Nipey, N., Boeh, W.Y., Copeland, D., Zeller, D. and Pauly, D. 2013. When 'Reality leaves a lot to the imagination': Liberian fisheries from 1950 to 2010. Fisheries Centre Working Paper #2013-06, University of British Columbia, Vancouver, 18 p. (http://www.seaaroundus.org/doc/publications/wp/2013/Belhabib-et-al-2013-Liberia.pdf).<br>(4) Belhabib, D., Sumaila, U.R., Lam, V.W.Y., Zeller, D., Le Billon, P., Kane, E.A. and Pauly, D. 2015. Euro vs. Yuan: Comparing European and Chinese fishing access in West Africa. *PLoS One* 10(3): e0118351<br>(4) Belhabib, D., Sumaila, U.R. and Pauly, D. 2015. Feeding the poor: contribution of West African fisheries to employment and food security. *Ocean & Coastal Management* 111: 72-81.<br>(4) Belhabib, D., Mendy, A., Subah, Y., Broh, N.T., Jueseah, A.S., Nipey, N., Boeh, W.W., Willemse, N., Zeller, D. and Pauly, D. (in press) Fisheries catch under-reporting in The Gambia, Liberia and Namibia, and the three Large Marine Ecosystems which they represent. *Environmental Development.* DOI: 10.1016/j.envdev.2015.08.004<br>[Indust. 3,3,4; Art. 3,3,3; Subs. 1,3,3; Recr -,-,-; Disc. 2,2,4]
136	Libya	1	Khalfallah, M., Belhabib, D., Zeller, D. and Pauly, D. 2015. Reconstruction of Marine Fisheries catches for Libya (1950-2010). Fisheries Centre Working Paper #2015-47, University of British Columbia, Vancouver, 15 p. (http://www.seaaroundus.org/doc/publications/wp/2015/Khalfallah-et-al-Libya.pdf).<br>[Indust. 1,2,2; Art. 1,2,2; Subs. 1,2,2; Recr. 1,1,1; Disc. 1,2,2]
137	Lithuania	2,4	(2) Veitch, L., Toliusis, S., Booth, S., Rossing, P., Harper, S. and Zeller, D. 2010. Catch reconstruction for Lithuania in the Baltic Sea from 1950–2007. pp. 145-164. *In:* R. Rossing, S. Booth and D. Zeller (eds.) *Total marine fisheries extractions by country in the Baltic Sea: 1950-present.* Fisheries Centre Research Reports 18(1), University of British Columbia, Vancouver. (http://www.seaaroundus.org/doc/publications/chapters/2010/Veitch-et-al-Lithuania.pdf).<br>(4) Zeller, D., Rossing, P., Harper, S., Persson, L., Booth, S. and Pauly, D. 2011. The Baltic Sea: estimates of total fisheries removals 1950-2007. *Fisheries Research* 108: 356-363.<br>Since completing the initial reconstructions, ICES landing statistics became available to 2010. To update the reconstructions, ICES landing statistics for 2008-2010 were accepted as the reported landings. The unreported components were calculated using the 2007 IUU rates (by species), which

Supplementary Table 5: Sources of reconstructions by country/EEZ component with associated publication links. Publication types are: 1: *Fisheries Centre Working Paper*, 2: *Fisheries Centre Research Reports*, 3: Other reports; 4: Primary literature. Uncertainty scores (as per Table S1) are given below the source of reconstruction for the three time periods 1950-1969, 1970-1989, 1990-2010 in square brackets by fishing sectors: Indust. = industrial, Art. = artisanal, Subs. = subsistence, Recr. = recreational, plus Disc. = discards.

#	Country	Publ. type	Source of reconstruction
			were applied to the reported landings. To calculate discards, the 2007 discard rates (by species) were applied to the sum of reported landings and unreported catches. To calculate recreational catches, population data were first retrieved from Populstat (www.populstat.info), and if needed, linear interpolations were used to estimate annual populations. The 2007 per capita catch rates for the recreational sectors were then applied to the 2008-2010 population estimates to calculate total recreational catches for those years. Please note that the values and comparisons for the years 1950-2007 were based on the 2007 ICES dataset, and changes were not made to account for small differences within the 2010 dataset regarding previous years. Data generated in these reconstructions (for both reported and unreported catches) for 1950 to 2010 will replace FAO landings data at www.seaaroundus.org.<br>[Indust. 3,3,4; Art. -,-,-; Subs. -,-,-; Recr. 3,3,2; Disc. 2,2,3]
138	Madagascar	2,4	(2) Le Manach, F., Gough, C., Humber, F., Harper, S. and Zeller, D. 2011. Reconstruction of total marine fisheries catches for Madagascar (1950-2008), pp. 21-42 *In:* S. Harper and D. Zeller (eds.) *Fisheries catch reconstruction: Islands, Part II.* Fisheries Centre Research Reports 19(4), University of British Columbia, Vancouver. (http://www.seaaroundus.org/doc/publications/chapters/2011/LeMenach-et-al-Madagascar.pdf).<br>(4) Le Manach, F., Gough, C., Harris, A., Humber, F., Harper, S. and Zeller, D. 2012. Unreported fishing, hungry people and political turmoil: the recipe for a food security crisis in Madagascar? *Marine Policy* 36(1): 218-225.<br>[Indust. 1,2,3; Art. 2,2,2; Subs. 2,2,2; Recr. 1,1,1; Disc. 2,2,2]
139	Malaysia (Peninsular)	1	Teh, L.C.L. and Teh, L.S.L. 2014. Reconstructing the marine fisheries catch of Peninsular Malaysia, Sarawak and Sabah, 1950-2010. Fisheries Centre Working Paper #2014-16, University of British Columbia, Vancouver, 20 p. (http://www.seaaroundus.org/doc/publications/wp/2014/Teh-et-al-Malaysia.pdf).<br>[Indust. 2,2,3; Art. 2,2,3; Subs. 2,2,2; Recr. 1,1,1; Disc. 2,2,3]
140	Malaysia (Sabah)	1,2	(1) Teh, L.C.L. and Teh, L.S.L. 2014. Reconstructing the marine fisheries catch of Peninsular Malaysia, Sarawak and Sabah, 1950-2010. Fisheries Centre Working Paper #2014-16, University of British Columbia, Vancouver, 20 p. (http://www.seaaroundus.org/doc/publications/wp/2014/Teh-et-al-Malaysia.pdf).<br>(2) Teh, L.S.L, Teh, L.C.L., Zeller, D. and Cabanban, A. 2009. Historical perspective of Sabah's marine fisheries. pp. 77-98. *In:* D. Zeller and S. Harper (eds). *Fisheries catch reconstructions: Islands, Part I.* Fisheries Centre Research Reports 17(5), University of British Columbia, Vancouver. (http://www.seaaroundus.org/doc/publications/chapters/2009/Teh-et-al-Sabah.pdf).<br>[Indust. -,2,3; Art. 2,2,2; Subs. 2,2,2; Recr -,-,-; Disc. -,3,3]
141	Malaysia (Sarawak)	1	Teh, L.C.L. and Teh, L.S.L. 2014. Reconstructing the marine fisheries catch of Peninsular Malaysia, Sarawak and Sabah, 1950-2010. Fisheries Centre Working Paper #2014-16, University of British Columbia, Vancouver, 20 p. (http://www.seaaroundus.org/doc/publications/wp/2014/Teh-et-al-Malaysia.pdf).<br>[Indust. -,2,3; Art. 2,2,2; Subs. 2,2,2; Recr -,1,1; Disc. -,3,3]
142	Maldives	2	Hemmings, M., Harper, S. and Zeller, D. 2014. Reconstruction of total marine catches for the Maldives: 1950–2010. pp. 107-120. *In:* K. Zylich, D. Zeller, M. Ang, and D. Pauly (eds.) *Fisheries catch reconstructions: Islands, Part IV.* Fisheries Centre Research Reports 22(2). Fisheries Centre, University of British Columbia, Vancouver. (http://www.seaaroundus.org/doc/publications/chapters/2014/Hemmings-et-al-Maldives.pdf).<br>[Indust. 2,3,3; Art. 2,3,3; Subs. 2,3,3; Recr. 1,1,1; Disc. 2,3,3]
143	Malta	1	Khalfallah, M., Dimech, M., Ulman, A., Zeller, D. and Pauly, D. 2014. Reconstruction of Marine Fisheries catches for the Republic of Malta (1950-2010). Fisheries Centre Working Paper #2015-43, University of British Columbia, Vancouver, 12 p. (http://www.seaaroundus.org/doc/publications/wp/2015/Khalfallah-et-al-Malta.pdf).<br>[Indust. 2.5,2.5,3.5; Art. 3,3,4; Subs. 2,2,2; Recr 2,2,4; Disc. -,-,-]
144	Marshall Islands	2,4	(2) Haas, A., Harper, S., Zylich, K., Hehre, J. and Zeller, D. 2014. Reconstruction of the Republic of the Marshall Islands fisheries catches: 1950-2010. pp. 121-128. *In*: K. Zylich, D. Zeller, M. Ang, and D. Pauly (eds.) *Fisheries catch reconstructions: Islands, Part IV.* Fisheries Centre Research Reports 22(2). Fisheries Centre, University of British Columbia, Vancouver. (http://www.seaaroundus.org/doc/publications/chapters/2014/Haas-et-al-Marshall-Is.pdf).<br>(4) Zeller, D., Harper, S., Zylich, K. and Pauly, D. 2015. Synthesis of under-reported small-scale fisheries catch in Pacific island waters. *Coral Reefs* 34(1): 25-39.<br>[Indust. 4,3,4; Art. 2,2,3; Subs. 2,2,3; Recr -,-,-; Disc. 3,3,2]
145	Mauritania	2,4,4	(2) Belhabib, D., Gascuel, D., Abou Kane, E., Harper, S., Zeller, D. and Pauly, D. 2012. Preliminary estimation of realistic fisheries removals from Mauritania: 1950-2010. pp. 61-

Supplementary Table 5: Sources of reconstructions by country/EEZ component with associated publication links. Publication types are: 1: *Fisheries Centre Working Paper*, 2: *Fisheries Centre Research Reports*, 3: Other reports; 4: Primary literature. Uncertainty scores (as per Table S1) are given below the source of reconstruction for the three time periods 1950-1969, 1970-1989, 1990-2010 in square brackets by fishing sectors: Indust. = industrial, Art. = artisanal, Subs. = subsistence, Recr. = recreational, plus Disc. = discards.

#	Country	Publ. type	Source of reconstruction
			78 *In:* D. Belhabib, D. Zeller, S. Harper and D. Pauly (eds.) *Marine fisheries catches in West Africa, Part 1*. Fisheries Centre Research Reports 20(3), University of British Columbia, Vancouver. (http://www.seaaroundus.org/doc/publications/chapters/2012/Belhabib-et-al-2012-Mauritania.pdf).<br>(4) Belhabib, D., Sumaila, U.R., Lam, V.W.Y., Zeller, D., Le Billon, P., Kane, E.A. and Pauly, D. 2015. Euro vs. Yuan: Comparing European and Chinese fishing access in West Africa. *PLoS One* 10(3): e0118351<br>(4) Belhabib, D., Sumaila, U.R. and Pauly, D. 2015. Feeding the poor: contribution of West African fisheries to employment and food security. *Ocean & Coastal Management* 111: 72-81.<br>[Indust. 2,2,4; Art. 2,3,4; Subs. 2,2,2; Recr 4,4,4; Disc. 4,4,4]
146	Mauritius	2	Boistol, L., Harper, S., Booth, S. and Zeller, D. 2011. Reconstruction of marine fisheries catches for Mauritius and its outer islands, 1950-2008. pp. 39-61. *In:* S. Harper and D. Zeller (eds.) *Fisheries catch reconstructions: Islands, Part II.* Fisheries Centre Research Reports 19(4), University of British Columbia, Vancouver. (http://www.seaaroundus.org/doc/publications/chapters/2011/Boistol-et-al-Mauritius.pdf).<br>[Indust. 2,3,3; Art. 2,3,3; Subs. 2,3,3; Recr. 1,1,1; Disc. 2,3,3]
147	México (Atlantic)	1,4	(1) Cisneros-Montemayor, A.M., Cisneros-Mata, M.A., Harper, S., Pauly, D. 2015. Unreported marine fisheries catch in Mexico, 1950-2010. Fisheries Centre Working Paper #2015-22, University of British Columbia, Vancouver, 9 p. (http://www.seaaroundus.org/doc/publications/wp/2015/Cisneros-et-al-Mexico.pdf).<br>(4) Cisneros-Montemayor, A.M., Cisneros-Mata, M.A., Harper, S., Pauly, D. 2013. Extent and implications of IUU catch in México's marine fisheries. *Marine Policy* 39: 283-288<br>[Indust. 3,3,4; Art. 1,1,2; Subs. 1,1,1; Recr 1,2,2; Disc. -,-,-]
148	México (Pacific)	1,4	(1) Cisneros-Montemayor, A.M., Cisneros-Mata, M.A., Harper, S., Pauly, D. 2015. Unreported marine fisheries catch in Mexico, 1950-2010. Fisheries Centre Working Paper #2015-22, University of British Columbia, Vancouver, 9 p. (http://www.seaaroundus.org/doc/publications/wp/2015/Cisneros-et-al-Mexico.pdf).<br>(4) Cisneros-Montemayor, A.M., Cisneros-Mata, M.A., Harper, S., Pauly, D. 2013. Extent and implications of IUU catch in México's marine fisheries. *Marine Policy* 39: 283-288<br>[Indust. 3,3,4; Art. 1,1,2; Subs. 1,1,1; Recr 1,2,2; Disc. -,-,-]
149	Micronesia (Federated States of)	1,4	(1)Vali, S., Rhodes, K., Au, A., Zylich, K., Harper, S. and Zeller, D. 2014. Reconstruction of total fisheries catches for the Federated States of Micronesia (1950–2010). Fisheries Centre Working Paper #2014-06, University of British Columbia, Vancouver, 16 p. (http://www.seaaroundus.org/doc/publications/wp/2014/Vali-et-al-Federated-States-of-Micronesia.pdf).<br>(4) Zeller, D., Harper, S., Zylich, K. and Pauly, D. 2015. Synthesis of under-reported small-scale fisheries catch in Pacific island waters. *Coral Reefs* 34(1): 25-39.<br>[Indust. 1,2,3; Art. 2,2,3; Subs. 2,2,3; Recr. 1,1,1; Disc. 2,3,3]
150	Montserrat	2	Ramdeen, R., Ponteen, A., Harper, S. and Zeller, D. 2012. Reconstruction of total marine fisheries catches for Montserrat (1950-2010). pp. 69-76. *In*: S. Harper, K. Zylich, L. Boonzaier, F. Le Manach, D. Pauly and D. Zeller (eds.) *Fisheries catch reconstructions: Islands, Part III*. Fisheries Centre Research Reports 20(5), University of British Columbia, Vancouver. (http://www.seaaroundus.org/doc/publications/chapters/2012/Ramdeen-et-al-Montserrat.pdf).<br>[Indust. 1,2,3; Art. 1,2,3; Subs. 1,2,3; Recr. 1,1,1; Disc. 1,1,1]
151	Montenegro	1	Keskin, Ç., Ulman, A., Iritani, D. and Zeller, D. 2014. Reconstruction of fisheries catches for Montenegro: 1950-2010. Fisheries Centre Working Paper #2014-27, University of British Columbia, Vancouver, 11 p. (http://www.seaaroundus.org/doc/publications/wp/2014/Keskin-et-al-Montenegro.pdf).<br>[Indust. 2,3,3; Art. 2,3,3; Subs. 1,1,1; Recr. 1,1,1; Disc. 2,2,2]
152	Morocco (Central)	2,4,4	(2) Belhabib, D., Harper, S., Zeller, D. and Pauly, D. 2012. Reconstruction of marine fisheries catches for Morocco (North, Central and South), 1950-2010. pp. 23-40 *In*: D. Belhabib, D. Zeller, S. Harper and D. Pauly (eds.) *Marine fisheries catches in West Africa, Part I.* Fisheries Centre Research Reports 20(3), University of British Columbia, Vancouver. (http://www.seaaroundus.org/doc/publications/chapters/2012/Belhabib-et-al-Morocco.pdf).<br>(4) Belhabib, D., Sumaila, U.R., Lam, V.W.Y., Zeller, D., Le Billon, P., Kane, E.A. and Pauly, D. 2015. Euro vs. Yuan: Comparing European and Chinese fishing access in West Africa. *PLoS One* 10(3): e0118351<br>(4) Belhabib, D., Sumaila, U.R. and Pauly, D. 2015. Feeding the poor: contribution of West African fisheries to employment and food security. *Ocean & Coastal Management* 111: 72-81.<br>[Indust. 3,3,3; Art. 3,4,4; Subs. 2,2,2; Recr. 2,2,4; Disc. 3,4,4]
153	Morocco (Mediterranean)	2,4,4	(2) Belhabib, D., Harper, S., Zeller, D. and Pauly, D. 2012. Reconstruction of marine fisheries catches for Morocco (North, Central and South), 1950-2010. pp. 23-40 *In*: D. Belhabib, D.

Supplementary Table 5: Sources of reconstructions by country/EEZ component with associated publication links. Publication types are: 1: *Fisheries Centre Working Paper*, 2: *Fisheries Centre Research Reports*, 3: Other reports; 4: Primary literature. Uncertainty scores (as per Table S1) are given below the source of reconstruction for the three time periods 1950-1969, 1970-1989, 1990-2010 in square brackets by fishing sectors: Indust. = industrial, Art. = artisanal, Subs. = subsistence, Recr. = recreational, plus Disc. = discards.

#	Country	Publ. type	Source of reconstruction
			Zeller, S. Harper and D. Pauly (eds.) *Marine fisheries catches in West Africa, Part 1*. Fisheries Centre Research Reports 20(3), University of British Columbia, Vancouver. (http://www.seaaroundus.org/doc/publications/chapters/2012/Belhabib-et-al-Morocco.pdf).<br>(4) Belhabib, D., Sumaila, U.R., Lam, V.W.Y., Zeller, D., Le Billon, P., Kane, E.A. and Pauly, D. 2015. Euro vs. Yuan: Comparing European and Chinese fishing access in West Africa. *PLoS One* 10(3): e0118351<br>(4) Belhabib, D., Sumaila, U.R. and Pauly, D. 2015. Feeding the poor: contribution of West African fisheries to employment and food security. *Ocean & Coastal Management* 111: 72-81.<br>[Indust. 3,3,3; Art. 3,4,4; Subs. 2,2,2; Recr. 2,2,4; Disc. 3,4,4]
154	Morocco (South)	2,4,4	(2) Belhabib, D., Harper, S., Zeller, D. and Pauly, D. 2012. Reconstruction of marine fisheries catches for Morocco (North, Central and South), 1950-2010. pp. 23-40 *In*: D. Belhabib, D. Zeller, S. Harper and D. Pauly (eds.) *Marine fisheries catches in West Africa, Part I.* Fisheries Centre Research Reports 20(3), University of British Columbia, Vancouver. (http://www.seaaroundus.org/doc/publications/chapters/2012/Belhabib-et-al-Morocco.pdf).<br>(4) Belhabib, D., Sumaila, U.R., Lam, V.W.Y., Zeller, D., Le Billon, P., Kane, E.A. and Pauly, D. 2015. Euro vs. Yuan: Comparing European and Chinese fishing access in West Africa. *PLoS One* 10(3): e0118351<br>(4) Belhabib, D., Sumaila, U.R. and Pauly, D. 2015. Feeding the poor: contribution of West African fisheries to employment and food security. *Ocean & Coastal Management* 111: 72-81.<br>[Indust. 3,3,3; Art. 3,4,4; Subs. 2,2,2; Recr. 2,2,4; Disc. 3,4,4]
155	Mozambique	2,4	(2) Doherty, B., McBride, M.M., Brito, A.J., Le Manach, F., Sousa, L., Chauca, I. and Zeller, D. 2015. Marine Fisheries in Mozambique: Catches Updated to 2010 and Taxonomic Disaggregation. pp. 67-82. *In:* F. Le Manach and D. Pauly (eds.) *Fisheries catch reconstructions in the Western Indian Ocean, 1950-2010.* Fisheries Centre Research Report 23(2), University of British Columbia, Vancouver. (http://www.seaaroundus.org/doc/publications/chapters/2015/Doherty-et-al-2015-Mozambique.pdf).<br>(4) Jacquet, J., Fox, H., Motta, H., Ngusaru, A. and Zeller, D. 2010. Few data but many fish: marine small-scale fisheries catches for Mozambique and Tanzania. *African Journal of Marine Science*, 32(2), 197-206.<br>[Indust. 1,2,3; Art. 1,1,2; Subs. 1,1,2; Recr. -,-,-; Disc. 1,1,1]
156	Myanmar	3	Booth, S. and Pauly, D. 2011. Myanmar's marine capture fisheries 1950-2008: Expansion from the coast to the deep waters. pp. 101-134. *In:* S. Harper, D. O'Meara, S. Booth, D. Zeller and D. Pauly (eds). *Fisheries Catches for the Bay of Bengal Large Marine Ecosystem since 1950*. Report to the Bay of Bengal Large Marine Ecosystem Project. BOBLME-2011-Ecology-16.<br>Since completing the initial reconstruction, the 2008 total reconstructed catch was carried forward, unaltered, to 2010. FAO data became available to 2010 and were used for the reported component. The unreported component for 2010 was then taken to be the difference between these two numbers. Some amendments to the FAO dataset were required, hence the reflection of those revisions in the original reconstruction. The sectoral breakdown (artisanal, large-scale etc.) for the reported component of the 2009-2010 estimated catch was based on the same percentage breakdown (of the reported component) used in 2008. Proportions applied to all species were corrected as reported landings in the initial reconstruction did not match the amounts that FAO had reported. "Natantian decapods" were not originally included in the taxonomic breakdown, thus the category was added. "Jellyfish" was not included in the taxonomic breakdown of the initial reconstruction, yet was reported by FAO and consequently were re-allocated. The sectoral breakdown (artisanal, subsistence, large-scale etc.) for the unreported component was based on the same percentage breakdown (of the unreported component) used in 2008. The taxonomic breakdown for the unreported component was also based on the percentage breakdown in 2008 (calculated separately by sector).<br>[Indust. 2,3,3; Art. 2,3,3; Subs. 2,3,3; Recr. 1,1,1; Disc. 2,2,2]
157	Namibia	1,4,4,4	(1) Belhabib, D., Willemse, N.E. and Pauly, D. 2015. A fishery tale: Namibian fisheries between 1950 and 2010. Fisheries Centre Working Paper #2015-65, University of British Columbia, Vancouver, 17 p. (http://www.seaaroundus.org/doc/publications/wp/2015/Belhabib-et-al-Namibia.pdf).<br>(4) Belhabib, D., Sumaila, U.R., Lam, V.W.Y., Zeller, D., Le Billon, P., Kane, E.A. and Pauly, D. 2015. Euro vs. Yuan: Comparing European and Chinese fishing access in West Africa. *PLoS One* 10(3): e0118351<br>(4) Belhabib, D., Sumaila, U.R. and Pauly, D. 2015. Feeding the poor: contribution of West African fisheries to employment and food security. *Ocean & Coastal Management* 111: 72-81.<br>(4) Belhabib, D., Mendy, A., Subah, Y., Broh, N.T., Jueseah, A.S., Nipey, N., Boeh, W.W., Willemse, N., Zeller, D. and Pauly, D. (in press) Fisheries catch under-reporting in The Gambia, Liberia and Namibia, and the three Large Marine Ecosystems which they represent. *Environmental Development.* DOI: 10.1016/j.envdev.2015.08.004

Supplementary Table 5: Sources of reconstructions by country/EEZ component with associated publication links. Publication types are: 1: *Fisheries Centre Working Paper*, 2: *Fisheries Centre Research Reports*, 3: Other reports; 4: Primary literature. Uncertainty scores (as per Table S1) are given below the source of reconstruction for the three time periods 1950-1969, 1970-1989, 1990-2010 in square brackets by fishing sectors: Indust. = industrial, Art. = artisanal, Subs. = subsistence, Recr. = recreational, plus Disc. = discards.

#	Country	Publ. type	Source of reconstruction
			[Indust. 4,4,4; Art. -,-,-; Subs. 2,2,3; Recr. 3,3,3; Disc. 4,4,4]
158	Nauru	2,4	(2) Trujillo, P., Harper, S. and Zeller, D. 2011. Reconstruction of Nauru's fisheries catches: 1950-2008. pp. 63-71. *In:* S. Harper and D. Zeller (eds.) *Fisheries catch reconstructions: Islands, Part II.* Fisheries Centre Research Reports 19(4), University of British Columbia, Vancouver. (http://www.seaaroundus.org/doc/publications/chapters/2011/Trujillo-et-al-2011-Nauru.pdf).<br>(4) Zeller, D., Harper, S., Zylich, K. and Pauly, D. 2015. Synthesis of under-reported small-scale fisheries catch in Pacific island waters. *Coral Reefs* 34(1): 25-39.<br>[Indust. 2,2,2; Art. 2,2,2; Subs. 2,2,2; Recr. 1,1,1; Disc. 1,1,1]
159	Netherlands	1	Gibson, D., Zylich, K. and Zeller, D. 2015. Preliminary reconstruction of total marine fisheries catches for the Netherlands in the North Sea (1950-2010). Fisheries Centre Working Paper #2015-46, University of British Columbia, Vancouver, 15 p. (http://www.seaaroundus.org/doc/publications/wp/2015/Gibson-et-al-Netherlands.pdf).<br>[Indust. 3,3,4; Art. 3,3,4; Subs. 2,1,1; Recr. 1,1,2; Disc. 1,1,2]
160	Netherlands (Aruba)	1	Pauly, D., Ramdeen, S. and Ulman, A. 2015. Reconstruction of total marine catches for Aruba, Southern Caribbean, 1950-2010. Fisheries Centre Working Paper #2015-10, University of British Columbia, Vancouver, 8 p. (http://www.seaaroundus.org/doc/publications/wp/2015/Pauly-et-al-Aruba.pdf).<br>[Indust. 2,2,2; Art. 2,3,4; Subs. 2,2,2; Recr. 2,2,2; Disc. 2,2,2]
161	Netherlands (Bonaire)	1	Lindop, A., Bultel, E., Zylich, K. and Zeller, D. 2015. Reconstructing the former Netherlands Antilles marine catches from 1950 to 2010. Fisheries Centre Working Paper #2015-69, University of British Columbia, Vancouver, 22 p. (http://www.seaaroundus.org/doc/publications/wp/2015/Lindop-et-al-Netherlands-Antilles.pdf).<br>[Indust. 2,2,2; Art. 2,3,4; Subs. 2,2,2; Recr. 2,2,2; Disc. 2,2,2]
162	Netherlands (Curaçao)	1	Lindop, A., Bultel, E., Zylich, K. and Zeller, D. 2015. Reconstructing the former Netherlands Antilles marine catches from 1950 to 2010. Fisheries Centre Working Paper #2015-69, University of British Columbia, Vancouver, 22 p. (http://www.seaaroundus.org/doc/publications/wp/2015/Lindop-et-al-Netherlands-Antilles.pdf).<br>[Indust. 2,2,2; Art. 2,3,4; Subs. 2,2,2; Recr. 2,2,2; Disc. 2,2,2]
163	Netherlands (Saba and Sint Eustatius)	1	Lindop, A., Bultel, E., Zylich, K. and Zeller, D. 2015. Reconstructing the former Netherlands Antilles marine catches from 1950 to 2010. Fisheries Centre Working Paper #2015-69, University of British Columbia, Vancouver, 22 p. (http://www.seaaroundus.org/doc/publications/wp/2015/Lindop-et-al-Netherlands-Antilles.pdf).<br>[Indust. 2,2,2; Art. 2,3,4; Subs. 2,2,2; Recr. 2,2,2; Disc. 2,2,2]
164	Netherlands (Sint Maarten)	1	Lindop, A., Bultel, E., Zylich, K. and Zeller, D. 2015. Reconstructing the former Netherlands Antilles marine catches from 1950 to 2010. Fisheries Centre Working Paper #2015-69, University of British Columbia, Vancouver, 22 p. (http://www.seaaroundus.org/doc/publications/wp/2015/Lindop-et-al-Netherlands-Antilles.pdf).<br>[Indust. 2,2,2; Art. 2,3,4; Subs. 2,2,2; Recr. 2,2,2; Disc. 2,2,2]
165	New Zealand	1	Simmons, G., Bremner, G., Stringer, C., Torkington, B., Teh, L.C.L., Zylich, K., Zeller, D., Pauly, D. and Whittaker, H. 2015. Preliminary reconstruction of marine fisheries catches for New Zealand (1950-2010). Fisheries Centre Working Paper #2015-87, University of British Columbia, Vancouver, 33 p. (http://www.seaaroundus.org/doc/publications/wp/2015/Simmons-et-al-New-Zealand.pdf).<br>[Indust. 2,3,3; Art. 2,3,3; Subs. 2,2,2; Recr. 2,2,3; Disc. 2,3,3]
166	New Zealand (Kermadec Islands)	2	Zylich, K., Harper, S. and Zeller, D. 2012. Reconstruction of marine fisheries catches for the Kermadec Islands (1950-2010). pp. 61-67. *In:* S. Harper, K. Zylich, L. Boonzaier, F. Le Manach, D. Pauly and D. Zeller (eds.) *Fisheries catch reconstructions: Islands, Part III.* Fisheries Centre Research Reports 20(5), University of British Columbia, Vancouver. (http://www.seaaroundus.org/doc/publications/chapters/2012/Zylich-et-al-Kermadec.pdf).<br>[Indust. 1,2,3; Art. -,-,-; Subs. -,-,-; Recr. -,-,-; Disc. 1,2,3]
167	New Zealand (Tokelau)	2,4	(2) Zylich, K., Harper, S. and Zeller, D. 2011. Reconstruction of fisheries catches for Tokelau (1950-2009). pp. 107-117. *In*: S. Harper and D. Zeller (eds.) *Fisheries catch reconstructions: Islands, Part II.* Fisheries Centre Research Reports 19(4), University of British Columbia, Vancouver. (http://www.seaaroundus.org/doc/publications/chapters/2011/Zylich-et-al-2011-Tokelau.pdf).<br>(4) Zeller, D., Harper, S., Zylich, K. and Pauly, D. 2015. Synthesis of under-reported small-scale fisheries catch in Pacific island waters. *Coral Reefs* 34(1): 25-39.<br>[Indust. 2,3,3; Art. 2,3,3; Subs. 2,2,2; Recr. 1,1,1; Disc. 2,2,2]

Supplementary Table 5: Sources of reconstructions by country/EEZ component with associated publication links. Publication types are: 1: *Fisheries Centre Working Paper*, 2: *Fisheries Centre Research Reports*, 3: Other reports; 4: Primary literature. Uncertainty scores (as per Table S1) are given below the source of reconstruction for the three time periods 1950-1969, 1970-1989, 1990-2010 in square brackets by fishing sectors: Indust. = industrial, Art. = artisanal, Subs. = subsistence, Recr. = recreational, plus Disc. = discards.

#	Country	Publ. type	Source of reconstruction
168	Nicaragua (Caribbean)	1	Haas, A., Harper, S. and Zeller, D. 2015. Reconstruction of Nicaragua's fisheries catches: 1950-2010. Fisheries Centre Working Paper #2015-23, University of British Columbia, Vancouver, 9 p. (http://www.seaaroundus.org/doc/publications/wp/2015/Haas-et-al-Nicaragua.pdf).<br>[Indust. 2,2,2; Art. 2,2,2; Subs. 2,1,2; Recr. -,-,-; Disc. 3,2,3]
169	Nicaragua (Pacific)	1	Haas, A., Harper, S. and Zeller, D. 2015. Reconstruction of Nicaragua's fisheries catches: 1950-2010. Fisheries Centre Working Paper #2015-23, University of British Columbia, Vancouver, 9 p. (http://www.seaaroundus.org/doc/publications/wp/2015/Haas-et-al-Nicaragua.pdf).<br>[Indust. 2,2,2; Art. 2,2,2; Subs. 2,1,2; Recr. -,-,-; Disc. 3,2,3]
170	Nigeria	2,4,4	(2) Etim, L., Belhabib, D. and Pauly, D. 2015. An overview of the Nigerian marine fisheries and a re-evaluation of its catch data for the years 1950-2010. pp. 66-76. *In:* D. Belhabib and D. Pauly (eds). *Fisheries catch reconstructions: West Africa, Part II.* Fisheries Centre Research Reports 23(3), University of British Columbia. (http://www.seaaroundus.org/doc/publications/chapters/2015/Etim-Nigeria.pdf)<br>(4) Belhabib, D., Sumaila, U.R., Lam, V.W.Y., Zeller, D., Le Billon, P., Kane, E.A. and Pauly, D. 2015. Euro vs. Yuan: Comparing European and Chinese fishing access in West Africa. *PLoS One* 10(3): e0118351<br>(4) Belhabib, D., Sumaila, U.R. and Pauly, D. 2015. Feeding the poor: contribution of West African fisheries to employment and food security. *Ocean & Coastal Management* 111: 72-81.<br>[Indust. 1,3,3; Art. 1.5,2.5,2.5; Subs. 1,3,3; Recr. -,-,-; Disc. 1,2.7,2.7]
171	Niue	2,4	(2) Zylich, K., Harper, S., Winkler, N. and Zeller, D. 2012. Reconstruction of marine figheries catches for Niue (1950-2010). pp. 77-86. *In:* S. Harper, K. Zylich, L. Boonzaier, F. Le Manach, D. Pauly and D. Zeller (eds.) *Fisheries catch reconstructions: Islands, Part III.* Fisheries Centre Research Reports 20(5), University of British Columbia, Vancouver. (http://www.seaaroundus.org/doc/publications/chapters/2012/Zylich-et-al-2012-Niue.pdf).<br>(4) Zeller, D., Harper, S., Zylich, K. and Pauly, D. 2015. Synthesis of under-reported small-scale fisheries catch in Pacific island waters. *Coral Reefs* 34(1): 25-39.<br>[Indust. 2,3,3; Art. 2,3,3; Subs. 2,3,3; Recr. 1,1,1; Disc. 1,2,3]
172	Norway	1	Nedreaas, K., Iversen, S. and Kuhnle, G. 2015. Preliminary estimates of total removals by the Norwegian marine fisheries, 1950-2010. Fisheries Centre Working Paper #2015-94, University of British Columbia, Vancouver, 15 p. (http://www.seaaroundus.org/doc/publications/wp/2015/Nedreaas-et-al-Norway.pdf).<br>[Indust. 2,3,4; Art. 2,3,4; Subs. 2,2,2; Recr. 1,1,1; Disc. 2,3,3]
173	Norway (Bouvet Island)	2	Padilla, A., Zeller, D. and Pauly, D. 2015.The fish and fisheries of Bouvet Island. *In:* M.L.D. Palomares and D. Pauly (eds.) *Marine Fisheries Catches of Sub- Antarctic Islands, 1950 to 2010.* pp. 20-29. Fisheries Centre Research Reports 23(1), University of British Columbia, Vancouver. (http://www.seaaroundus.org/doc/publications/chapters/2015/Padilla-et-al-2015-Bouvet-Island.pdf)<br>[Indust. 2,4,4; Art. -,-,-; Subs. -,-,-; Recr. -,-,-; Disc. 1,2,2]
174	Norway (Jan Mayen)	1	Nedreaas, K., Iversen, S. and Kuhnle, G. 2015. Preliminary estimates of total removals by the Norwegian marine fisheries, 1950-2010. Fisheries Centre Working Paper #2015-94, University of British Columbia, Vancouver, 15 p. (http://www.seaaroundus.org/doc/publications/wp/2015/Nedreaas-et-al-Norway.pdf).<br>[Indust. 2,3,4; Art. 2,3,4; Subs. 2,2,2; Recr. 1,1,1; Disc. 2,3,3]
175	Norway (Svalbard)	1	Nedreaas, K., Iversen, S. and Kuhnle, G. 2015. Preliminary estimates of total removals by the Norwegian marine fisheries, 1950-2010. Fisheries Centre Working Paper #2015-94, University of British Columbia, Vancouver, 15 p. (http://www.seaaroundus.org/doc/publications/wp/2015/Nedreaas-et-al-Norway.pdf).<br>[Indust. 2,3,3; Art. 2,2,2; Subs. 1,1,1; Recr. 1,1,1; Disc. 2,2,2]
176	Oman	1	Khalfallah, M., Zylich, K., Zeller, D. and Pauly, D. 2015. Reconstruction of marine fisheries catches for Oman (1950-2010). Fisheries Centre Working Paper #2015-89, University of British Columbia, Vancouver, 11 p. (http://www.seaaroundus.org/doc/publications/wp/2015/Khalfallah-et-al-Oman.pdf).<br>[Indust. 2,3,3; Art. 2,3,3; Subs. 2,2,2; Recr. 1,1,1; Disc. 1,2,2]
177	Oman (Musandam Peninsula)	1	Khalfallah, M., Zylich, K., Zeller, D. and Pauly, D. 2015. Reconstruction of marine fisheries catches for Oman (1950-2010). Fisheries Centre Working Paper #2015-89, University of British Columbia, Vancouver, 11 p. (http://www.seaaroundus.org/doc/publications/wp/2015/Khalfallah-et-al-Oman.pdf).<br>[Indust. 1,1,1; Art. 2,2,3; Subs. 1,1,1; Recr. 1,1,1; Disc. 1,1,1]
178	Pakistan	1	Hornby, C., Moazzam, M., Zylich, K. and Zeller, D. 2014. Reconstruction of Pakistan's marine fisheries catches (1950–2010). Fisheries Centre Working Paper #2014-28, University of British Columbia, Vancouver, 54 p. (http://www.seaaroundus.org/doc/publications/wp/2014/Hornby-et-al-Pakistan.pdf).

Supplementary Table 5: Sources of reconstructions by country/EEZ component with associated publication links. Publication types are: 1: *Fisheries Centre Working Paper*, 2: *Fisheries Centre Research Reports*, 3: Other reports; 4: Primary literature. Uncertainty scores (as per Table S1) are given below the source of reconstruction for the three time periods 1950-1969, 1970-1989, 1990-2010 in square brackets by fishing sectors: Indust. = industrial, Art. = artisanal, Subs. = subsistence, Recr. = recreational, plus Disc. = discards.

#	Country	Publ. type	Source of reconstruction
			[Indust. 2,3,3; Art. 2,2,2; Subs. 2,2,2; Recr. 1,1,1; Disc. 2,2,2]
179	Palau	2,4	(2) Lingard, S., Harper, S., Ota, Y. and Zeller, D. 2011. Marine Fisheries of Palau, 1950-2008: Total reconstructed catch. pp. 73-84. *In:* S. Harper and D. Zeller (eds.) *Fisheries catch reconstructions: Islands, Part II.* Fisheries Centre Research Reports 19(4), University of British Columbia, Vancouver. (http://www.seaaroundus.org/doc/publications/chapters/2011/Lingard-et-al-2011-Palau.pdf).<br>(4) Zeller, D., Harper, S., Zylich, K. and Pauly, D. 2015. Synthesis of under-reported small-scale fisheries catch in Pacific island waters. *Coral Reefs* 34(1): 25-39.<br>[Indust. 2,3,3; Art. 3,3,3; Subs. 2,2,2; Recr. 1,1,1; Disc. 2,2,2]
180	Panama (Caribbean)	4	Harper, S., Guzman, H.M., Zylich, K. and Zeller, D. 2014. Reconstructing Panama's total fisheries catches from 1950 to 2010: highlighting data deficiencies and management needs. *Marine Fisheries Review*, 76 (1-2): 51-65. doi: dx.doi.org/10.7755/MFR.76.1_2.3<br>[Indust. 3,3,3; Art. 2,2,2; Subs. 1,1,1; Recr. 1,2,1; Disc. 1,1,2]
181	Panama (Pacific)	4	Harper, S., Guzman, H.M., Zylich, K. and Zeller, D. 2014. Reconstructing Panama's total fisheries catches from 1950 to 2010: highlighting data deficiencies and management needs. *Marine Fisheries Review*, 76 (1-2): 51-65. doi: dx.doi.org/10.7755/MFR.76.1_2.3<br>[Indust. 3,3,3; Art. 2,2,2; Subs. 1,1,1; Recr. 1,2,1; Disc. 1,1,2]
182	Papua New Guinea	1,4	(1) Teh, L.C.L, Kinch, J., Zylich, K. and Zeller, D. 2014. Reconstructing Papua New Guineas's Marine Fisheries Catch, 1950-2010. Fisheries Centre Working Paper #2014-09, University of British Columbia, Vancouver, 23 p. (http://www.seaaroundus.org/doc/publications/wp/2014/Teh-et-al-Papua-New-Guinea.pdf).<br>(4) Zeller, D., Harper, S., Zylich, K. and Pauly, D. 2015. Synthesis of under-reported small-scale fisheries catch in Pacific island waters. *Coral Reefs* 34(1): 25-39.<br>[Indust. 2,2,2; Art. 2,2,2; Subs. 2,2,2; Recr. 1,1,1; Disc. 2,2,2]
183	Peru	1	Mendo, J. and Wosnitza-Mendo, C. 2014. Reconstruction of total marine fisheries catches for Peru: 1950-2010. Fisheries Centre Working Paper #2014-21, University of British Columbia, Vancouver, 23 p. (http://www.seaaroundus.org/doc/publications/wp/2014/Mendo-et-al-Peru.pdf).<br>[Indust. 3,4,4; Art. 3,3,3; Subs. 3,3,3; Recr. 1,2,2; Disc. 3,3,3]
184	Philippines	2	Palomares, M.L.D. and Pauly, D. (eds.) 2014. *Philippine Marine Fisheries Catches: A Bottom-up Reconstruction, 1950 to 2010.* Fisheries Centre Research Report 22(1), University of British Columbia, Vancouver, 171 p. (http://www.fisheries.ubc.ca/webfm_send/365).<br>[Indust. 2,2,2; Art. 3,3,3; Subs. 3,3,3; Recr. 3,3,3; Disc. 2,2,2]
185	Poland	2,4	(2) Bale, S., Rossing, P., Booth, S., Wowkonowicz, P. and Zeller, D. 2010. Poland's fisheries catches in the Baltic Sea (1950-2007). pp. 165-188. *In:* R. Rossing, S. Booth and D. Zeller (eds.) *Total marine fisheries extractions by country in the Baltic Sea: 1950-present.* Fisheries Centre Research Reports 18(1), University of British Columbia, Vancouver. (http://www.seaaroundus.org/doc/publications/chapters/2010/Harper-et-al-Bale-et-al-Poland.pdf).<br>(4) Zeller, D., Rossing, P., Harper, S., Persson, L., Booth, S. and Pauly, D. 2011. The Baltic Sea: estimates of total fisheries removals 1950-2007. *Fisheries Research* 108: 356-363.<br>Since completing the initial reconstruction, ICES landing statistics became available to 2010. To update the reconstruction, ICES landing statistics for 2008-2010 were accepted as the reported landings. The unreported component was calculated using the 2007 IUU rates (by species), which were applied to the reported landings. To calculate discards, the 2007 discard rates (by species) were applied to the sum of reported landings and unreported catches. To calculate recreational catch, population data was first retrieved from Populstat (www.populstat.info), and if needed, a linear interpolation was used to estimate annual population. The 2007 per capita catch rate for the recreational sector was then applied to the 2008-2010 population estimates to calculate total recreational catch for those years. Please note that the values and comparisons for the years 1950-2007 were based on the 2007 ICES dataset, and changes were not made to account for small differences within the 2010 dataset regarding previous years.<br>[Indust. 2,3,2; Art. 2,2,2; Subs. 1,1,1; Recr. 1,1,1; Disc. 2,2,1]
186	Portugal	1,4	(1) Leitão, F., Baptista, V., Erzini, K., Iritani, D. and Zeller, D. 2014. Reconstruction of mainland Portugal fisheries catches 1950-2010. Fisheries Centre Working Paper #2014-08, University of British Columbia, Vancouver, 29 p. (http://www.seaaroundus.org/doc/publications/wp/2014/Leitao-et-al-Portugal.pdf).<br>(4) Leitão, F., Baptista, V., Zeller, D. and Erzini, K. 2014. Reconstructed catches and trends for mainland Portugal fisheries between 1938 and 2009: implications for sustainability, domestic fish supply and imports. *Fisheries Research* 155: 33-50.<br>[Indust. 2,3,4; Art. 2,3,3; Subs. 2,2,2; Recr. 2,2,2; Disc. 2,2,2]
187	Portugal (Azores)	4	Pham, C.K, Canha, A., Diogo, H., Pereira, J.G., Prieto, R. and Morato, T. 2013. Total marine

Supplementary Table 5: Sources of reconstructions by country/EEZ component with associated publication links. Publication types are: 1: *Fisheries Centre Working Paper*, 2: *Fisheries Centre Research Reports*, 3: Other reports; 4: Primary literature. Uncertainty scores (as per Table S1) are given below the source of reconstruction for the three time periods 1950-1969, 1970-1989, 1990-2010 in square brackets by fishing sectors: Indust. = industrial, Art. = artisanal, Subs. = subsistence, Recr. = recreational, plus Disc. = discards.

#	Country	Publ. type	Source of reconstruction
			fishery catch for the Azores (1950-2010). *ICES Journal of Marine Science*. 70(3): 564-577.<br>[Indust. 3,3,4; Art. 3,3,4; Subs. 2,2,3; Recr. 2,3,3; Disc. 1,1,3]
188	Portugal (Madeira)	1	Shon, S., Delgado, J.M., Morato, T., Pham, C.K., Zylich, K., Zeller, D. and Pauly, D. 2015. Reconstruction of marine fisheries catches for Madeira Island, Portugal, from 1950-2010. Fisheries Centre Working Paper #2015-52, University of British Columbia, Vancouver, 13 p. (http://www.seaaroundus.org/doc/publications/wp/2015/Shon-et-al-Madeira.pdf).<br>[Indust. 2,3,4; Art. 2,3,3; Subs. 2,2,2; Recr. 2,2,2; Disc. 2,2,2]
189	Qatar	2,4	(2) Al-Abdulrazzak, D. 2013. Total fishery extractions for Qatar: 1950-2010. pp. 31-37. *In*: D. Al-Abdulrazzak and D. Pauly (eds.) *From dhows to trawlers: a recent history of fisheries in the Gulf countries, 1950 to 2010.* Fisheries Centre Research Reports 21(2), University of British Columbia, Vancouver. (http://www.seaaroundus.org/doc/publications/chapters/2013/AlAbdulrazzak-Qatar.pdf).<br>(4) Al-Abdulrazzak, D., Zeller, D., Belhabib, D., Tesfamichael, D. and Pauly, D. 2015. Total marine fisheries catches in the Persian/Arabian Gulf from 1950-2010. *Regional Studies in Marine Science* 2: 28-34.<br>[Indust. -,3,2; Art. 3,3,3; Subs. 1,1,1; Recr. 1,1,1; Disc. 3,3,2]
190	Romania	1	Bănaru, D., Le Manach, F., Färber, L., Zylich, K. and Pauly, D. 2015. From bluefin tuna to gobies: a reconstruction of the fisheries catch statistics in Romania, 1950-2010. Fisheries Centre Working Paper #2015-48, University of British Columbia, Vancouver, 10 p. (http://www.seaaroundus.org/doc/publications/wp/2015/Banaru-et-al-Romania.pdf).<br>[Indust. 2,3,3; Art. 2,3,3; Subs. 2,2,2; Recr. 1,1,1; Disc. 2,2,2]
191	Russia (Baltic Sea)	2,4	(2) Harper, S., Shibaev, S.V., Baryshnikova, O., Rossing, P., Booth, S. and Zeller, D. 2010. Russian fisheries catches in the Baltic Sea from 1950-2007. pp. 189-224. *In:* R. Rossing, S. Booth and D. Zeller (eds.) *Total marine fisheries extractions by country in the Baltic Sea: 1950-present.* Fisheries Centre Research Reports 18(1), University of British Columbia, Vancouver. (http://www.seaaroundus.org/doc/publications/chapters/2010/Harper-et-al-Russia-Baltic.pdf).<br>(3) Zeller, D., Rossing, P., Harper, S., Persson, L., Booth, S. and Pauly, D. 2011. The Baltic Sea: estimates of total fisheries removals 1950-2007. *Fisheries Research* 108: 356-363.<br>Since completing the initial reconstructions, ICES landing statistics became available to 2010. To update the reconstructions, ICES landing statistics for 2008-2010 were accepted as the reported landings. The unreported components were calculated using the 2007 IUU rates (by species), which were applied to the reported landings. To calculate discards, the 2007 discard rates (by species) were applied to the sum of reported landings and unreported catches. To calculate recreational catches, population data were first retrieved from Populstat (www.populstat.info), and if needed, linear interpolations were used to estimate annual populations. The 2007 per capita catch rates for the recreational sectors were then applied to the 2008-2010 population estimates to calculate total recreational catches for those years. Please note that the values and comparisons for the years 1950-2007 were based on the 2007 ICES dataset, and changes were not made to account for small differences within the 2010 dataset regarding previous years.<br>[Indust. 2,2,2; Art. 2,2,2; Subs. -,-,-; Recr. 1,1,1; Disc. 1,1,1]
192	Russian (Barents Sea)	1	Jovanović, B., Divovich, E., Harper, S., Zeller, D. and Pauly, D. 2015. Estimates of total Russian fisheries catches in the Barents Sea region (FAO 27 subarea I) between 1950 and 2010. Fisheries Centre Working Paper #2015-59, University of British Columbia, Vancouver, 16 p. (http://www.seaaroundus.org/doc/publications/wp/2015/Jovanović-et-al-Russia-Barent-Sea.pdf).<br>[Indust. 3,3,3; Art. 2,2,2; Subs. 1,1,1; Recr. 1,1,1; Disc. 2,2,2]
193	Russia (Black and Azov Seas)	1	Divovich, E., Jovanović, B., Zylich, K., Harper, S., Zeller, D. and Pauly, D. 2015. Caviar and politics: A reconstruction of Russia's marine fisheries in the Black Sea and Sea of Azov from 1950 to 2010. Fisheries Centre Working Paper #2015-84, University of British Columbia, Vancouver, 24 p. (http://www.seaaroundus.org/doc/publications/wp/2015/Divovich-et-al-Russia-Black-Sea.pdf).<br>[Indust. 2,3,2; Art. 2,3,2; Subs. 2,2,2; Recr. 1,1,2; Disc. 2,2,3]
194	Russia (Far East)	1	Sobolevskaya, A. and Divovich, E. 2015. The Wall Street of fisheries: the Russian Far East, a catch reconstruction from 1950-2010. Fisheries Centre Working Paper #2015-45, University of British Columbia, Vancouver, 65 p.(http://www.seaaroundus.org/doc/publications/wp/2015/Sobolevskaya-and-Divovich-Russia-Far-East.pdf).<br>[Indust. 4,4,4; Art. 4,4,4; Subs. 2,3,3; Recr. 2,3,3; Disc. 1,1,3]
195	Russia (Kara Sea)	2,2,4	(2) Pauly, D. and Swartz, W. 2007. Marine fish catches in North Siberia (Russia, FAO Area 18). pp. 17-33 *In:* D. Zeller and D. Pauly (eds.) *Reconstruction of Marine Fisheries Catches for Key Countries and Regions (1950-2005).* Fisheries Centre Research Report 15(2),

Supplementary Table 5: Sources of reconstructions by country/EEZ component with associated publication links. Publication types are: 1: *Fisheries Centre Working Paper*, 2: *Fisheries Centre Research Reports*, 3: Other reports; 4: Primary literature. Uncertainty scores (as per Table S1) are given below the source of reconstruction for the three time periods 1950-1969, 1970-1989, 1990-2010 in square brackets by fishing sectors: Indust. = industrial, Art. = artisanal, Subs. = subsistence, Recr. = recreational, plus Disc. = discards.

#	Country	Publ. type	Source of reconstruction
			University of British Columbia, Vancouver. (http://www.seaaroundus.org/doc/publications/chapters/2007/Pauly-and-Swartz-North-Siberia.pdf).<br>(2) Teh, L.C.L., Zylich, K. and Zeller, D. 2015. FAO area 18 (Arctic Sea): Catch data reconstruction extension of Zeller *et al.* (2011) to 2010. Fisheries Centre Working Paper #2015-14, University of British Columbia, Vancouver, 5 p. (http://www.seaaroundus.org/doc/publications/wp/2015/Teh-et-al-Arctic-Sea.pdf).<br>(4) Zeller, D., Booth, S., Pakhomov, E., Swartz, W. and Pauly, D. 2011. Arctic fisheries catches in Russia, USA and Canada: Baselines for neglected ecosystems. *Polar Biology* 34(7): 955-973.<br>[Indust. 1,1,1; Art. 1,1,1; Subs. 1,1,1; Recr. 1,1,1; Disc. 1,1,1]
196	Russia (Laptev to Chukchi Seas)	1,2,4	(1) Teh, L.C.L., Zylich, K. and Zeller, D. 2015. FAO area 18 (Arctic Sea): Catch data reconstruction extension of Zeller *et al.* (2011) to 2010. Fisheries Centre Working Paper #2015-14, University of British Columbia, Vancouver, 5 p. (http://www.seaaroundus.org/doc/publications/wp/2015/Teh-et-al-Arctic-Sea.pdf).<br>(2) Pauly, D. and Swartz, W. 2007. Marine fish catches in North Siberia (Russia, FAO Area 18). pp. 17-33 *In:* D. Zeller and D. Pauly (eds.) *Reconstruction of Marine Fisheries Catches for Key Countries and Regions (1950-2005)*. Fisheries Centre Research Reports, 15(2). (http://www.seaaroundus.org/doc/publications/chapters/2007/Pauly-and-Swartz-North-Siberia.pdf).<br>(4) Zeller, D., Booth, S., Pakhomov, E., Swartz, W. and Pauly, D. 2011. Arctic fisheries catches in Russia, USA and Canada: Baselines for neglected ecosystems. *Polar Biology* 34(7): 955-973.<br>[Indust. 1,1,1; Art. 1,1,1; Subs. 1,1,1; Recr. 1,1,1; Disc. 1,1,1]
197	Samoa	2,4	(2) Lingard, S., Harper, S. and Zeller, D. 2012. Reconstructed catches of Samoa 1950-2010, pp. 103-118. *In:* S. Harper, K. Zylich, L. Boonzaier, F. Le Manach, D. Pauly and D. Zeller (eds.) *Fisheries catch reconstructions: Islands, Part III.* Fisheries Centre Research Reports 20(5), University of British Columbia, Vancouver. (http://www.seaaroundus.org/doc/publications/chapters/2012/Lingard-et-al-2012-Samoa.pdf).<br>(4) Zeller, D., Harper, S., Zylich, K. and Pauly, D. 2015. Synthesis of under-reported small-scale fisheries catch in Pacific island waters. *Coral Reefs* 34(1): 25-39.<br>[Indust. 2,3,3; Art. 2,3,3; Subs. 2,3,3; Recr. 1,1,1; Disc. 2,2,2]
198	São Tomé and Príncipe	1,4,4	(1) Belhabib, D. 2015. Fisheries of São Tomé and Príncipe, a catch reconstruction 1950-2010. Fisheries Centre Working Paper #2015-67, University of British Columbia, Vancouver, 13 p. (http://www.seaaroundus.org/doc/publications/wp/2015/Belhabib-Sao-Tome-and-Principe.pdf).<br>(4) Belhabib, D., Sumaila, U.R., Lam, V.W.Y., Zeller, D., Le Billon, P., Kane, E.A. and Pauly, D. 2015. Euro vs. Yuan: Comparing European and Chinese fishing access in West Africa. *PLoS One* 10(3): e0118351<br>(4) Belhabib, D., Sumaila, U.R. and Pauly, D. 2015. Feeding the poor: contribution of West African fisheries to employment and food security. *Ocean & Coastal Management* 111: 72-81.<br>[Indust. 4,4,4; Art. 2,3,3; Subs. 2,3,3; Recr. -,-,-; Disc. -,-,-]
199	Saudi Arabia (Persian Gulf)	2,4	(2) Tesfamichael, D. and Pauly, D. 2013. Catch reconstruction of the fisheries of Saudi Arabia in the Gulf, 1950-2010. pp. 39-52. *In*: D. Al-Abdulrazzak and D. Pauly (eds.) *From dhows to trawlers: a recent history of fisheries in the Gulf countries, 1950 to 2010*. Fisheries Centre Research Reports 21(2), University of British Columbia, Vancouver. (http://www.seaaroundus.org/doc/publications/chapters/2013/Tesfamichael-and-Pauly-Saudi-Arabia-Persian-Gulf.pdf).<br>(4) Al-Abdulrazzak, D., Zeller, D., Belhabib, D., Tesfamichael, D. and Pauly, D. 2015. Total marine fisheries catches in the Persian/Arabian Gulf from 1950-2010. *Regional Studies in Marine Science* 2: 28-34.<br>[Indust. 2,3,3; Art. 2,2,2; Subs. 1,1,1; Recr. 2,2,2; Disc. 2,2,2]
200	Saudi Arabia (Red Sea)	2	Tesfamichael, D. and Rossing, P. 2012. Reconstructing Red Sea fisheries catches of Saudi Arabia: National wealth and fisheries transformation. *In:* D. Tesfamichael and D. Pauly (eds.) *Catch reconstruction for the Red Sea large marine ecosystem by countries (1950–2010).* Fisheries Centre Research Reports 20(1), University of British Columbia, Vancouver. (http://www.seaaroundus.org/doc/publications/chapters/2012/Tesfamichael-and-Rossing-Saudi-Arabia.pdf).<br>[Indust. 2,3,3; Art. 2,2,2; Subs. 1,1,1; Recr. 2,2,2; Disc. 2,2,2]
201	Senegal	1,4,4,4,4	(1) Belhabib, D., Koutob, V., Gueye, N., Mbaye, L., Mathews, C., Lam, V.W.Y., Pauly, D. 2013. Lots of boats and fewer fishes: A preliminary catch reconstruction for Senegal, 1950-2010. Fisheries Centre Working Papers #2013-03, Fisheries Centre, University of British Columbia, Vancouver. 34 p.

Supplementary Table 5: Sources of reconstructions by country/EEZ component with associated publication links. Publication types are: 1: *Fisheries Centre Working Paper*, 2: *Fisheries Centre Research Reports*, 3: Other reports; 4: Primary literature. Uncertainty scores (as per Table S1) are given below the source of reconstruction for the three time periods 1950-1969, 1970-1989, 1990-2010 in square brackets by fishing sectors: Indust. = industrial, Art. = artisanal, Subs. = subsistence, Recr. = recreational, plus Disc. = discards.

#	Country	Publ. type	Source of reconstruction
			(http://www.seaaroundus.org/doc/publications/wp/2014/Belhabib-et-al-Senegal.pdf).<br>(4) Belhabib, D., Koutob, V., Sall, A., Lam, V.W.Y. and Pauly, D. 2014. Fisheries catch misreporting and its implications: The case of Senegal. *Fisheries Research* 151:1-11. doi: 10.1016/j.fishres.2013.12.006<br>(4) Belhabib, D., Koutob, V., Sall, A., Lam, V.W.Y. and Pauly, D. 2015. Counting pirogues and missing the boat: Reply to Chaboud *et al*.'s comment on Belhabib *et al*. "Fisheries catch misreporting and its implications: The case of Senegal". *Fisheries Research* 164: 325-328.<br>(4) Belhabib, D., Sumaila, U.R., Lam, V.W.Y., Zeller, D., Le Billon, P., Kane, E.A. and Pauly, D. 2015. Euro vs. Yuan: Comparing European and Chinese fishing access in West Africa. *PLoS One* 10(3): e0118351<br>(4) Belhabib, D., Sumaila, U.R. and Pauly, D. 2015. Feeding the poor: contribution of West African fisheries to employment and food security. *Ocean & Coastal Management* 111: 72-81.<br>[Indust. 3,3,4; Art. 2,2,3; Subs. 3,3,3; Recr. 2,2,4; Disc. 4,4,4]
202	Seychelles	2	Le Manach, F., Bach, P., Boistol, L., Robinson, J. and Pauly, D. 2015. Artisanal Fisheries in the World's Second Largest Tuna Fishing Ground - Reconstruction of the Seychelles' Marine Fisheries Catch, 1950–2010. p. 99-109. *In:* F. Le Manach and D. Pauly (eds.) *Fisheries catch reconstructions in the Western Indian Ocean, 1950-2010.* Fisheries Centre Research Report 23(2), University of British Columbia, Vancouver. (http://www.seaaroundus.org/doc/publications/chapters/2015/LeManach-et-al-2015-Seychelles.pdf).<br>[Indust. 2,3,3; Art. 2,3,3; Subs. 1,1,1; Recr. 1,1,1; Disc. 2,2,2]
203	Sierra Leone	1,4,4	(1) Seto, K., Belhabib, D., Copeland, D., Vakily, M., Seilert, H., Sankoh, S., Baio, A., Turay, I., Harper, S., Zeller, D., Zylich, K. and Pauly, D. 2015. Colonialism, conflict, and fish: a reconstruction of marine fisheries catches for Sierra Leone, 1950-2010. Fisheries Centre Working Paper #2015-74, University of British Columbia, Vancouver, 23 p. (http://www.seaaroundus.org/doc/publications/wp/2015/Seto-et-al-Sierra-Leone.pdf).<br>(4) Belhabib, D., Sumaila, U.R., Lam, V.W.Y., Zeller, D., Le Billon, P., Kane, E.A. and Pauly, D. 2015. Euro vs. Yuan: Comparing European and Chinese fishing access in West Africa. *PLoS One* 10(3): e0118351<br>(4) Belhabib, D., Sumaila, U.R. and Pauly, D. 2015. Feeding the poor: contribution of West African fisheries to employment and food security. *Ocean & Coastal Management* 111: 72-81.<br>[Indust. 4,4,4; Art. 2,3,4; Subs. 1,1,1; Recr. -,-,-; Disc. 2,2,4]
204	Singapore	2	Corpus, L. 2014. Reconstructing Singapore's marine fisheries catch, 1950-2010. pp. 137-146. *In*: K. Zylich, D. Zeller, M. Ang and D. Pauly (eds.) *Fisheries catch reconstructions: Islands, Part IV*. Fisheries Centre Research Reports 22(2), University of British Columbia, Vancouver. (http://www.seaaroundus.org/doc/publications/chapters/2014/Corpus-Singapore.pdf).<br>[Indust. 4,4,4; Art. 4,3,3; Subs. 4,3,2; Recr. 3,3,4; Disc. 4,3,3]
205	Slovenia	1	Bolje, A., Marčeta, B., Blejec, A. and Lindop, A. 2015. Marine fish catches in Slovenia between 1950 and 2010. Fisheries Centre Working Paper #2015-58, University of British Columbia, Vancouver, 13 p. (http://www.seaaroundus.org/doc/publications/wp/2015/Bolje-et-al-Slovenia.pdf).<br>[Indust. 2,3,3; Art. 2,3,3; Subs. 1,1,1; Recr. 1,1,1; Disc. 2,2,2]
206	Solomon Islands	2,4	(2) Doyle, B., Harper, S., Jacquet, J. and Zeller, D. 2012. Reconstructing marine fisheries catches in the Solomon Islands: 1950-2009, pp. 119-134. *In:* S. Harper, K. Zylich, L. Boonzaier, F. Le Manach, D. Pauly and D. Zeller (eds.) *Fisheries catch reconstructions: Islands, Part III.* Fisheries Centre Research Reports 20(5), University of British Columbia, Vancouver. (http://www.seaaroundus.org/doc/publications/chapters/2012/Doyle-et-al-2012-Solomon-Islands.pdf).<br>(4) Zeller, D., Harper, S., Zylich, K. and Pauly, D. 2015. Synthesis of under-reported small-scale fisheries catch in Pacific island waters. *Coral Reefs* 34(1): 25-39.<br>[Including on p. 129 an addendum which updates the dataset to 2010]<br>[Indust. -,2,2; Art. 1,1,1; Subs. -,1,1; Recr. -,-,-; Disc. 2,1,1]
207	Somalia	1,2	(1) Persson, L., Lindop, A., Harper, S., Zylich, K. and Zeller, D. 2014. Failed state: Reconstruction of domestic fisheries catches in Somalia 1950-2010. Fisheries Centre Working Paper #2014-10, University of British Columbia, Vancouver, 10 p. (http://www.seaaroundus.org/doc/publications/wp/2014/Persson-et-al-Somalia.pdf).<br>(2) Persson, L., Lindop, A., Harper, S., Zylich, K. and Zeller, D. 2015. Failed state: Reconstruction of domestic fisheries catches in Somalia 1950-2010. p. 111-127. *In:* F. Le Manach and D. Pauly (eds.) *Fisheries catch reconstructions in the Western Indian Ocean, 1950-2010.* Fisheries Centre Research Report 23(2), University of British Columbia, Vancouver. (http://www.seaaroundus.org/doc/publications/chapters/2015/Persson-et-al-2015-Somalia.pdf).<br>[Indust. 1,2,2; Art. 1,2,2; Subs. 1,1,1; Recr. 1,1,1; Disc. 1,1,1]

Supplementary Table 5: Sources of reconstructions by country/EEZ component with associated publication links. Publication types are: 1: *Fisheries Centre Working Paper*, 2: *Fisheries Centre Research Reports*, 3: Other reports; 4: Primary literature. Uncertainty scores (as per Table S1) are given below the source of reconstruction for the three time periods 1950-1969, 1970-1989, 1990-2010 in square brackets by fishing sectors: Indust. = industrial, Art. = artisanal, Subs. = subsistence, Recr. = recreational, plus Disc. = discards.

#	Country	Publ. type	Source of reconstruction
208	South Africa (Atlantic Coast)	2	Baust, S., Teh, L.C.L., Harper, S. and Zeller, D. 2015. South Africa's Marine Fisheries Catches (1950–2010). pp. 129-150. *In:* F. Le Manach and D. Pauly (eds.) *Fisheries catch reconstructions in the Western Indian Ocean, 1950-2010.* Fisheries Centre Research Report 23(2), University of British Columbia, Vancouver. (http://www.seaaroundus.org/doc/publications/chapters/2015/Baust-et-al-2015-South-Africa.pdf).<br>[Indust. 2,2,2; Art. 2,2,2; Subs. 2,2,3; Recr. 2,2,3; Disc. 1,2,2]
209	South Africa (Indian Ocean Coast)	2	Baust, S., Teh, L.C.L., Harper, S. and Zeller, D. 2015. South Africa's Marine Fisheries Catches (1950–2010). pp. 129-150. *In:* F. Le Manach and D. Pauly (eds.) *Fisheries catch reconstructions in the Western Indian Ocean, 1950-2010.* Fisheries Centre Research Report 23(2), University of British Columbia, Vancouver. (http://www.seaaroundus.org/doc/publications/chapters/2015/Baust-et-al-2015-South-Africa.pdf).<br>[Indust. 2,2,2; Art. 2,2,2; Subs. 2,2,3; Recr. 2,2,3; Disc. 1,2,2]
210	South Africa (Prince Edward Islands)	2	Boonzaier, L., Harper, S., Zeller, D. and Pauly, D. 2012. A brief history of fishing in the Prince Edward Islands, South Africa, 1950-2010, pp. 95-101 *In:* S. Harper, K. Zylich, L. Boonzaier, F. Le Manach, D. Pauly and D. Zeller (eds.) *Fisheries catch reconstructions: Islands, Part III.* Fisheries Centre Research Reports 20(5), University of British Columbia, Vancouver. (http://www.seaaroundus.org/doc/publications/chapters/2012/Boonzaier-et-al-Prince-Edward-Is.pdf).<br>[Indust. -,-,4; Art. -,-,-; Subs. -,-,-; Recr. -,-,-; Disc. -,-,3]
211	Spain (Balearic Islands)	1,3	(1) Carreras, M., Coll, M., Quetglas, A., Goñi, R., Pastor, X., Cornax, M.J., Iglesias, M., Massutí, E., Oliver, P., Aguilar, R. and Pauly, D. 2015. Estimates of total fisheries removal for the Balearic Islands (1950-2010). Fisheries Centre Working Paper #2015-19, University of British Columbia, Vancouver, 45 p. (http://www.seaaroundus.org/doc/publications/wp/2015/Carreras-et-al-Balearic-Islands.pdf).<br>(3) Carreras, M. 2014. Evolucion de la pesca Beleares, Oceana, Madrid, 32 p.<br>[Indust. 2,2,4; Art. 2,3,3; Subs. 2,2,3; Recr. 2,2,3; Disc. 2,3,4]
212	Spain (Canary Islands)	1	Castro, J.J., Divovich, E., Delgado de Molina Acevedo, A. and Barrera-Luján, A. 2015. Over-looked and under-reported: A catch reconstruction of marine fisheries in the Canary Islands, Spain, 1950-2010. Fisheries Centre Working Paper #2015-26, University of British Columbia, Vancouver, 35 p. (http://www.seaaroundus.org/doc/publications/wp/2015/Castro-et-al-CanaryIs.pdf).<br>[Indust. -,-,-; Art. 3,3,4; Subs. 2,1,2; Recr. 2,2,3; Disc. 2,2,3]
213	Spain (Mediterranean and Gulf of Cadiz)	1,4	(1) Coll, M., Carreras, M., Cornax, M.J., Massutí, E., Morote, E., Pastor, X., Quetglas, A., Sáez, R., Silva, L., Sobrino, I., Torres, M.A., Tudela, S., Harper, S., Zeller, D. and Pauly, D. 2015. An estimate of the total catch in the Spanish Mediterranean Sea and Gulf of Cadiz regions (1950-2010). Fisheries Centre Working Paper #2015-60, University of British Columbia, Vancouver, 52 p. (http://www.seaaroundus.org/doc/publications/wp/2015/Coll-et-al-Spain-Med-and-Gulf-of-Cadiz.pdf).<br>(4) Pauly, D., Ulman, A., Piroddi, C., Bultel, E. and Coll, M. 2014. 'Reported' versus 'likely' fisheries catches of four Mediterranean countries. pp. 11-17. *In*: J. Lleonart and F. Maynou (eds.) The Ecosystem approach to fisheries in the Mediterranean and Black Seas. *Scientia Marina*. 78S1.<br>[Indust. 3,3,3; Art. 3,3,3; Subs. 1,1,1; Recr. 1,1,1; Disc. 2,2,2]
214	Spain (Northwest)	1	Villasante, S., Macho, G., Giráldez, J., Rivero Rodriguez, S., Isusu de Rivero, J., Harper, S., Zeller, D. and Pauly, D. 2015. Estimates of total fisheries removals from the Northwest of Spain (1950-2010). Fisheries Centre Working Paper #2015-51, University of British Columbia, Vancouver, 21 p. (http://www.seaaroundus.org/doc/publications/wp/2015/Villasante-et-al-Spain-NW.pdf).<br>[Indust. 1,1,2; Art. 1,1,2; Subs. 1,1,2; Recr. 1,1,2; Disc. 1,1,3]
215	Sri Lanka	2	O'Meara, D., Harper, S., Perera, N. and Zeller, D. 2011. Reconstruction of Sri Lanka's fisheries catches: 1950-2008, pp. 85-96. *In:* S. Harper and D. Zeller (eds.) *Fisheries catch reconstructions: Islands, Part II.* Fisheries Centre Research Reports 19(4), University of British Columbia, Vancouver. (http://www.seaaroundus.org/doc/publications/chapters/2011/OMeara-et-al-Sri-Lanka.pdf).<br>Since completing the initial reconstruction, FAO data became available to 2010. To update the reconstruction, the total reconstructed catch was carried forward to 2010. The FAO data were used for the reported component, and the sectoral breakdown was based on species. In the initial reconstruction the following industrial reported tonnages were allocated to outside the EEZ: 90% of pelagics, 90% of sea cucumbers, and 80% of some of the sharks. Thus, the same pattern was followed for 2009. However, due to large increases in the reported pelagics in 2010, the 2009

Supplementary Table 5: Sources of reconstructions by country/EEZ component with associated publication links. Publication types are: 1: *Fisheries Centre Working Paper*, 2: *Fisheries Centre Research Reports*, 3: Other reports; 4: Primary literature. Uncertainty scores (as per Table S1) are given below the source of reconstruction for the three time periods 1950-1969, 1970-1989, 1990-2010 in square brackets by fishing sectors: Indust. = industrial, Art. = artisanal, Subs. = subsistence, Recr. = recreational, plus Disc. = discards.

#	Country	Publ. type	Source of reconstruction
			tonnages allocated to inside the EEZ were flatlined to 2010, and the remainder were allocated to outside the EEZ. For the unreported components, the same discard rates were applied to the reported shrimp catches, and the ratio between the FAO reported component and IUU component for 2008 (from inside the EEZ) was calculated and applied to the FAO data for 2009 to 2010 to estimate the total reconstructed amounts for those years. The difference was then assigned as IUU from the subsistence sector. The taxonomic breakdowns for both the reported and unreported components were based on the percentage breakdowns in 2008 (calculated separately by sector and input).<br>[Indust. 1,2,2; Art. 1,2,2; Subs. 1,1,1; Recr. 1,1,1; Disc. 1,1,1]
216	St. Kitts and Nevis	2	Ramdeen, R., Zylich, K. and Zeller, D. 2014. Reconstruction of total marine fisheries catches for St. Kitts and Nevis (1950-2010). pp. 129-136. *In*: K. Zylich, D. Zeller, M. Ang and D. Pauly (eds.) *Fisheries catch reconstructions: Islands, Part IV*. Fisheries Centre Research Reports 22(2), University of British Columbia. (http://www.seaaroundus.org/doc/publications/chapters/2014//Ramdeen-et-al-SKN.pdf).<br>[Indust. 2,3,3; Art. 2,3,3; Subs. 2,2,2; Recr. 1,1,1; Disc. 1,1,1]
217	St. Lucia	1	Mohammed, E., Lindop, A. and Joseph, W. 2015. St Lucia, reconstructed fisheries catches, 1950-2010. Fisheries Centre Working Paper #2015-53, University of British Columbia, Vancouver, 25 p. (http://www.seaaroundus.org/doc/publications/wp/2015/Mohammed-et-al-St-Lucia.pdf).<br>[Indust. 2,3,3; Art. 2,3,3; Subs. 2,3,3; Recr. 1,1,1; Disc. 1,1,1]
218	St Vincent and the Grenadines	1	Mohammed, E. and Lindop, A. 2015. St. Vincent and the Grenadines: Reconstructed Fisheries Catches, 1950-2010. Fisheries Centre Working Paper #2015-54, University of British Columbia, Vancouver, 27 p. (http://www.seaaroundus.org/doc/publications/wp/2015/Mohammed-et-al-St-Vincent-and-the-Grenadines.pdf).<br>[Indust. 3,3,3; Art. 3,3,3; Subs. 2,2,2; Recr. 2,2,2; Disc. 2,2,2]
219	Sudan	2	Tesfamichael, D. and Ekawad, A.N. 2012. Reconstructing Red Sea fisheries of Sudan: foreign aid and fisheries. pp. 51-70. *In*: D. Tesfamichael and D. Pauly (eds.) *Catch reconstruction for the Red Sea large marine ecosystem by countries (1950-2010).* Fisheries Centre Research Reports 20(1), University of British Columbia, Vancouver. (http://www.seaaroundus.org/doc/publications/chapters/2012/Tesfamichael-and-Elawad-Sudan.pdf).<br>[Indust. 2,3,3; Art. 2,3,3; Subs. 1,1,1; Recr. 1,1,1; Disc. 1,1,1]
220	Suriname	1	Hornby, C., Harper, S., MacDonald, J. and Zeller, D. 2015. Reconstruction of Suriname's marine fisheries catches from 1950-2010. Fisheries Centre Working Paper #2015-49, University of British Columbia, Vancouver, 14 p. (http://www.seaaroundus.org/doc/publications/wp/2015/Hornby-et-al-Suriname.pdf).<br>[Indust. 2,3,3; Art. 2,3,3; Subs. 1,1,1; Recr. 1,1,1; Disc. 1,1,1]
221	Sweden (Baltic)	2,4	(2) Persson, L. 2010. Sweden's fisheries catches in the Baltic Sea (1950-2007). pp. 225-263. *In:* R. Rossing, S. Booth and D. Zeller (eds.) *Total marine fisheries extractions by country in the Baltic Sea: 1950-present.* Fisheries Centre Research Reports 18(1), University of British Columbia, Vancouver. (http://www.seaaroundus.org/doc/publications/chapters/2010/Persson-Sweden-Baltic.pdf).<br>(3) Zeller, D., Rossing, P., Harper, S., Persson, L., Booth, S. and Pauly, D. 2011. The Baltic Sea: estimates of total fisheries removals 1950-2007. *Fisheries Research* 108: 356-363.<br>Since completing the initial reconstructions, ICES landing statistics became available to 2010. To update the reconstructions, ICES landing statistics for 2008-2010 were accepted as the reported landings. The unreported components were calculated using the 2007 IUU rates (by species), which were applied to the reported landings. To calculate discards, the 2007 discard rates (by species) were applied to the sum of reported landings and unreported catches. To calculate recreational catches, population data were first retrieved from Populstat (www.populstat.info), and if needed, linear interpolations were used to estimate annual populations. The 2007 per capita catch rates for the recreational sectors were then applied to the 2008-2010 population estimates to calculate total recreational catches for those years. Please note that the values and comparisons for the years 1950-2007 were based on the 2007 ICES dataset, and changes were not made to account for small differences within the 2010 dataset regarding previous years.<br>[Indust. 2,3,3; Art. 2,3,3; Subs. 2,2,2; Recr. 2,2,2; Disc. 2,2,2]
222	Sweden (West Coast)	1	Persson, L. 2014. Reconstructing total Swedish catches on the west coast of Sweden: 1950-2010. Fisheries Centre Working Paper #2015-24, University of British Columbia, Vancouver, 10 p. (http://www.seaaroundus.org/doc/publications/wp/2015/Persson-Sweden.pdf).<br>[Indust. 2,3,3; Art. 2,3,3; Subs. 2,2,2; Recr. 2,2,2; Disc. 2,2,2]
223	Syria	1,4	(1) Ulman, A., Saad, A., Zylich, K., Pauly, D. and Zeller, D. 2015. Reconstruction of Syria's fisheries catches from 1950-2010: Signs of overexploitation. Fisheries Centre Working Paper #2015-80, University of British Columbia, Vancouver, 26 p.

Supplementary Table 5: Sources of reconstructions by country/EEZ component with associated publication links. Publication types are: 1: *Fisheries Centre Working Paper*, 2: *Fisheries Centre Research Reports*, 3: Other reports; 4: Primary literature. Uncertainty scores (as per Table S1) are given below the source of reconstruction for the three time periods 1950-1969, 1970-1989, 1990-2010 in square brackets by fishing sectors: Indust. = industrial, Art. = artisanal, Subs. = subsistence, Recr. = recreational, plus Disc. = discards.

#	Country	Publ. type	Source of reconstruction
			(http://www.seaaroundus.org/doc/publications/wp/2015/Ulman-et-al-Syria.pdf).<br>(4) Ulman, A., Saad, A., Zylich, K., Pauly, D. and Zeller, D.. 2015. Reconstruction of Syria's fisheries catches from 1950-2010: Signs of overexploitation. *Acta Ichthyologica et Piscatoria* 45(3): 259-272.<br>[Indust. 1.5,2.5,2.5; Art. 1,3,4; Subs. 1,1,1; Recr. 1,1,1; Disc. -,-,-]
224	Taiwan	1,2	(1) Divovich, E., Färber, L., Shon, S. and Zylich, K. 2015. An updated catch reconstruction of the marine fisheries of Taiwan from 1950-2010. Fisheries Centre Working Paper #2015-78, University of British Columbia, Vancouver, 7 p. (http://www.seaaroundus.org/doc/publications/wp/2015/Divovich-et-al-Taiwan.pdf).<br>(2) Kuo, D. and Booth, S. 2011. From local to global: a catch reconstruction of Taiwan's fisheries from 1950-2007. pp. 97-106. *In:* S. Harper and D. Zeller (eds.) *Fisheries catch reconstructions: Islands, Part II.* Fisheries Centre Research Reports 19(4), University of British Columbia, Vancouver. (http://www.seaaroundus.org/doc/publications/chapters/2011/Kuo-and-Booth-Taiwan.pdf).<br>[Indust. 2,3,3; Art. 2,3,3; Subs. 1,2,2; Recr. 1,1,1; Disc. 2,2,2]
225	Tanzania	2,2	(2) Jacquet, J. and Zeller, D. 2007. Putting the 'United' in the United Republic of Tanzania: Reconstructing marine fisheries catches. pp. 49-60. *In*: D. Zeller and D. Pauly (eds.) *Reconstruction of marine fisheries catches for key countries and regions (1950-2005).* Fisheries Centre Research Reports 15(2), University of British Columbia, Vancouver. (http://www.seaaroundus.org/doc/publications/chapters/2007/Jacquet-and-Zeller-Tanzania.pdf).<br>(2) Bultel, E., Doherty, B., Herman, A., Le Manach, F. and Zeller, D. 2015. An Update of the Reconstructed Marine Fisheries Catches of Tanzania with Taxonomic Breakdown. pp. 151-161. *In:* F. Le Manach and D. Pauly (eds.) *Fisheries catch reconstructions in the Western Indian Ocean, 1950-2010.* Fisheries Centre Research Report 23(2), University of British Columbia, Vancouver. (http://www.seaaroundus.org/doc/publications/chapters/2015/Bultel-et-al-2015-Tanzania.pdf).<br>[Indust. 2,3,3; Art. 2,3,3; Subs. 1,2,2; Recr. 1,2,2; Disc. 2,2,2]
226	Thailand (Andaman Sea)	1	Teh, L.C.L., Zeller, D. and Pauly, D. 2015. Preliminary reconstruction of Thailand's marine fisheries catches: 1950-2010. Fisheries Centre Working Paper #2015-01, University of British Columbia, Vancouver, 14 p. (http://www.seaaroundus.org/doc/publications/wp/2015/Teh-et-al-Thailand.pdf).<br>[Indust. -,2,3; Art. 2,2,3; Subs. 2,2,3; Recr. 1,1,1; Disc. 2,2,3]
227	Thailand (Gulf of Thailand)	1	Teh, L.C.L., Zeller, D. and Pauly, D. 2015. Preliminary reconstruction of Thailand's marine fisheries catches: 1950-2010. Fisheries Centre Working Paper #2015-01, University of British Columbia, Vancouver, 14 p. (http://www.seaaroundus.org/doc/publications/wp/2015/Teh-et-al-Thailand.pdf).<br>[Indust. -,2,3; Art. 2,2,3; Subs. 2,2,3; Recr. 1,1,1; Disc. 2,2,3]
228	Timor-Leste	2	Barbosa, M. and Booth, S. 2009. East Timor's fisheries catch reconstruction (1950-2009): Fisheries under different regimes. pp. 39-51. *In*: D. Zeller and S. Harper (eds.) *Fisheries catch reconstructions: Islands, Part I.* Fisheries Centre Research Reports 17(5), University of British Columbia, Vancouver. (http://www.seaaroundus.org/doc/publications/chapters/2009/Barbosa-and-Booth-Timor-Leste.pdf).<br>To update the reconstruction the total reconstructed catch was carried forward, unaltered, to 2010. The FAO data constituted the reported portion of the catch. The unreported component was then taken to be the difference between the total reconstructed catch and the FAO data. Some amendments to the reconstructed dataset were also required, such as including the Cephalopods nei, Marine crabs nei, Natantian decapods nei, and Tropical spiny lobsters nei categories from the FAO data. In addition, all data was assigned as unreported, so first the correct amount of unreported artisanal catches were reassigned as reported, followed by subsistence if there was not enough artisanal. All reported catches were assigned as artisanal going forward, with the difference between the FAO reported data and the artisanal landings assigned as subsistence landings. All unreported catches were assigned as subsistence going forward. The taxonomic breakdown for both the reported and unreported components was based on the percentage breakdown in 2009.<br>[Indust. 1,1,1; Art. 2,2,2; Subs. 2,2,2; Recr. 1,1,1; Disc. 1,1,1]
229	Togo	2,4,4	(2) Belhabib, D., Kutoub, V. and Pauly, D. 2015. The marine fisheries of Togo, the 'heart of West Africa,' 1950 to 2010. pp. 37-50. *In:* D. Belhabib and D. Pauly (eds). *Fisheries catch reconstructions: West Africa, Part II.* Fisheries Centre Research Reports 23(3), University of British Columbia (http://www.seaaroundus.org/doc/publications/chapters/2015/Belhabib-Togo.pdf)<br>(4) Belhabib, D., Sumaila, U.R., Lam, V.W.Y., Zeller, D., Le Billon, P., Kane, E.A. and Pauly, D. 2015. Euro vs. Yuan: Comparing European and Chinese fishing access in West Africa.

Supplementary Table 5: Sources of reconstructions by country/EEZ component with associated publication links. Publication types are: 1: *Fisheries Centre Working Paper*, 2: *Fisheries Centre Research Reports*, 3: Other reports; 4: Primary literature. Uncertainty scores (as per Table S1) are given below the source of reconstruction for the three time periods 1950-1969, 1970-1989, 1990-2010 in square brackets by fishing sectors: Indust. = industrial, Art. = artisanal, Subs. = subsistence, Recr. = recreational, plus Disc. = discards.

#	Country	Publ. type	Source of reconstruction
			PLoS One 10(3): e0118351<br>(4) Belhabib, D., Sumaila, U.R. and Pauly, D. 2015. Feeding the poor: contribution of West African fisheries to employment and food security. *Ocean & Coastal Management* 111: 72-81.<br>[Indust. 3,3,3; Art. 3,3,3; Subs. 3,3,3; Recr. -,2,2; Disc. 1,1,2]
230	Tonga	2,4	(2) Sun, P., Harper, S., Booth, S. and Zeller, D. 2011. Reconstructing marine fisheries catches for the Kingdom of Tonga: 1950-2007. pp. 119-130. *In:* S. Harper and D. Zeller (eds.) *Fisheries catch reconstructions: Islands, Part II.* Fisheries Centre Research Reports 19(4), University of British Columbia, Vancouver. (http://www.seaaroundus.org/doc/publications/chapters/2011/Sun-et-al-2011-Tonga.pdf).<br>(4) Zeller, D., Harper, S., Zylich, K. and Pauly, D. 2015. Synthesis of under-reported small-scale fisheries catch in Pacific island waters. *Coral Reefs* 34(1): 25-39.<br>[Indust. 2,3,3; Art. 2,3,3; Subs. 2,2,2; Recr. 1,1,1; Disc. 2,2,2]
231	Trinidad and Tobago	1	Mohammed, E. and Lindop, M. 2014. Trinidad and Tobago: reconstructed fisheries catches, 1950-2010. Fisheries Centre Working Paper #2015-55, University of British Columbia, Vancouver, 42 p. (http://www.seaaroundus.org/doc/publications/wp/2015/Mohammed-and-Lindop-Trinidad-and-Tobago.pdf).<br>[Indust. 2,2,3; Art. 2,2,2; Subs. 1,1,1; Recr. -,-,-; Disc. 1,1,1]
232	Tunisia	1	Halouani, G., Lasram, F., Khalfallah, M., Zeller, D. and Pauly, D. 2015. Reconstruction of Marine Fisheries catches for Tunisia (1950-2010). Fisheries Centre Working Paper #2015-95, University of British Columbia, Vancouver, 11 p. (http://www.seaaroundus.org/doc/publications/wp/2015/Halouani-et-al-Tunisia.pdf).<br>[Indust. 2,2,3; Art. 2,2,3; Subs. 1,2,3; Recr. 1,1,1; Disc. 2,3,3]
233	Turkey (Black Sea)	4	Ulman, A., Bekişoğlu, Ş., Zengin, M., Knudsen, S., Ünal, V., Mathews, C., Harper, S., Zeller, D. and Pauly, D. 2013. From bonito to anchovy: a reconstruction of Turkey's marine fisheries catches (1950-2010). *Mediterranean Marine Science* 14(2): 309-342.<br>[Indust. 2,3,3; Art. 2,3,3; Subs. 1,2,2; Recr. 1,2,2; Disc. 3,3,4]
234	Turkey (Marmara Sea)	4	Ulman, A., Bekişoğlu, Ş., Zengin, M., Knudsen, S., Ünal, V., Mathews, C., Harper, S., Zeller, D. and Pauly, D. 2013. From bonito to anchovy: a reconstruction of Turkey's marine fisheries catches (1950-2010). *Mediterranean Marine Science* 14(2): 309-342.<br>[Indust. 2,3,3; Art. 2,3,3; Subs. 1,2,2; Recr. 1,2,2; Disc. 3,3,4]
235	Turkey (Mediterranean)	4	Ulman, A., Bekişoğlu, Ş., Zengin, M., Knudsen, S., Ünal, V., Mathews, C., Harper, S., Zeller, D. and Pauly, D. 2013. From bonito to anchovy: a reconstruction of Turkey's marine fisheries catches (1950-2010). *Mediterranean Marine Science* 14(2): 309-342.<br>[Indust. 2,3,3; Art. 2,3,3; Subs. 1,2,2; Recr. 1,2,2; Disc. 3,3,4]
236	Turks and Caicos Islands	1	Ulman, A., Burke, L., Hind, E., Ramdeen, R. and Zeller, D. 2015. Reconstruction of total marine fisheries catches for the Turks and Caicos Islands (1950 -2010). Fisheries Centre Working Paper #2015-63, University of British Columbia, Vancouver, 23 p.<br>[Indust. -,-,-; Art. 2,3,3; Subs. 2,3,4; Recr. 2,3,4; Disc. -,-,-]
237	Tuvalu	2,4	(2) Crawford, K., Harper, S. and Zeller, D. 2011. Reconstruction of marine fisheries catches for Tuvalu (1950-2009). pp. 131-143. *In:* S. Harper and D. Zeller (eds.) *Fisheries catch reconstructions: Islands, Part II.* Fisheries Centre Research Reports 19(4), University of British Columbia, Vancouver. (http://www.seaaroundus.org/doc/publications/chapters/2011/Crawford-et-al-Tuvalu.pdf).<br>(4) Zeller, D., Harper, S., Zylich, K. and Pauly, D. 2015. Synthesis of under-reported small-scale fisheries catch in Pacific island waters. *Coral Reefs* 34(1): 25-39.<br>[Indust. 1,2,3; Art. 1,2,3; Subs. 1,2,3; Recr. 1,1,1; Disc. 1,2,3]
238	Ukraine	1,4	(1) Ulman, A., Shlyakhov, V., Jatsenko, S. and Pauly, D. 2015. A reconstruction of the Ukraine's marine fisheries catches, 1950-2010. Fisheries Centre Working Paper #2015-86, University of British Columbia, Vancouver, 23 p. (http://www.seaaroundus.org/doc/publications/wp/2015/Ulman-et-al-Ukraine.pdf).<br>(4) Ulman, A., Shlyakhov, V., Jatsenko, S. and Pauly, D. 2015. A reconstruction of the Ukraine's marine fisheries catches, 1950-2010. *Journal of the Black Sea and Mediterranean Environment* 21(2): 103-124.<br>[Indust. 2.5,3,2; Art. 3,4,2; Subs. 1,1,2; Recr. 1,1,2; Disc. -,-,-]
239	United Arab Emirates	2,4	(2) Al-Abdulrazzak, D. 2013. Estimating total fish extractions in the United Arab Emirates: 1950-2010. p. 53-59. *In*: D. Al-Abdulrazzak and D. Pauly (eds.) *From dhows to trawlers: a recent history of fisheries in the Gulf countries, 1950 to 2010.* Fisheries Centre Research Reports 21(2), University of British Columbia, Vancouver. (http://www.seaaroundus.org/doc/publications/chapters/2013/AlAbdulrazzak-UAE.pdf).<br>(4) Al-Abdulrazzak, D., Zeller, D., Belhabib, D., Tesfamichael, D. and Pauly, D. 2015. Total marine fisheries catches in the Persian/Arabian Gulf from 1950-2010. *Regional Studies in Marine Science* 2: 28-34.<br>[Indust. -,-,-; Art. 3,3,3; Subs. 1,1,1; Recr. 1,1,1; Disc. 2,2,3]
240	United Arab	1	Khalfallah, M., Zeller, D. and Pauly, D. 2015. Reconstruction of marine fisheries catches for

Supplementary Table 5: Sources of reconstructions by country/EEZ component with associated publication links. Publication types are: 1: *Fisheries Centre Working Paper*, 2: *Fisheries Centre Research Reports*, 3: Other reports; 4: Primary literature. Uncertainty scores (as per Table S1) are given below the source of reconstruction for the three time periods 1950-1969, 1970-1989, 1990-2010 in square brackets by fishing sectors: Indust. = industrial, Art. = artisanal, Subs. = subsistence, Recr. = recreational, plus Disc. = discards.

#	Country	Publ. type	Source of reconstruction
	Emirates (Gulf of Oman)		Fujairah (UAE) (1950-2010). Fisheries Centre Working Paper #2015-57, University of British Columbia, Vancouver, 13 p. (http://www.seaaroundus.org/doc/publications/wp/2015/Khalfallah-et-al-Fujairah.pdf).<br>[Indust. 2,3,3; Art. 2,3,3; Subs. 2,2,2; Recr. 1,1,1; Disc. 1,2,2]
241	United Kingdom	1	Gibson, D., Cardwell, E., Zylich, K. and Zeller, D. 2015. Preliminary reconstruction of total marine fisheries catches for the United Kingdom and the Channel Islands in EEZ equivalent waters (1950-2010). Fisheries Centre Working Paper #2015-76, University of British Columbia, Vancouver, 20 p. (http://www.seaaroundus.org/doc/publications/wp/2015/Gibson-et-al-UK-and-Channel-Is.pdf).<br>[Indust. 3,3,3; Art. 2,2,3; Subs. 1,1,1; Recr. 2,2,2; Disc. 2,2,2]
242	United Kingdom (Anguilla)	2	Ramdeen, R., Zylich, K. and Zeller, D. 2014. Reconstruction of total marine fisheries catches for Anguilla (1950-2010). pp. 1-8. *In:* K. Zylich, D. Zeller, M. Ang, and D. Pauly (eds.) *Fisheries catch reconstructions: Islands, Part IV.* Fisheries Centre Research Reports 22(2), University of British Columbia, Vancouver. (http://www.seaaroundus.org/doc/publications/chapters/2014/Ramdeen-et-al-Anguilla.pdf).<br>[Indust. 2,3,3; Art. 2,3,3; Subs. 2,2,2; Recr. 1,1,1; Disc. 2,2,2]
243	United Kingdom (Ascension)	2	Booth, S. and Azar, H. 2009. The fisheries of St Helena and its dependencies. p. 27-34. *In*: D. Zeller and S. Harper (eds.) *Fisheries catch reconstructions: Islands, Part I.* Fisheries Centre Research Reports 17(5), University of British Columbia, Vancouver. (http://www.seaaroundus.org/doc/publications/chapters/2009/Booth-and-Azar-St-Helena.pdf).<br>Since completing the initial reconstruction, FAO data became available to 2010. To update the Ascension Island reconstruction, the 2010 total reconstructed catch was estimated using this formula: (population*per capita kg/1000)-imports, and the data points between 2006 and 2010 were generated using linear interpolation. A population estimates for 2010 was combined with the per capita catch rate and imports were used to update trends to 2010. The taxonomic breakdown used for Saint Helena was adopted for this island.<br>[Indust. 1,2,2; Art. 1,2,2; Subs. 1,2,2; Recr. 1,1,1; Disc. 2,2,2]
244	United Kingdom (Bermuda)	1	Divovich, E., Teh, L.C.L., Zylich, K. and Zeller, D. 2015. Updated reconstruction of Bermuda's marine fisheries catches, 1950-2010. Fisheries Centre Working Paper #2015-96, University of British Columbia, Vancouver, 18 p. (http://www.seaaroundus.org/doc/publications/wp/2015/Divovich-et-al-Bermuda.pdf).<br>[Indust. -,-,-; Art. 2,3,3; Subs. 2,2,2; Recr. 1,2,2; Disc. -,-,-]
245	United Kingdom (Cayman Islands)	2	Harper, S., Bothwell, J., Bale, S., Booth, S. and Zeller, D. 2009. Cayman Island fisheries catches: 1950-2007. pp. 3-11. *In:* D. Zeller and S. Harper (eds.) *Fisheries catch reconstructions: Islands, Part I.* Fisheries Centre Research Reports 17(5), University of British Columbia, Vancouver. (http://www.seaaroundus.org/doc/publications/chapters/2009/Harper-et-al-Cayman.pdf).<br>To update this reconstruction the 2007 total reconstructed catch was carried forward, unaltered, to 2010. The FAO data constituted the reported portion of the catch. The unreported catch component for 2008-2010 was taken to be the difference between the 2007 total reconstructed catch amount and the FAO totals. The reported component was assigned to the artisanal sector and split 50/50 between the EEZs of Honduras and Colombia (which follows the pattern of the original reconstruction). The unreported component was assigned to sectors as follows: 1 t to subsistence, ~ 25 t to recreational, 1 t to artisanal in Cayman Island's EEZ, ~ 0.78 t to artisanal in Honduras's EEZ. This also follows the pattern of the original reconstruction. The taxonomic breakdowns remained the same for all sectors for both the reported and unreported components. Please note that the values and comparisons for the years 1950-2007 were based on the 2007 FAO dataset, and changes were not made to account for small differences within the 2010 dataset regarding previous years.<br>[Indust. -,-,-; Art. 2,2,2; Subs. 2,2,2; Recr. -,2,2; Disc. -,-,-]
246	United Kingdom (Chagos Archipelago)	2	Zeller, D. and Pauly, D. 2014. Reconstruction of domestic fisheries catches in the Chagos Archipelago: 1950-2010. pp. 17-24 *In:* K. Zylich, D. Zeller, M. Ang and D. Pauly (eds.) *Fisheries catch reconstructions: Islands, Part IV.* Fisheries Centre Research Reports 22(2). University of British Columbia, Vancouver. (http://www.seaaroundus.org/doc/publications/chapters/2014/Zeller-and-Pauly-Chagos.pdf).<br>[Indust. -,-,-; Art. -,-,-; Subs. 1,1,2; Recr. -,-,1; Disc. -,-,-]
247	United Kingdom (Channel Islands)	1	Gibson, D., Cardwell, E., Zylich, K. and Zeller, D. 2015. Preliminary reconstruction of total marine fisheries catches for the United Kingdom and the Channel Islands in EEZ equivalent waters (1950-2010). Fisheries Centre Working Paper #2015-76, University of British Columbia, Vancouver, 20 p.

Supplementary Table 5: Sources of reconstructions by country/EEZ component with associated publication links. Publication types are: 1: *Fisheries Centre Working Paper*, 2: *Fisheries Centre Research Reports*, 3: Other reports; 4: Primary literature. Uncertainty scores (as per Table S1) are given below the source of reconstruction for the three time periods 1950-1969, 1970-1989, 1990-2010 in square brackets by fishing sectors: Indust. = industrial, Art. = artisanal, Subs. = subsistence, Recr. = recreational, plus Disc. = discards.

#	Country	Publ. type	Source of reconstruction
			(http://www.seaaroundus.org/doc/publications/wp/2015/Gibson-et-al-UK-and-Channel-Is.pdf).<br>[Indust. 3,3,2; Art. 2,2,2; Subs. 1,1,1; Recr. 1,1,1; Disc. 2,2,2]
248	United Kingdom (Falklands Islands)	2	Palomares, M.L.D. and Pauly, D. 2015. Reconstruction of the marine fisheries catches of the Falkland Islands and the British Antarctic Territories 1950-2010. *In:* M.L.D. Palomares and D. Pauly (eds.) *Marine Fisheries Catches of Sub- Antarctic Islands, 1950 to 2010.* pp. 1-19. Fisheries Centre Research Reports 23(1), University of British Columbia, Vancouver. (http://www.seaaroundus.org/doc/publications/chapters/2015/Palomares-and-Pauly-2015-Falkland-Islands.pdf).<br>[Indust. 1,3,4; Art. 1,1,1; Subs. -,-,-; Recr. -,-,-; Disc. 1,3,4]
249	United Kingdom (Pitcairn Islands)	2,4	(2) Chaitanya, D., Harper, S. and Zeller, D. 2012. Reconstruction of total marine fisheries catches for the Pitcairn Islands (1950-2009), pp. 87-94. *In:* S. Harper, K. Zylich, L. Boonzaier, F. Le Manach, D. Pauly and D. Zeller (eds.) *Fisheries catch reconstructions: Islands, Part III.* Fisheries Centre Research Reports 20(5), University of British Columbia, Vancouver. (http://www.seaaroundus.org/doc/publications/chapters/2012/Chaitanya-et-al-Pitcairn-Islands.pdf).<br>(4) Zeller, D., Harper, S., Zylich, K. and Pauly, D. 2015. Synthesis of under-reported small-scale fisheries catch in Pacific island waters. *Coral Reefs* 34(1): 25-39.<br>Incl. on p. 91 a short addendum which updates the dataset to 2010.<br>[Indust. 2,3,2; Art. 2,3,3; Subs. 2,3,3; Recr. 1,1,1; Disc. 1,1,1]
250	United Kingdom (St Helena)	2	Booth, S. and Azar, H. 2009. The fisheries of St Helena and its dependencies. pp. 27-34. *In*: D. Zeller and S. Harper (eds) *Fisheries catch reconstructions: Islands, Part I.* Fisheries Centre Research Reports 17(5), University of British Columbia, Vancouver. (http://www.seaaroundus.org/doc/publications/chapters/2009/Booth-and-Azar-St-Helena.pdf).<br>Since completing the initial reconstruction, FAO data became available to 2010. To update the reconstruction, the FAO data except for the Tristan da Cunha rock lobster and Octopuses, etc. nei categories were used as the reported component. However, in the initial reconstruction the reported data for 1950-1977 were disregarded. Therefore, we reassigned the correct amount of artisanal unreported catches as reported catches for that time period. The ratio between the FAO reported component and total reconstructed component for 2006 was calculated and applied to the FAO data for 2007 to 2010 to estimate the total reconstructed catch for those years. The unreported component for 2007 to 2010 was then taken to be the difference between the two numbers. All of the reported data for 2007 to 2010 was assigned as industrial, and the sectoral breakdown for the unreported component was based on the percentage breakdown in 2006. The taxonomic breakdown for the unreported component was based on the percentage breakdown in 2006 (by sector).<br>[Indust. 1,2,2; Art. 1,2,2; Subs. 1,2,2; Recr. 1,1,1; Disc. 2,2,2]
251	United Kingdom (South Georgia/South Sandwich Islands)	2	Palomares, M.L.D. and Pauly, D. 2015. Reconstruction of the marine fisheries catches of the Falkland Islands and the British Antarctic Territories 1950-2010. *In:* M.L.D. Palomares and D. Pauly (eds.) *Marine Fisheries Catches of Sub- Antarctic Islands, 1950 to 2010.* pp. 1-19. Fisheries Centre Research Reports 23(1), University of British Columbia, Vancouver. (http://www.seaaroundus.org/doc/publications/chapters/2015/Palomares-and-Pauly-2015-Falkland-Islands.pdf).<br>[Indust. 2,3,3; Art. 2,2,2; Subs. 1,2,2; Recr. 1,1,1; Disc. 2,2,2]
252	United Kingdom (South Orkney Islands)	2	Palomares, M.L.D. and Pauly, D. 2015. Reconstruction of the marine fisheries catches of the Falkland Islands and the British Antarctic Territories 1950-2010. *In:* M.L.D. Palomares and D. Pauly (eds.) *Marine Fisheries Catches of Sub- Antarctic Islands, 1950 to 2010.* pp. 1-19. Fisheries Centre Research Reports 23(1), University of British Columbia, Vancouver. (http://www.seaaroundus.org/doc/publications/chapters/2015/Palomares-and-Pauly-2015-Falkland-Islands.pdf).<br>[Indust. 2,3,3; Art. -,-,-; Subs. -,-,-; Recr. -,-,-; Disc. 2,3,3]
253	United Kingdom (Tristan da Cunha)	2	Booth, S. and Azar, H. 2009. The fisheries of St Helena and its dependencies. pp. 27-34 *In*: D. Zeller and S. Harper (eds.) *Fisheries catch reconstructions: Islands, Part I.* Fisheries Centre Research Reports 17(5), University of British Columbia, Vancouver. (http://www.seaaroundus.org/doc/publications/chapters/2009/Booth-and-Azar-St-Helena.pdf).<br>Since completing the initial reconstruction, FAO data became available to 2010. To update the reconstruction, the FAO data for Tristan da Cunha rock lobster and Octopuses, etc. nei were used as the reported component. The ratio between the FAO reported component and total reconstructed catch for 2005 was applied to FAO categories for 2006 to 2010 to estimate the total reconstructed catch for those years. The unreported component was then taken to be the difference between the two numbers. All of the reported data was assigned as industrial, and the sectoral breakdown for the unreported component was based on the percentage breakdown in 2005. The taxonomic breakdown for the unreported component was based on the percentage breakdown in 2005 (by sector).

Supplementary Table 5: Sources of reconstructions by country/EEZ component with associated publication links. Publication types are: 1: *Fisheries Centre Working Paper*, 2: *Fisheries Centre Research Reports*, 3: Other reports; 4: Primary literature. Uncertainty scores (as per Table S1) are given below the source of reconstruction for the three time periods 1950-1969, 1970-1989, 1990-2010 in square brackets by fishing sectors: Indust. = industrial, Art. = artisanal, Subs. = subsistence, Recr. = recreational, plus Disc. = discards.

#	Country	Publ. type	Source of reconstruction
			[Indust. 1,2,2; Art. 1,2,2; Subs. 1,2,2; Recr. 1,1,1; Disc. 2,2,2]
254	United Kingdom (Virgin Islands)	2	Ramdeen, R., Harper, S., Zylich, K. and Zeller, D. 2014. Reconstruction of total marine fisheries catches for the British Virgin Islands (1950-2010). pp. 9-16. *In*: K. Zylich, D. Zeller, M. Ang and D. Pauly (eds.) *Fisheries catch reconstructions: Islands, Part IV.* Fisheries Centre Research Reports 22(2), University of British Columbia, Vancouver. (http://www.seaaroundus.org/doc/publications/chapters/2014/Ramdeen-et-al-BVI.pdf).<br>[Indust. 2,2,1; Art. 2,2,1; Subs. 1,1,1; Recr. 1,1,1; Disc. 1,1,1]
255	Uruguay	1	Lorenzo, M.I., Defeo, O., Roshan Moniri, N. and Zylich, K. 2014. Fisheries catch statistics for Uruguay. Fisheries Centre Working Paper #2015-25, University of British Columbia, Vancouver, 6 p. (http://www.seaaroundus.org/doc/publications/wp/2015/Lorenzo-et-al-Uruguay.pdf).<br>[Indust. 2,3,4; Art. 2,2,3; Subs. 2,3,3; Recr. 1,2,2; Disc. 1,1,2]
256	USA (American Samoa)	4,4	(4) Zeller, D., Booth, S., Craig, P. and Pauly, D. 2006. Reconstruction of coral reef fisheries catches in American Samoa, 1950-2002. *Coral Reefs* 25: 144-152.<br>(4) Zeller, D., Harper, S., Zylich, K. and Pauly, D. 2015. Synthesis of under-reported small-scale fisheries catch in Pacific island waters. *Coral Reefs* 34(1): 25-39.<br>[Indust. -,-,-; Art. 2,3,3; Subs. 1,1,1; Recr. -,-,-; Disc. -,-,-]
257	USA (Alaska, Arctic)	1,2,4	(1) Teh, L.C.L., Zylich, K. and Zeller, D. 2015. FAO area 18 (Arctic Sea): Catch data reconstruction extension of Zeller *et al.* (2011) to 2010. Fisheries Centre Working Paper #2015-14, University of British Columbia, Vancouver, 5 p. (http://www.seaaroundus.org/doc/publications/wp/2015/Teh-et-al-Arctic-Sea.pdf).<br>(2) Booth, S. and Zeller, D. 2008. *Marine fisheries catches in Arctic Alaska.* Fisheries Centre Research Reports 16(9), University of British Columbia, Vancouver. (http://www.fisheries.ubc.ca/webfm_send/137).<br>(4) Zeller, D., Booth, S., Pakhomov, E., Swartz, W. and Pauly, D. 2011. Arctic fisheries catches in Russia, USA and Canada: Baselines for neglected ecosystems. *Polar Biology* 34(7): 955-973.<br>[Indust. 3,3,3; Art. 4,4,4; Subs. 4,4,4; Recr. -,-,-; Disc. 2,3,3]
258	USA (Alaska, Subarctic)	1	Doherty, B., Gibson, D., Zhai, Y., McCrea-Strub, A., Zylich, K., Zeller, D. and Pauly, D. 2015. Reconstruction of marine fisheries catches for Subarctic Alaska, 1950-2010. Fisheries Centre Working Paper #2015-82, University of British Columbia, Vancouver, 34 p. (http://www.seaaroundus.org/doc/publications/wp/2015/Doherty-et-al-Alaska.pdf).<br>[Indust. 3,3,4; Art. 3,3,4; Subs. 1,1,2; Recr. 1,2,3; Disc. 1,2,4]
259	USA (East Coast)	1	McCrea-Strub, A. 2015. Reconstruction of total catch by U.S. fisheries in the Atlantic and Gulf of Mexico: 1950-2010. Fisheries Centre Working Paper #2015-79, University of British Columbia, Vancouver, 46 p. (http://www.seaaroundus.org/doc/publications/wp/2015/McCrea-Strub-US-Atlantic-and-Gulf.pdf).<br>[Indust. 4,4,4; Art. 4,4,4; Subs. -,-,-; Recr. 2,3,4; Disc. 2,2,3]
260	USA (Guam)	4,4	(4) Zeller, D., Booth, S., Davis, G. and Pauly, D. 2007. Re-estimation of small-scale for U.S. flag-associated islands in the western Pacific: the last 50 years. *U.S. Fisheries Bulletin* 105: 266-277<br>(4) Zeller, D., Harper, S., Zylich, K. and Pauly, D. 2015. Synthesis of under-reported small-scale fisheries catch in Pacific island waters. *Coral Reefs* 34(1): 25-39.<br>[Indust. 2,2,2; Art. 2,2,2; Subs. 1,1,1; Recr. 1,1,1; Disc. 2,2,2]
261	USA (Gulf of Mexico)	1	McCrea-Strub, A. 2015. Reconstruction of total catch by U.S. fisheries in the Atlantic and Gulf of Mexico: 1950-2010. Fisheries Centre Working Paper #2015-79, University of British Columbia, Vancouver, 46 p. (http://www.seaaroundus.org/doc/publications/wp/2015/McCrea-Strub-US-Atlantic-and-Gulf.pdf).<br>[Indust. 4,4,4; Art. 4,4,4; Subs. -,-,-; Recr. 2,3,4; Disc. 3,4,4]
262	USA (Main Hawaiian Islands)	1,4	(1) Gibson, D., McCrea-Strub, A. and Zeller, D. 2015. Updated reconstruction of Hawaiian fisheries 1950-2010. Fisheries Centre Working Paper #2015-83, University of British Columbia, Vancouver, 8 p. (http://www.seaaroundus.org/doc/publications/wp/2015/Gibson-et-al-Hawaii.pdf).<br>(4) Zeller, D., Harper, S., Zylich, K. and Pauly, D. 2015. Synthesis of under-reported small-scale fisheries catch in Pacific island waters. *Coral Reefs* 34(1): 25-39.<br>[Indust. 3,3,4; Art. 3,3,3; Subs. 2,2,2; Recr. 2,2,2; Disc. 1,2,2]
263	USA (Northern Mariana Islands)	4	Zeller, D., Booth, S., Davis, G. and Pauly, D. 2007. Re-estimation of small-scale for U.S. flag-associated islands in the western Pacific: the last 50 years. *U.S. Fisheries Bulletin* 105: 266-277.<br>[Indust. 2,3,4; Art. 2,3,4; Subs. 2,2,2; Recr. 1,1,1; Disc. 2,3,3]
264	USA (Northwestern	1,4	(1) Gibson, D., McCrea-Strub, A. and Zeller, D. 2015. Updated reconstruction of Hawaiian fisheries 1950-2010. Fisheries Centre Working Paper #2015-83, University of British

Supplementary Table 5: Sources of reconstructions by country/EEZ component with associated publication links. Publication types are: 1: *Fisheries Centre Working Paper*, 2: *Fisheries Centre Research Reports*, 3: Other reports; 4: Primary literature. Uncertainty scores (as per Table S1) are given below the source of reconstruction for the three time periods 1950-1969, 1970-1989, 1990-2010 in square brackets by fishing sectors: Indust. = industrial, Art. = artisanal, Subs. = subsistence, Recr. = recreational, plus Disc. = discards.

#	Country	Publ. type	Source of reconstruction
	Hawaiian Islands)		Columbia, Vancouver, 8 p. (http://www.seaaroundus.org/doc/publications/wp/2015/Gibson-et-al-Hawaii.pdf).<br>(4) Zeller, D., Harper, S., Zylich, K. and Pauly, D. 20154. Synthesis of under-reported small-scale fisheries catch in Pacific -island waters. *Coral Reefs* 34(1): 25-39.<br>[Indust. 1,2,3; Art. -,-,-; Subs. -,-,-; Recr. -,-,-; Disc. 1,1,1]
265	USA (Pacific Small Island Territories)	4	Zeller, D., Harper, S., Zylich, K. and Pauly, D. 2015. Synthesis of under-reported small-scale fisheries catch in Pacific -island waters. *Coral Reefs* 34(1): 25-39.<br>[Indust. -,-,-; Art. -,-,-; Subs. -,-,-; Recr. 1,1,1; Disc. -,-,-]
266	USA (Puerto Rico)	1	Appeldoorn, R. and Sanders, I. 2015. A 61 year reconstruction of fisheries catch in Puerto Rico. Fisheries Centre Working Paper #2015-44, University of British Columbia, Vancouver, 15 p. (http://www.seaaroundus.org/doc/publications/wp/2015/Appeldoorn-and-Sanders-Puerto-Rico.pdf).<br>[Indust. 2,3,3; Art. 3,3,3; Subs. 2,2,2; Recr. 2,2,2; Disc. 2,2,2]
267	USA (Virgin Islands)	1	Ramdeen, R., Zylich, K. and Zeller, D. 2015. Reconstruction of total marine catches for the US Virgin Islands (1950-2010). Fisheries Centre Working Paper #2015-64, University of British Columbia, Vancouver, 9 p. (http://www.seaaroundus.org/doc/publications/wp/2015/Ramdeen-et-al-US-Virgin-Is.pdf).<br>[Indust. 2,3,3; Art. 2,3,3; Subs. 2,2,2; Recr. 1,1,1; Disc. 2,3,3]
268	USA (West Coast)	1	Doherty, B., Harguth, H., McCrea-Strub, A., Jenkins, L.D. and Figueira, W. 2015. Reconstructing catches along Highway 101: Historic catch estimates for marine fisheries in California, Oregon and Washington from 1950-2010. Fisheries Centre Working Paper #2015-81, University of British Columbia, Vancouver, 66 p. (http://www.seaaroundus.org/doc/publications/wp/2015/Doherty-et-al-US-West.pdf).<br>[Indust. 4,4,4; Art. 4,4,4; Subs. -,-,-; Recr. 2,3,3; Disc. 2,1,2]
269	Vanuatu	2,4	(2) Zylich, K., Shon, S., Harper, S. and Zeller, D. 2014. Reconstruction of total marine fisheries catches for the Republic of Vanuatu, 1950-2010. pp. 147-156. *In*: K. Zylich, D. Zeller, M. Ang and D. Pauly (eds.) *Fisheries catch reconstructions: Islands, Part IV.* Fisheries Centre Research Reports 22(2), University of British Columbia, Vancouver. (http://www.seaaroundus.org/doc/publications/chapters/2014/Zylich-et-al-Vanuatu.pdf).<br>(4) Zeller, D., Harper, S., Zylich, K. and Pauly, D. 2015. Synthesis of under-reported small-scale fisheries catch in Pacific island waters. *Coral Reefs* 34(1): 25-39.<br>[Indust. 1,2,2; Art. 2,3,3; Subs. 2,2,2; Recr. 1,1,1; Disc. 2,3,3]
270	Venezuela	1	Mendoza, J. 2015. Rise and fall of Venezuelan industrial and artisanal marine fisheries: 1950-2010. Fisheries Centre Working Paper #2015-27, University of British Columbia, Vancouver, 15 p. (http://www.seaaroundus.org/doc/publications/wp/2015/Mendoza-Venezuela.pdf).<br>[Indust. 3,3,3; Art. 1,2,2; Subs. 1,1,1; Recr. 2,3,3; Disc. 1,2,3]
271	Vietnam	1	Teh, L.C.L., Zeller, D., Zylich, K., Nguyen, G. and Harper, S. 2014. Reconstructing Vietnam's Marine Fisheries Catch, 1950-2010. Fisheries Centre Working Paper #2014-17, University of British Columbia, Vancouver, 11 p. (http://www.seaaroundus.org/doc/publications/wp/2014/Teh-et-al-Vietnam.pdf).<br>[Indust. -,-,2; Art. 2,2,1; Subs. 2,2,1; Recr. -,-,-; Disc. -,2,2]
272	Yemen (Gulf of Aden)	2	Tesfamichael, D., Rossing, P. and Awadh, H. 2012. Reconstruction of Yemen's catches in the Gulf of Aden, 1950–2010. pp. 135-152. *In:* D. Tesfamichael and D. Pauly (eds.) *Catch reconstruction for the Red Sea large marine ecosystem by countries (1950-2010)*. Fisheries Centre Research Reports 20(1), University of British Columbia, Vancouver. (http://www.seaaroundus.org/doc/publications/chapters/2012/Tesfamichael-Yemen.pdf).<br>[Indust. 2,3,3; Art. 3,3,3; Subs. 1,1,1; Recr. 1,1,1; Disc. 2,2,2]
273	Yemen (Red Sea)	2	Tesfamichael, D., Rossing, P. and Awadh, H. 2012. The marine fisheries of Yemen with emphasis on the Red Sea and cooperatives. pp. 105-134. *In:* D. Tesfamichael and D. Pauly (eds.) *Catch reconstruction for the Red Sea large marine ecosystem by countries (1950-2010)*. Fisheries Centre Research Reports 20(1), University of British Columbia, Vancouver. (http://www.seaaroundus.org/doc/publications/chapters/2012/Tesfamichael-Yemen-Gulf.pdf).<br>[Indust. 2,3,3; Art. 3,3,3; Subs. 1,1,1; Recr. 1,1,1; Disc. 2,2,2]

Supplementary Table 6. Sources used in the creation of tuna and large pelagic dataset (Layer 3), by ocean.

**Ocean basin**		**Atlantic**	**Indian**	**Pacific**		**Southern**[a]
				**Eastern**	**Western**	
**RFMO**		**ICCAT**	**IOTC**	**IATTC**	**WCPFC**	**CCSBT**
Data	Nominal	website	website	website	website	Provided by staff
	Spatialized	website	website	www.fao.org/figis/geoserver/tunaatlas	website	website
Spatial resolution		1°x1°<br>5°x5°<br>5°x10°<br>10°x10°<br>10°x20°<br>20°x20°	1°x1°<br>5°x5°<br>10°x10°<br>10°x20°<br>20°x20°	5°x5° ([b])	5°x5°	5°x5°
Number of	Countries	114	57	28	41	11
	Gears	48	35	11	9	8
	Species	142	45	19	9	1

[a] This RFMO covers all three oceans, but only deals with southern bluefin tuna (*Thunnus maccoyii*). Note that the other RFMOs also sometimes report this species (here not considered as double-counting).

[b] A number of these cells straddle the Pacific and Atlantic Oceans. Their catch was split into these two ocean basins in proportion to the surface of the cells included in each ocean.

Supplementary Acknowledgements

A large number of colleagues around the world have contributed to country catch reconstructions and other database work, including: Vicky Lam[1], Mohammed Abudaya[2], David G. Ainley[3], Cameron Ainsworth[4], Dalal Al-Abdulrazzak[1], Juan José Alava [5], Melanie Ang[1], Richard Appledoorn[6], Andrea Au[1], Sarah Bale[1], Milton Barbosa[1], Sebastian Baust[1], Dyhia Belhabib[1], Brajgeet Bhathal[1], Léa Boistol[1], Aleš Bolje[7], Lisa Boonzaier[1], Shawn Booth[1], Lucas Brotz[2], Vania Budimartono[1], Elise Bultel[1], Daniela Bănaru[8], Emma Cardwell[9], Marta Carreras[10], José J. Castro[11], Devraj Chaitanya[1], William W. L. Cheung[12], Elviro A. Cinco[13], Andrés M. Cisneros-Montemayor[14], Marta Coll[15], Mathieu Colléter[1], Loida Corpus [1], Kendyl Crawford[16], Esther Divovich[1], Beau Doherty[1], Rodrigo Donadi[17], Bridget Doyle[1], Dori Edelist[18], Lawrence Etim[19], Kátia de Meirelles Felizola Freire[20], Rainer Froese[21], Lou Frotté[22], Manuela Funes[1], Jeanel Georges[23], Darah Gibson[1], Krista Greer[1], Andrea Haas[1], Ghassen Halouani[24], Sarah Harper[1], Mark Hemmings[25], Claire Hornby[1], Davis Iritani[1], Manuel Ixquiac-Cabrera[26], Jennifer L. Jacquet[1], Boris Jovanović[1], Çetin Keskin[27], Myriam Khalfallah[1], Kristin M. Kleisner[1], Daniel Kuo[1], Frédéric Le Manach[1], Francisco Leitão[28], Ann-Katrien Lescrauwaet[29], Alasdair Lindop[1], Stephanie Lingard[1], María Inés Lorenzo[30], Jessica MacDonald[1], Hatem H. Mahmoud[31], Sanja

[1] *Sea Around Us*, Global Fisheries Cluster, University of British Columbia, Vancouver, BC, V6T 1ZA, Canada
[2] University of Palestine, Faculty of Applied Engineering, Al Zahra, Gaza, Palestine
[3] H.T. Harvey & Associates, Los Gatos, CA 95032, USA
[4] College of Marine Science, University of South Florida, St. Petersburg, USA
[5] School of Resource and Environmental Management, Simon Fraser University, Burnaby, BC, V5A 1S6, Canada
[6] Department of Marine Sciences, University of Puerto Rico, Mayagüez, Puerto Rico 00681-9000, USA
[7] Fisheries Research Institute of Slovenia, Spodnje Gameljne 61a, 1211 Ljubljana-Šmartno, Slovenia
[8] Aix-Marseille Université, Institut Méditerranéen d'Océanologie, Campus de Luminy, 13 288 Marseille, France
[9] School of Geography and Environment, Oxford University Centre for the Environment, Oxford, OX1 3QY, England
[10] OCEANA, Plaza España Leganitos, 47. 28013. Madrid, Spain
[11] Department of Biology, University of Las Palmas de Gran Canaria, Canary Islands, Spain
[12] Changing Ocean Research Unit, Global Fisheries Cluster, University of British Columbia, Vancouver, Canada
[13] Department of Fisheries, Ministry of Industry and Primary Resources, Berakas BB3910, Brunei Darussalam
[14] Fisheries Economics Research Unit, Global Fisheries Cluster, University of British Columbia. Vancouver, Canada
[15] Institut de Ciències del Mar (ICM-CSIC), Passeig Marítim de la Barceloneta, n° 37-49, Barcelona 08003, Spain
[16] Department of Marine and Environmental Science, Hampton University, Hampton, Virginia 23668, USA
[17] WWF Panama, Ciudad del Saber – Building 235, Clayton, Republic of Panama
[18] Department of Maritime Civilizations, Faculty of Natural Sciences, University of Haifa, Haifa 31905, Israel
[19] Department of Fisheries and Aquatic Environmental Management, University of Uyo, Akwa Ibom State, Nigeria
[20] Universidade Federal de Sergipe, Departamento de Engenharia de Pesca e Aquicultura, São Cristóvão, Brazil
[21] GEOMAR, Helmholtz-Zentrum für Ozeanforschung, Düsternbrooker Weg 20, 24148 Kiel, Germany
[22] Muséum National d'Histoire Naturelle, Station Marine de Concarneau, France
[23] University of the West Indies, Cave Hill, St. Michael, Barbados
[24] National Agronomic Institute of Tunisia, 43 Av. Charles Nicolle 1082 Tunis, Tunisia
[25] School of Marine Science and Engineering, Plymouth University, Drake Circus, Plymouth, PL4 8AA
[26] Universidad de San Carlos de Guatemala, Ciudad Universitaria, Guatemala
[27] Faculty of Fisheries, University of Istanbul, Laleli, Istanbul, Turkey
[28] Centro de Ciências do Mar, Universidade do Algarve, Campus de Gambelas, 8005-139 Faro, Portugal
[29] Flanders Marine Institute VLIZ, Wandelaarkaai 7, 8400 Oostende Belgium
[30] Dirección Nacional de Recursos Acuáticos. Constituyente 1497, 11200 Montevideo, Uruguay

Matić-Skoko[32], Ashley McCrea-Strub[1], Jaime Mendo[33], Jeremy J. Mendoza[34], Dana D. Miller[14], Elizabeth Mohammed[35], Telmo Morato[36], Dimitrios K. Moutopoulos[37], Manal R. Nader[38], Kjell Nedreaas[39], Francis K. E. Nunoo[40], Devon O'Meara[1], Allan Padilla[1], Maria-Lourdes Palomares[1], Lo Persson[1], Christopher K. Pham[41], Chiara Piroddi[42], Patrice Pruvost[43], Robin Ramdeen[1], Nardin Roshan Moniri[1], Peter Rossing[1], Isaac Trindade Santos[44], Laurenne Schiller[1], Katherine Seto[45], Soohyun Shon[1], Glenn Simmons[46], Nicola S. Smith[47], Anna Sobolevskaya[48], Konstantinos I. Stergiou[49], Patricia Sun[1], Wilf Swartz[50], Louise S.L. Teh[14], Lydia C.L. Teh[1], Dawit Tesfamichael[1], Pablo Trujillo[1], Hadayet Ullah[51], Aylin Ulman[1], Sadiq Vali[1], Hreiðar Þór Valtýsson[52], Liesbeth van der Meer[1], Liane Veitch[1], Sebastian Villasante[53], Kyrstn Zylich[1]

[31] College of Fisheries Technology & Aquaculture, Arab Academy for Science, Technology & Maritime Transport, Alexandria, Egypt
[32] Institute of Oceanography and Fisheries, Meštrovićevo šetalište 63, P.O.Box. 500, 21000 Split, Croatia
[33] Facultad de Pesquería, Universidad Nacional Agraria La Molina, Av. La Molina, Lima, Perú
[34] Instituto Oceanográfico de Venezuela, Universidad de Oriente, Cumaná, Venezuela
[35] Caribbean Regional Fisheries Mechanism Secretariat, Eastern Caribbean Office, St Vincent and the Grenadines
[36] Department of Oceanography and Fisheries-University of the Azores, Horta, Azores, Portugal
[37] Technological Educational Institute of Western Greece, Department of Aquaculture and Fisheries, Greece
[38] Institute of the Environment, University of Balamand, Lebanon
[39] Institute of Marine Research, P.O. Box 1870, 5817 Bergen
[40] Department of Marine and Fisheries Sciences, University of Ghana, P.O. Box LG 99, Legon, Accra, Ghana
[41] Departamento de Oceanografia e Pescas, Universidade dos Açores, Horta, Portugal
[42] Joint Research Centre, European Commission, Ispra, Italy
[43] Muséum National d'Histoire Naturelle, Paris; France
[44] Instituto Nacional de Desenvolvimento das Pescas (INDP), Mindelo, Cabo Verde
[45] Department of Environmental Science, Policy, and Management, University of California at Berkeley, Berkeley, California, USA
[46] New Zealand Asia Institute, The University of Auckland, Private Bag 92019, Auckland, New Zealand
[47] Department of Marine Resources, Ministry of Agriculture, Marine Resources and Local Government, Nassau, Bahamas
[48] Institute of East Asian Studies, Duisburg–Essen University, Germany
[49] Institute of Marine Biological Resources and Inland Waters, Sounio Ave., 19013 Anavissos Attica, Greece
[50] Nereus Program, University of British Columbia, Vancouver, Canada
[51] WorldFish – Bangladesh Office, House 22B, Road 7, Block F, Banani, Dhaka 1213, Bangladesh
[52] University of Akureyri, Borgir v/Nordurslod, IS-600 Akureyri, Iceland
[53] University Santiago de Compostela, Faculty of Political Sciences, Santiago de Compostela, A Coruña 15782, Spain
